# Food security in Roman Palmyra (Syria) in light of paleoclimatological evidence and its historical implications

**DOI:** 10.1371/journal.pone.0273241

**Published:** 2022-09-21

**Authors:** Joan Campmany Jiménez, Iza Romanowska, Rubina Raja, Eivind H. Seland

**Affiliations:** 1 Center for Urban Network Evolutions, Aarhus University, Aarhus, Denmark; 2 Aarhus Institute of Advanced Studies, Aarhus University, Aarhus, Denmark; 3 Department of Archaeology, History, Cultural Studies and Religion, University of Bergen, Bergen, Norway; University at Buffalo - The State University of New York, UNITED STATES

## Abstract

Food security in ancient urban centers is often discussed but rarely formally modelled. Despite its location in an inhospitable desert where food production is a constant challenge ancient Palmyra grew from a small oasis settlement in to a major geopolitical player. Here, we present a spatially explicit reconstruction of the land use and agricultural yield expectations of its hinterland determining the maximum feasible population of the city. Coupling the hinterland carrying capacity model with palaeoclimatic data allowed us to track changes in the food security of the city in the face of changing climate. While initially the hinterland could provide ample food resources for the small settlement with time the deteriorating climate conditions after the Roman Optimum (100 BCE-200 CE) collided with rapidly growing population of the city. The nexus of these two processes fall at mid third century–a period of profound changes in the structure of Palmyrene society, its geopolitical situation and its historical trajectory. The results point to increasingly precarious subsistence levels as a likely factor behind rapid militarization, shift towards an autocratic regime and military expansion of the city in the late third century CE. As a well-established causal mechanism in many modern conflicts and crises, food security is also a potential causal factor behind historical events, if a hard one to prove due to the difficulty of identifying relevant data patterns. The methods presented establishes a robust research pipeline that can be used on other ancient urban centers, contributing to the construction of an empirically supported model of how food security shaped human history, past and present.

## 1. Introduction

Most of the earliest urban centres are found in areas with fertile soils and favourable climate, where the hinterland is capable of producing a significant surplus that could support a high density of population. However, for those cities whose existence was due to other factors, such as trade, resource extraction, craft activity, transport infrastructure or military activity [1–6], ensuring food security, defined as a continuous availability of sufficient and adequate food provision to all members of the community, was a challenge of vital importance. Palmyra is one such city [7]. Located in an inhospitable region of the Syrian Desert, it is often called a “caravan city” due to its dependence on long-distance trade [8, 9]. Palmyra grew thanks to long-distance trade from a small oasis settlement in the first centuries BCE and CE to become a sizable city negotiating its position between the Roman and Parthian, later Sasanian empires. During the first three centuries CE it even briefly challenged Rome during the rule of the city’s famous queen Zenobia. Determining how cities such as Palmyra, located in marginal environments, ensured food security over centuries and in the face of cataclysmic events such as conflicts, social transformations and climate change is of critical importance for our understanding of urban resilience and provides a new and highly relevant perspective.

Even in a prosperous city, that could levy taxes on the passing caravans, food security was not guaranteed through monetary resources alone. The growing population of Palmyra, as evident in the rapidly expanding city plan [10, 11], must have outstripped the food supplied from the oasis itself relatively early on in its history. This created a need for a dedicated food supply and distribution system to ensure food security for the growing urban population. Given the distances involved and the cost of land-based transport of foodstuff through a desert, it is likely that the bulk of food necessary for the city population’s survival had to be cultivated locally.

This makes Palmyra a perfect case study for exploring and reconstructing trends in food security. Its location as an individual large urban center in a sparsely populated area and well documented historical trajectory provide clearly demarcated spatio-temporal boundaries. The city was intensively excavated and researched for more than a century providing a diverse range of evidence types that can be used to contextualise findings [12–17]. Although there had been little focus on Palmyra’s hinterland in early research, more recent work has started to address this gap, providing much needed data about the land surrounding the city [18–20].

While food supply in various ancient cities has been extensively discussed in the past [21], this study is the first to leverage a combination of historical and archaeological sources, traditional humanities methods, agricultural productivity simulation software, geospatial modelling and paleoclimatology. A formal spatially explicit model of land productivity has resulted, allowing us to assess the changes to the hinterland’s carrying capacity over time and through climatic, social and political transitions. This research pipeline is portable to other historical urban centers opening a new avenue for comparative studies on urban resilience.

In this study, we employ historical, ethnographic and archaeological data to guide the application of GIS and agricultural productivity software, thus creating a model of ancient agricultural productivity around Palmyra. This model allows us to calculate robust estimates for the maximum optimistic carrying capacity around Palmyra. Climatic scenarios implied by paleoclimatic data and their implications for food security in Palmyra can be tested in this model. As it sets the maximum feasible population, the carrying capacity of the hinterland can also trace the population evolution over time. Current estimates of Palmyra’s population vary considerably from 10,000 [22, table 4 in 23] to up to 200,000 people [24] and can be narrowed down. Finally, historical analysis highlights events and processes that could have been caused by environmental and demographic pressure of food insecurity. Here, we suggest that food security might have been a factor in the city’s third-century social and socio-economic transformations that have led to the military expansion of Palmyrene rule and the short lived Palmyrene Empire. While food security is often recognized factor in explaining modern phenomena this study highlights its role in historical narrative and provides robust quantitative methods to do so.

### 1.1. Location and environment of Palmyra

**Fig 1:**
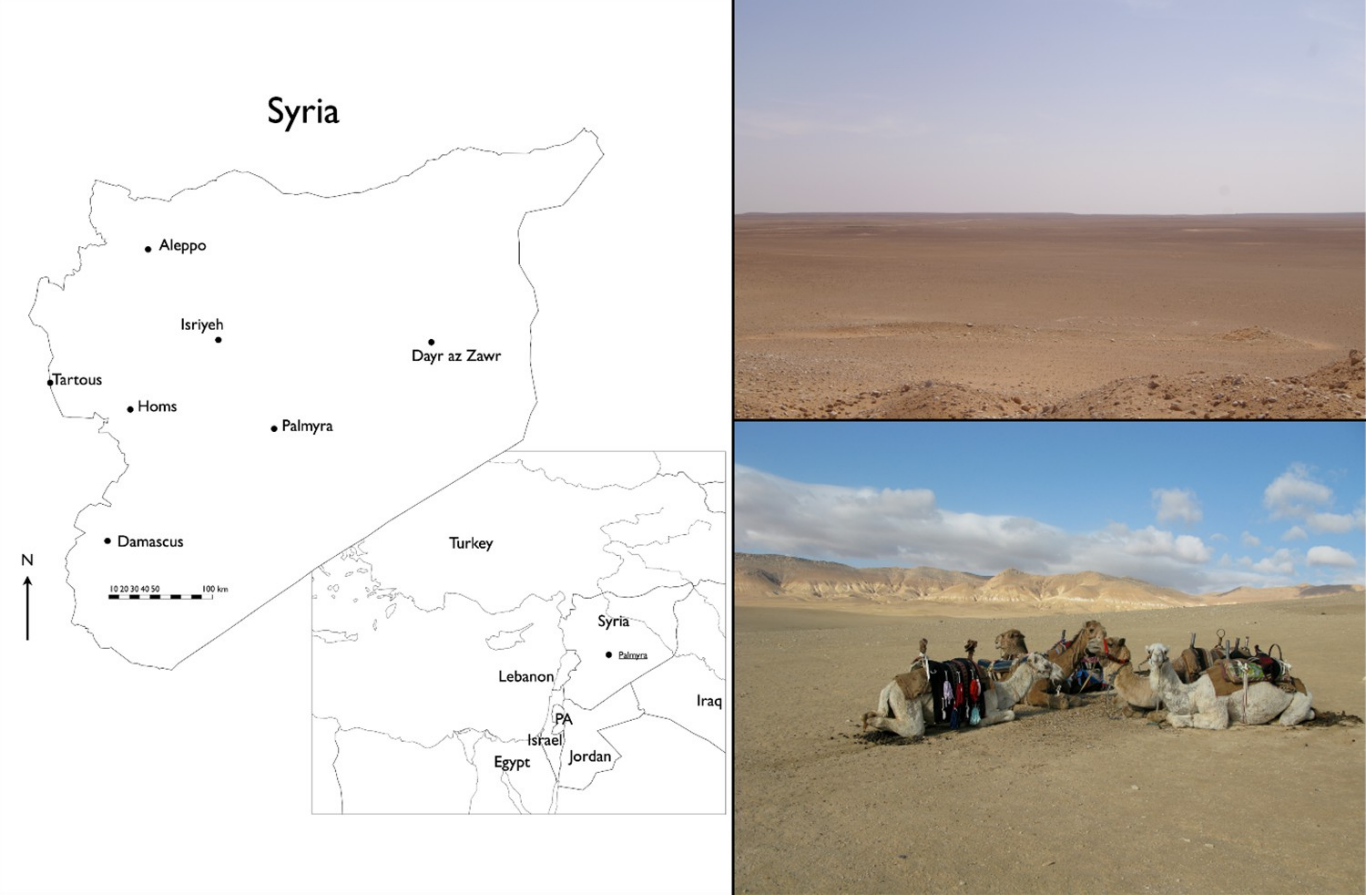
Palmyra’s surroundings. Clockwise starting from the left: Position of Palmyra (Eivind Heldaas Seland), gravel-plains southeast of Palmyra (reproduced with permission from Jørgen Christian Meyer, original copyright 2006), limestone mountains north of Palmyra (Eivind Heldaas Seland).

Palmyra’s location, at an oasis within a vast steppe desert, determined its subsistence base. It lies at 150–200 km distance from perennial rivers and major regions of rain-fed agriculture. The city is located immediately south of a relatively low, yet steep mountain chain running from the outskirts of Damascus northeast towards the Euphrates, and opens to the Syrian Desert from all the remaining sides. It is located in a semi-arid environment, a dry steppe (bādiya) (Fig 1), between isohyets 150 mm and 100 mm with an average rainfall of around 125 mm per year. This is below the standard water requirements for cereals such as barley (200 mm) and wheat (250 mm), and significantly below the traditional limit between agricultural settlement and desert pasture in the arid regions of the Near East. Through most of pre-modern history this limit was closer to 400 mm, a level that ensures sufficient rainfall even in most of the regularly occurring dry years [25]. Thus, while the oasis has been a source of agricultural produce since the Neolithic [26], the area beyond it could not sustain rain-fed agriculture in average years, and was predominantly used to herd sheep, goats, and camels. However, even in arid landscapes like the Syrian desert it is feasible to farm crops if there are adequate water management strategies in place, such as cisterns or dams collecting rainwater in wadis and in larger basins. Such infrastructure is well attested in the hinterland of Palmyra [27].

### 1.2. Food types and city’s subsistence base

Cereals have been the basis of most ancient diets, and barley has been the staple crop in the region due to its lower water requirements. Palmyrene funerary art includes banqueting scenes as do the numerous locally produced clay-tokens known as tesserae, which depict food and drink available in Palmyra, at least at certain points of the year, such as dates, pomegranates, grapes, bread/cakes, wine etc [28–33]. The oasis likely provided various fruits and vegetables, such as olives and dates, but the amounts would have been comparatively small [27]. The Tax Tariff of Palmyra [34, 35] dating to the mid-second century CE provides a list of goods brought into the city from the surrounding hinterland, which includes commodities such as wheat, wine, olive oil, animal fat, sheep, horses, mules, camels, and fodder. Produce from within the Palmyrene’s borders was exempt from taxes, indicating that the city provided incentives for agricultural activities in the area and that such production took place.

### 1.3. The base of the city’s economy

Roman Palmyra is known as one of the examples *par excellence* of a “caravan city” with trade considered its *raison d’être*. Palmyra’s geographical location at the key respite point at the major trade route but also at the frontier between the Roman and Arsacid (Parthian) later Sasanian empires enabled engagement in the lucrative long-distance trade between the Mediterranean and the Indian Ocean both including caravan and overseas trade. Palmyrene merchants operated caravans across the desert between Syria and Mesopotamia by the early first century CE, and from the second century onwards also maritime trade in the Persian Gulf and to the mouth of the Indus. By the third century Palmyrene merchants and ship-owners were also established in Egypt and the Red Sea, venturing into the Gulf of Aden and the western Indian Ocean [36, 37]. Palmyrene merchants settled across the eastern Roman border and held important positions in local communities there [38–40]. Although there is limited evidence regarding the type of goods being traded, it is generally accepted that these were likely to be low-weight, high-value goods, such as silk, aromatics, spices or precious stones, rather than bulky foodstuffs [37, 41, 42]. Palmyra had a textile production sector of some importance [43] and other crafts must have been present to serve the local needs. While under Roman control, Palmyra retained considerable political and fiscal autonomy [44, 45].

### 1.4. Historical outline

The Palmyrene polity seems to have emerged from a tribal framework of the nomadic population in the surrounding desert, facilitating the operation of caravan trade and reducing the need for investment in military presence and infrastructure in the hinterland and along this section of the Roman border [46–50]. The start of the rapid urbanisation of Palmyrene can be traced in archaeological, epigraphic and historical records to the first century BCE [10, 11, 15]. The city grew in both prominence and size during the first and the second century CE and a new city centre was laid out on a monumental scale north of the earliest urban nucleus [10]. Palmyra was integrated into the Roman Empire at an unknown date before the early first century CE [51, 52]. It received the honorary name of Hadriane in connection with emperor Hadrian’s visit to the city 129 CE and officially received the status of a Roman colony at the beginning of the third century during the reign of Septimius Severus (193–211) or Caracalla (211–217 CE) [53, 54]. Throughout this period the population of the city gradually grew. The new district north of the wadi, which comprises the bulk of the presently visible ruins of Palmyra received most trappings of a prosperous Roman city, including an agora (forum) a theatre, several temples a, nymphaeum (fountain), two long colonnaded streets, a bath and perhaps even an amphitheatre [10].

A sudden change came around the mid-third century when Septimius Odaenathus became the ruler, followed by his wife Zenobia. They dramatically expanded Palmyra’s realm so that, at its peak, it stretched from Egypt to Anatolia [55, 56]. After the defeat at the Battle of Edessa, 260 CE, when emperor Valerian was taken captive by the Sassanids, Odaenathus and the Palmyrene army drove the invaders back and sacked the Persian capital of Ctesiphon. This victory and subsequent military campaigns gave Palmyra military control over the eastern part of the Roman world. In 271/272 CE Zenobia even claimed the imperial purple on behalf of her son Vaballathus. Palmyra’s dramatic ascension to hegemony, however, did not last long. A Roman punitive intervention put an end to the Palmyrene empire in 272 CE. After a brief rebellion in 273 the city was sacked. It survived, but reduced to a minor provincial town [57].

During the third quarter of the third century, Palmyra was arguably one of the most important cities of the eastern Roman Empire. The city attracted artists, philosophers, along traders, artisans, soldiers, builders and slaves charged with ensuring the smooth functioning of this urban centre. The large population of the city thrived in the inhospitable environment of the Syrian Desert. It is clear that this city must have had a robust food production and distribution system in its hinterland that ensured its population food security levels necessary to survive grow and thrive. By combining data and methods from several disciplines, we can now reconstruct the subsistence basis of the city, assess the robustness of their food security and track how climatic changes over the first three centuries CE influenced the city’s resilience.

## 2. Materials and methods

To evaluate Palmyra’s hinterland carrying capacity and the maximum threshold for the population of the city the designed research methodology goes through four stages:

- Establishing the spatial range of Palmyra’s hinterland, or its minimum site catchment;
- Calculating the carrying capacity of the farming areas within that spatial range;
- Translation of the carrying capacity into population size;
- Evaluating the temporal fluctuations in the carrying capacity and by extension, the maximum feasible population.

The first phase establishes the area of the city’s hinterland within one, three and five days of camel travel and takes into account both topography and the availability of water sources. The baseline carrying capacity of each distance envelope is then calculated in modern hulled barley yield, and translated into population estimates. While Palmyra’s population consumed foodstuffs beyond barley, the grain, being the most water-efficient crop (and a local staple), represents a good proxy for the maximum calories that can be extracted from the available land.

Once the minimum necessary catchment is identified, we narrow down areas that could be irrigated. Four areas suitable for irrigated agriculture were identified [20], and measured. The availability of water for irrigation, when possible, is calculated for each of these. With the availability of water determining the surface that could be irrigated, the carrying capacity of each agricultural area is estimated for different rainfall levels, also by calculating a barley output and translating it into a population number. Finally, by fitting these yields to climate records we evaluate the changes to the carrying capacity of Palmyra’s hinterland over the three centuries of the city’s history, and therefore the population that could be supported from the local produce. A detailed description of the methodology, including all steps, functions and values used as well as data analysis scripts, intermediate figures and evaluations of alternative sources of data are available in S2–S9 Files. The main datasets used for the analysis are listed in S1 File. A detailed discussion of all the decisions, compromises and limitations of the methodology can be found in a forthcoming publication [58].

### 2.1. Spatial range of Palmyra’s hinterland

**Fig 2:**
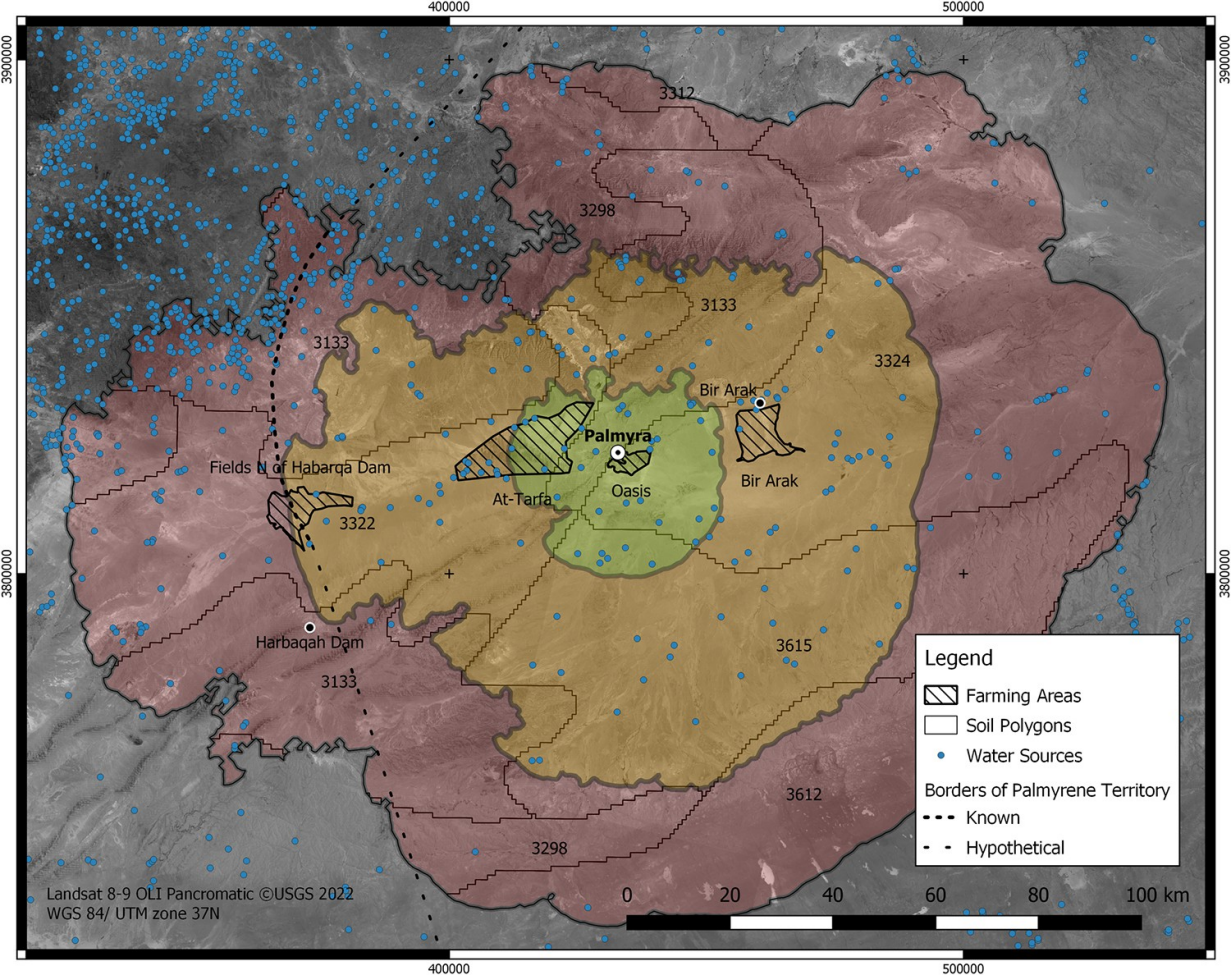
Map of the catchments around Palmyra. It shows areas within one day (green), one to three days (yellow) and five days (red) camel travel from the city. The numbers indicate each soil unit from the HWSD [61]. The striped areas are the maximum extent of fields in each of the main agricultural areas. Landsat 8–9 aerial imagery courtesy of the USGS, SPOT CIB-10 imagery was used for the analysis [62]. Water sources from Seland [63]. Palmyra territory’s boundary from Matthews [34].

The details for this and the next two steps are collected in S2 File. The catchment area of Palmyra is represented as three concentric distance envelopes, which encompass the area of one, three and five days travel from the city (Fig 2). This site-catchment spatial analysis followed standard methods [59, 60], adjusted to capture the specificity of camel movement across the Palmyrene arid landscape. The two main factors used in the cost calculations were topography and water availability.

The slopes were generated from a reprojection of the USGS EROS SRTM 1 arc-second global elevation raster [64–67]. We squared the value of slopes, therefore exaggerating those with the highest values to counteract the well-known issue of the systematic underestimate of the impassibility of steep slopes [68], which is particularly pronounced in the low but steep hills north of Palmyra (Fig 1). We also used the 1/500000 provisional "Goings" map of Syria [69], which displays the difficulty of travel to amend the baseline topographic map with areas deemed impassable. The resulting slope raster was then used as an input for a version of Tobler’s Hiking function [70] modified to work with slope expressed in degrees [71].

Lack of reliable water sources severely impedes the ability of caravans to travel through arid areas such as the Syrian Desert. Thus, cells’ friction was scaled depending on the presence of a water source and the reliability of the water source (spring, well, cistern, etc) [data based on Soviet military maps, collected by E. H. Seland, 63]. Assuming six hours of march per day the calculated distances average to about 30 km on even terrain which is consistent with values known for caravans with pack animals, such as camels [72]. The three-day travel envelope also matches Palmyra’s frontiers, as documented by inscribed stone markers [34, 52] (Fig 2).

### 2.2. Baseline carrying capacity of Palmyra’s hinterland

The details for this step are collected in S3 File. The carrying capacity of the hinterland is based on barley cultivation, which has been the staple crop in the area since the Neolithic thanks to its low water requirements (200 mm). Nevertheless, rain-fed agriculture is not reliable in the Syrian Steppe so traditionally barley is grown on irrigated fields [73]. Other cereal crops or horticulture are feasible in the area, but they require more water. While not arguing that the population of Palmyra sustained itself exclusively on barley [74], it is the most cost-effective cereal in terms of water use and therefore the best proxy for the maximum amount of calories that can be extracted from the available land and water resources.

Crop yields were calculated using the AquaCrop software—a widely used software tool developed by the United Nations Food and Agriculture Organisation for calculating agricultural yields [75], particularly suited for annual cereals in areas where water is the limiting factor [76–78]. The required inputs include soil type, field management strategy, and climate data, such as monthly precipitation, maximum and minimum temperature, evapotranspiration and CO_2_. The soil data were obtained from the FAO/UN Harmonized World Soil Database v. 1.2 [61, 79], which contains the required data on soil compositions and depth. Since there are several soil types within each soil unit, the weighted average of productivity was used as the productivity of the entire soil unit.

Climate data, i.e., precipitation and temperature, was taken from WorldClim 2.1 [80], evapotranspiration data from Brunel et al. [81] and for atmospheric CO_2_ the global average was used. We assumed simple field management strategies, consistent with local agricultural techniques [73], such as weed management, and water management described below for each area. We tested two irrigation levels, 200 or 400 mm, to see if irrigating a smaller area but with more water would be more productive but the total yield was on average three times higher for 200 mm (S3 File, step 3.6). The 1-year fallow cycle was part of traditional cereal agriculture in Syria in the twentieth century [73] and we assume that the same practice was followed in Roman times. The weighted average productivity was multiplied by the area of the corresponding soil unit within each of the three distance envelopes and for each of the four identified agricultural areas (S3 File). This pipeline produced a total output in tonnes of barley under ideal conditions, i.e., consistent amount of rain, no losses to pest infestations, no losses in storage and distribution, and perfectly efficient water distribution.

### 2.3. Agricultural areas in Palmyra’s hinterland

As mentioned, large-scale rain-fed agriculture is not feasible in the hinterland of Palmyra [82]. While opportunistic small-scale rain-fed barley farming was sometimes possible in some wadi beds as a supplementary source of grain, it would not constitute a food production strategy efficient enough to reliably supply a large urban centre. To achieve that, dedicated agricultural areas with water management infrastructure were necessary. Meyer [20] distinguished four main agricultural areas that fall within the three-day travel envelope: the Bir Arak oasis, the At-Tarfa depression, the fields north of Harbaqah and the Oasis of Palmyra (Fig 1). Each of them has archaeological evidence of occupation and agricultural activity during Roman times:

- In Bir Arak, the Qanats and remnants of buildings, as well as an inscription in an altar that mentions “Yaribol (a Palmyrene deity), the irrigator of the earth” [16].
- In At-Tarfa, three Palmyrene altars thank deities for the rain and include agricultural imagery [83].
- A Latin inscription and an analysis of construction phases strongly suggest a Roman origin for the Harbaqah Dam [20, 84–86, Contra 87]. Roman architectural fragments found close to the Umayyad Garden in Qasr al-Heir al-Gharbi, north of the Dam, suggest an earlier Roman occupation [88]. We assume that just like in Umayyad times, the water of the Dam was used for irrigation there.

The mountain range north of Palmyra has been traditionally considered an important source of food for Palmyra [20, 27, 89], and surveys have produced evidence of agricultural estates and small irrigation systems. However, access from Palmyra was difficult due to the topography as indicated by the region falling outside the catchment envelopes (Fig 2). In addition, the evidence suggests only small-scale irrigation and farming there. Large-scale irrigation and agricultural production would have also been difficult in higher areas, due to the difficulty of capturing runoff water and establishing irrigated fields on the steep slopes at any larger scale.

In all of the four areas, traces of contemporary or abandoned fields are clearly visible from aerial imagery. While the extent of ancient fields around Palmyra is unknown, it is safe to assume that the fields opened in the 1960s as part of Bedouin settlement and agricultural development efforts [90] occupied the most favourable lands for farming and represent the maximum practical extent of non-opportunistic cereal agriculture.

Across the region, rainfall needs to be complemented with water management techniques to reach the level required for barley to grow [20]. Evidence for water management in the area indicates that runoff could have been redirected with temporary earthen embankments [91], small walls and dams such as those identified in wadis north of Palmyra [89] and rock-cut channels, or collected in cisterns. Additionally, water could have also been acquired from wells thanks to water-lifting devices, such as animal-powered *Saqiyya* or similar devices, which were fairly common across the Roman world, especially towards the Late Roman period [85, 92, 93]. For Bir Arak and the At-Tarfa depressions, we used their catchment areas to estimate the rainfall available during the growing period. The effectiveness of the water management systems was set to between 5 and 15% for At-Tarfa, and 30% for Bir Arak where a more stable source of water—Qanats are still visible in aerial imagery. The capacity of the Harbaqah Dam of 5,000,000 m3 [20, 27, 94–97] was used for the fields of Habarqah. Finally, the Oasis of Palmyra does not have an estimate of the water that could be available for agriculture before the introduction of water pumps, so its maximum extent irrigated at 200 mm was used as a baseline.

With this input, we simulated the productivity of all four areas. The weighted average productivity of each farming area, calculated in AquaCrop, was multiplied by the irrigated area to give an output in tons of barley. All of the above estimates need to be considered as a “reasonable best-case scenario” and are likely to be overestimating water and land availability for agriculture, thus they represent the maximum feasible carrying capacity of Palmyra’s hinterland.

### 2.4. Population numbers

The translation of barley yields into the number of people that could be sustained is based on the average consumption figures of 360–510 kg of barley/person/year [20]. These values come from Cato the Elder’s *De agri cultura*, where he details cereal requirements for a slave tasked with hard manual labour (*Agr*. *56*). The figures match estimates of yearly consumption for Seleucid peasants made by Aperghis [98]. According to his numbers, 320–370 kg of hulled barley/person/year would be needed for a 1900 kcal a day 100% barley diet rising to 515–590 kg of barley/person/year for a 3000 kcal diet appropriate for manual labourers. 1900 kcal a day is a low caloric intake, and most likely mean intakes were higher. Scheidel and Friesen criticized estimates of 250 kg of wheat equivalent as insufficient for human survival, instead accepting higher estimates around 315 kg [99]. This means that the lower end of our carrying capacity estimates is likely closer to the truth than the upper estimates.

The population size numbers necessarily include the rural population that would have been involved in producing the food destined for the city but which does not necessarily constitute Palmyra’s population. Workforce requirements would have been highest during harvest [73] but the local population might have been aided then by the nomads [100, 101] as the winter crops harvest coincides with the return of pastoralists from the desert to the better watered areas close to agricultural land [73, 102]. There is also evidence in other arid areas, like the Negev, of nomad-settled economic complementarity during the Roman period [103, 104]. While city inhabitants could have also helped the effort, given the distance between the city and the agricultural areas it was unlikely to be a significant addition.

The average labour requirements of harvesting, threshing and winnowing cereal (S6 File) were calculated following the methods by Laabs and Knitter [105] and complemented with the data from the Cologne Tableau [106, 107] a collection of econometric data for traditional economic activities collated from written, ethnographic and experimental sources.

### 2.5. Temporal patterns in the carrying capacity of Palmyra’s hinterland

Additional details for this step are collected in S4 File. The calculated baseline carrying capacity of Palmyra is based on modern rainfall figures. Since the climate has remained broadly similar during the late Holocene [108], we consider it to be an appropriate first approximation. However, the marginal nature of the Palmyrene environment means that even relatively modest fluctuations in water availability could have significant consequences for food production and security. We used paleoclimate data to evaluate the changes to food security in Palmyra during the first three centuries CE.

There are no high-resolution palaeoclimatic sequences for the area immediately surrounding the city, and climate proxies in the northern Levant, especially in the interior are limited [see 108 fig. 1,4,^109^ fig. 2]. We evaluated 24 distinct climate records from the wider region of the East Mediterranean. Most of them did not have the decades-long resolution or their values were affected by stochastic annual variations, while others mapped multiple factors making disentangling the climate proxies impossible (S4 File).

The Dead Sea rainfall records based on the reconstruction of the ancient shoreline [110, 111] is the most representative of the rainfall in the region of Palmyra because of its proximity and quality of the record. The Dead Sea is fed from a large catchment area, thus representing an averaged record of regional rainfall. While annual variation in the lake level occurs, the sedimentological record accumulates slowly, so it smooths these short-term changes thus making the reconstructed lake levels a reliable proxy for decadal and centennial trends in regional rainfall [110]. To corroborate the patterns against a higher resolution but more localised dataset the Dead Sea trends were contrasted against isotope data from speleothems in Soreq cave, Israel [112] and against several larger scale, long-term climate records from outside of the region (S4 File).

The Dead Sea sequence was then calibrated using 20-century precipitation data from the Palmyra Weather Station (S3 File, step 3.8; S9 File, step 23) [113] to extrapolate the average and standard deviation of rainfall in the Palmyra’s area during the first three centuries CE from the mean rainfall in the Dead Sea.

### 2.6. Data caveats

Several factors, currently difficult to estimate, could narrow our estimates down (but not up), in particular, the area under cultivation, fluctuations in rainfall and losses during distribution. First, the area assumed here as under possible cultivation was assigned generously and is thus much larger than the area farmed at present. Remote sensing and field survey could narrow the extent of arable land further.

Second, yearly fluctuations in rainfall are not accounted for in the model. They are significant in the region and limit the reliability of food production [82, 113] requiring storage solutions. There are no known large storehouses for grain (*horrea*) in Palmyra before 273 CE similar to those found in Rome, Ostia, and elsewhere. Traditional elite dwellings in the Syrian countryside, however, incorporate large storage rooms for grain, and the so-called khans (caravanserai) could have also included grain storage spaces [73]. The countryside around Palmyra holds a large number of such structures visible in aerial photographs and identified through archaeological surveys and excavation [15, 114]. Further research is needed, however, to better establish their possible function and role in the city’s food storage and supply system. Similarly, storage for seed would have been required, but due to the productivity of the area the amounts might have been as low as 50 kg/ha, 2,5% of the total grain, or even lower [73, 115, 116].

Third, the model assumes no losses during the distribution of grain. Traditional grain management, storage and transport under premodern conditions lead to considerable loss due to decomposition, parasites and pests. For example, in twentieth century Ethiopia this amounted to 25–38% of the harvest [117]. These losses depend on the methods and length of storage, climatic conditions, and other factors currently difficult to estimate for Roman Palmyra. However, these losses would have further lowered the amount of people that could be reliably fed in Palmyra. As stated above, a usual loss rate of 30% would have had a proportional impact on the city’s carrying capacity.

On the other hand, a higher population could be sustained if alternative sources of food were used. As discussed above, systematic large-scale long-distance transport of bulk foodstuffs over land from the mountains or the Orontes river Valley is unlikely due to its prohibitive cost but cannot entirely be ruled out, as these areas could have complemented local production in bad years [20, 27, 89, 118–120]. Alternatively, opportunistic dry farming in wet years could support grain production, however, at a significant risk due to the unpredictability of rainfall. We know that Palmyrenes had a more diverse diet than simply barley, including meat and animal products provided by nomads. However, they were unlikely to be given freely or without compensation. The exchange of animal products for grain is a common practice between farmers and pastoralists [121], thus not changing our estimates in a significant way.

## 3. Results

### 3.1. Reasonable maximum population of Palmyra

**Fig 3:**
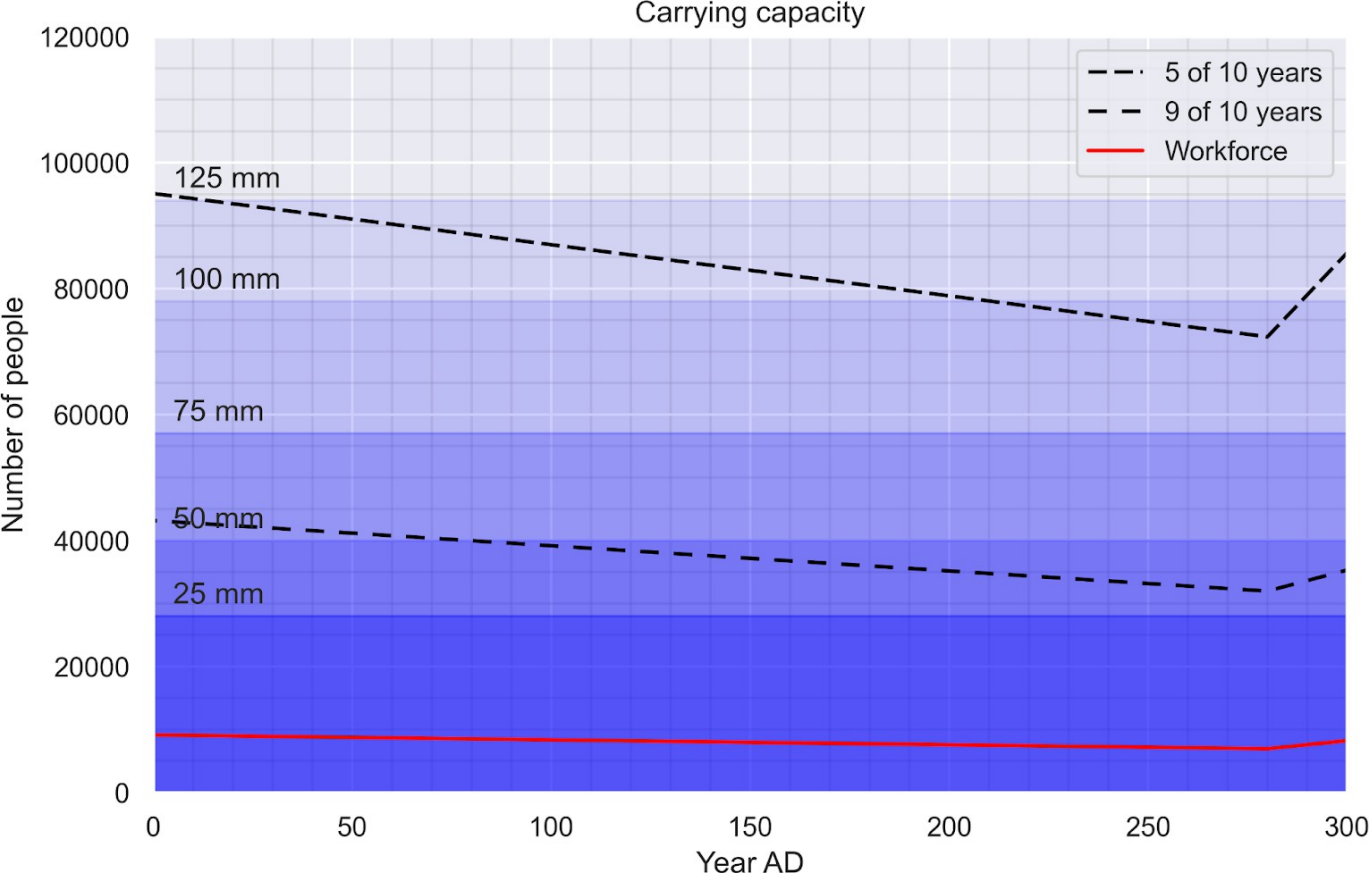
Changes in the carrying capacity between 1–300 CE. In the background, average rainfall levels needed to feed different population levels. Dashed lines show the decadal frequency of successful crop yields.

The results indicate that a population of between 30,000 people could be comfortably supported by the hinterland of Palmyra at all times during its history (Fig 3). The maximum viable population falls between 65,000 and 90,000 (Table 1) people and assumes significant storage infrastructure to ensure food security despite annual fluctuations in agricultural production.

**Table 1:** Estimates of the maximum possible population fed in optimal conditions.

Area	Barley (tonnes)	Population
*At-Tarfa*	19,951	39,119–55,419
*Harbaqah Dam*	7656	15,011–21,266
*Bir Arak*	3528	6918–9801
*Oasis Palmyra*	2047	4014–5687
***TOTAL***	**33,183**	**65,064–92,174**

The numbers assume modern average yearly rainfall for each agricultural area.

While the soils in the immediate surroundings of Palmyra are highly fertile and could theoretically support hundreds of thousands of people (see S3 File), the available rainfall (on average 125 mm) is a strongly limiting factor restricting the land use to irrigable areas only. In the area within a day from Palmyra, only 13% of the land irrigated practically, and that would not be enough to feed a moderately large Roman city reliably. Within the boundaries of Palmyra’s territory these are available at the distance of up to three days’ travel from the city. This is a significantly larger hinterland compared to most ancient cities where agricultural hinterland was defined by the distance farmers could commute in a day [122]. Historical data also shows the limits on food transport. In eighteenth century India the correlation of grain prices between urban markets decreased markedly with distances over 35 km and was very weak above 70 km [123]. During the eighteenth century famine in Syrian Aleppo in the same period, market mechanisms were insufficient to supply the city from regions beyond its regular hinterland forcing authorities to bring in subsidized supplies from further afield [124]. While several Roman cities required food to be brought in from far away, all had maritime or fluvial ports. In contrast, the city’s isolated location forced Palmyrenes to develop an exceptional food security strategy of exploiting agricultural opportunities further afield.

The city depended on the four irrigated agricultural areas identified by Meyer [20] of The Palmyra Oasis, the At-Tarfa Depression, the Bir Arak Oasis, and the fields north of the Harbaqah Dam (Fig 2). The most productive area is the At-Tarfa depression (Table 1), still farmed extensively in the present day. It occupies a low point east of Palmyra and collects most of the runoff from the mountains north of the city. The Harbaqah Dam could store substantial amounts of water to serve fields of cereals [20]. The oases of Bir Arak and Palmyra had a lower potential for grain agriculture but thanks to the continuous and reliable water supply could be dedicated to gardens and tree groves. Measured in calories these crops outputs are less water-efficient than barley. Altogether, the four agricultural areas could produce around 33 thousand tonnes of barley a year, which could feed between 65,000 and 92,000 people. These yield outputs are in line with previous estimates for agricultural productivity in the ancient world [125; S7 File].

Included in this number is the agricultural workforce. Approximately 8800 labourers would be necessary during harvest (Table 2). With 17,587 hectares farmed this is around 0.50 workers per hectare, which is the upper limit of the range (0.33–0.5 people per ha) estimated for the workforce involved in food production around Pergamon by Laabs and Knitter [105]. Nomadic pastoralists aiding during the harvest could decrease this number [100, 101] but if other, more labour-intensive crops were also grown, the workforce requirements were higher.

**Table 2:** Estimated maximum workforce requirements.

Area	Labourers Harvest	Labourers Threshing	Labourers Winnowing	Total
*At-Tarfa*	1059	2898	1357	5314
*Harbaqah Dam*	411	1112	521	2044
*Bir Arak*	197	513	240	950
*Oasis Palmyra*	116	297	139	552
***TOTAL***	**1783**	**4820**	**2257**	**8860**

The numbers assume optimal conditions, with a modern average yearly rainfall, for each agricultural area.

**Fig 4:**
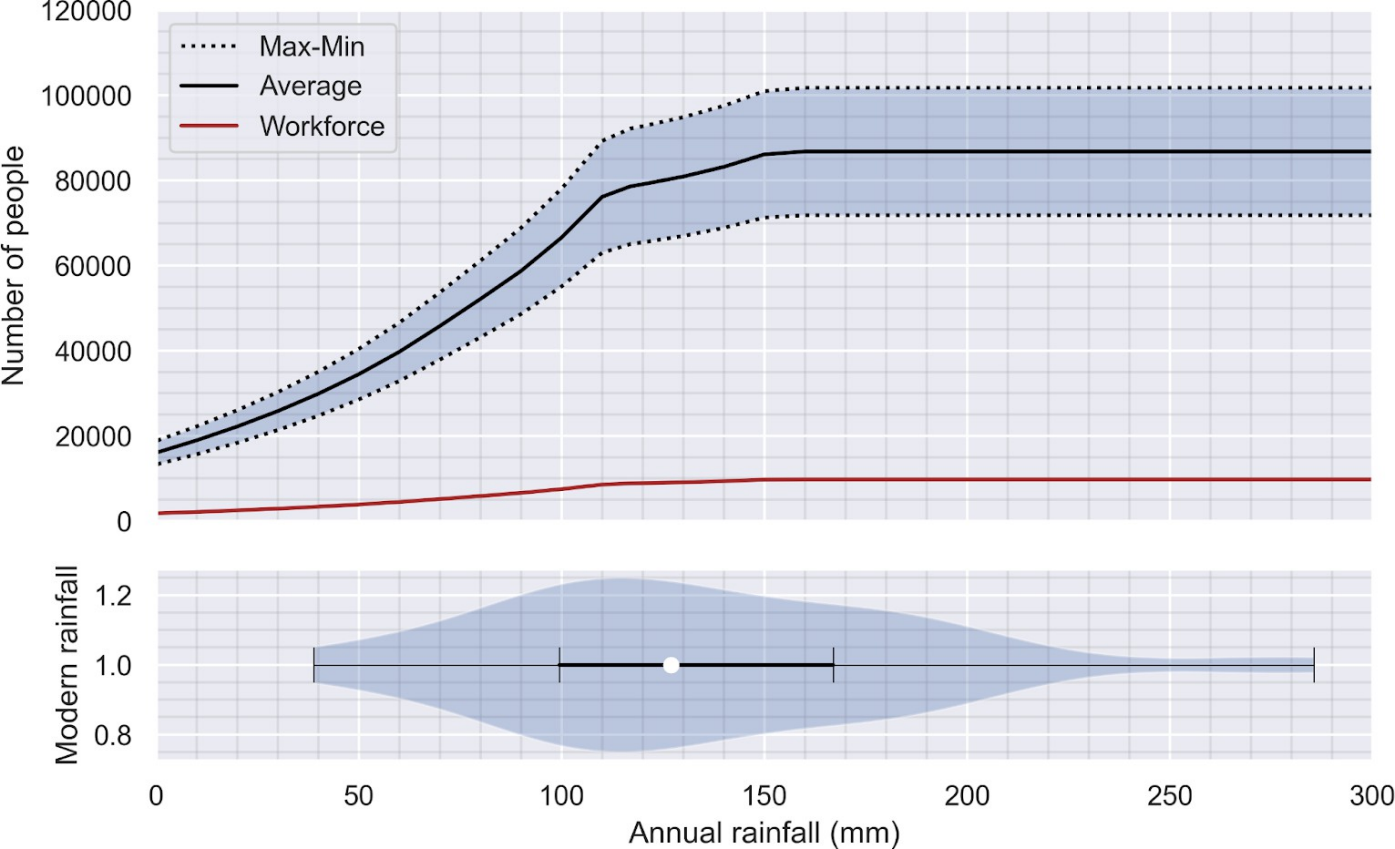
Carrying capacity in relation to rainfall. Top: the relationship between population numbers and yearly rainfall levels. Bottom: a violin plot showing the distribution of yearly rainfall between 1934 and 1999 shows the range of feasible population sizes give twentieth century levels of rainfall [113].

The reported numbers of 33,000 tonnes of barley, and 65,000 to 90,000 people, represents the maximum reasonable estimate for food production in the hinterland of Palmyra, with a 6000–9000 strong agricultural workforce. The extent of irrigable land places a hard limit on food production meaning that additional rainfall would result in surplus food production only up to the threshold of 90,000 people (Fig 4). Nevertheless, more efficient water management strategies could increase the security of the food supply and the diversity of foodstuffs available to the local population.

### 3.2. Food security over time

All of the above calculations are based on WorldClim’s 30-year average rainfalls. However, the region experiences significant annual variation in rainfall (Fig 4) [113]. The highest level of food production, enough to support 90,000 people, is feasible on average five out of every ten years. Reliable food security (nine out of every ten years) is achievable for a population of up to 45,000 people, including 6000 agricultural workers. Paleoclimatic records, however, indicate that the amount of rainfall in the early first Millennium CE would have been lower than these figures [111].

**Fig 5:**
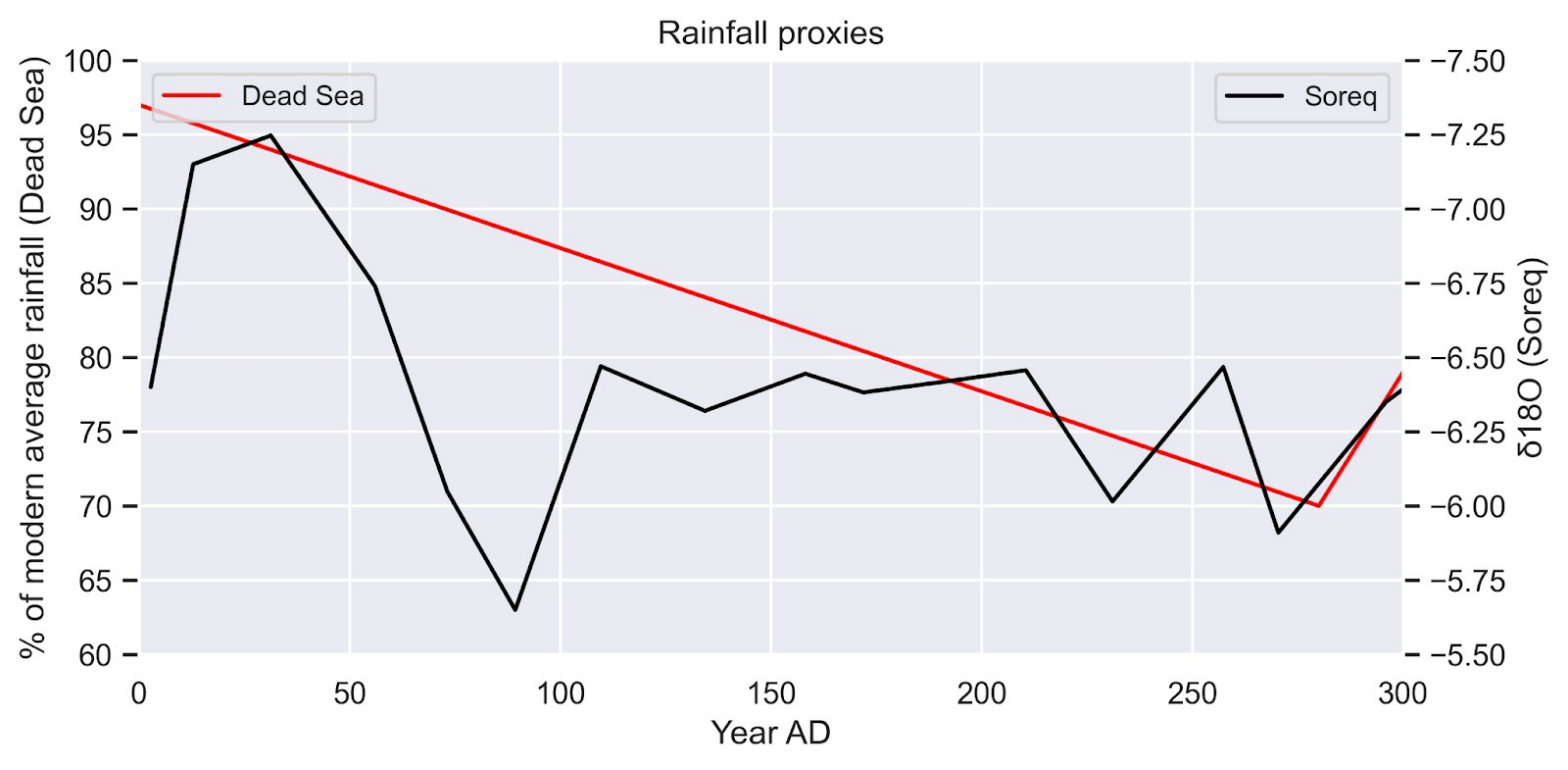
Graph of rainfall proxies. Shown are the reconstructed rainfall over the Dead Sea (table 4 in 111) and the 20-year rolling mean of Oxygen isotopes in Soreq [112]. Note the truncation of the y-axis, which starts at 60% of modern values.

Rainfall reconstructions in the Dead Sea show a steady decrease in precipitation between the first and late third centuries. The lowest point falls in the second part of the third century during which the rainfall in the region fell below 70% of the modern levels. This is corroborated by a higher resolution but a more localised record of the Soreq cave although it also documents a significant dip in precipitation at the end of the first century, not noted in the Dead Sea records (Fig 5).

Recent work on historical climate-society interaction warns against assuming uniform climate periods and similar effects in different regions [126]. The trend of a generally drier but also more variable climate in the period following the so-called Roman Climate Optimum (200 BCE—150 CE) has, however, been documented across the northern hemisphere and in the region through several paleoclimatic (tree ring, ice cores, lake and other geological sequences) and historical sources [109, 127, 128]. For example, the Talmud [Yerushalmi Ta’ anit 3.4; Bavli Ta’anit 24a, as collected in 129] mentions a string of severe droughts that affected Palestine between 210–220 CE, 220–240 CE and 255–270 CE [130]. A dry event has been recorded around 250 CE in other palaeoclimatic records in Anatolia, such as Bereket Basin [131, 132] and the Gravgaz marsh [133]. It is uncertain whether the rebounce from dry to wetter conditions occurred in the fourth or fifth century, but the pattern of prolonged droughts and dry conditions dominating the Roman East in the third century CE is well-documented [126]. While keeping in mind that the aforementioned climatic data do not come from the immediate surroundings of Palmyra, and that the relationship between climate change and agricultural production is complex [134, 135] it is clear that any climatic downturn in such a marginal environment would have consequences for food security.

The paleodata indicates that while at the beginning of the first century up to 95,000 could be sustained by Palmyra’s hinterland five out of ten years, this number drops to 72,000 in the second half of the third century. In terms of robust levels of food security, Palmyra’s hinterland could easily support 43,000 people nine out of every ten years at the beginning of the city’s history, but by the mid third century this number dropped to 31,000, including the rural workforce. While the exact values may be contested given the inherent uncertainty over several factors used in the model (e.g., if storage and transport losses are included all of the above figures need to be reduced by 30%), the overall trend is clear: the capacity to ensure food security in the city would gradually decrease to values over 25% lower than the starting point (Fig 3). This process would have reached the critical point around the mid-third century when the city’s population was supposedly the largest while the hinterland’s food production was at its lowest.

## 4. Discussion

While earlier estimates of the size of the population of Palmyra ranged from 11,000 to 200,000 people [23, 24, 27], a smart subsistence strategy, with extensive water management and infrastructure, could support a population of up to 45,000 for most of the city’s history. Assuming 30% of losses due to storage and transport, this number could be revised down to a maximum of 30,000 people.

Palmyra presents a particular case in terms of sourcing foodstuff through long-distance import. Bulk foodstuff transport overland was expensive in the ancient world [136], and even more so over difficult terrain and large distances at the edge of the empire. The economic feasibility of moving low-price commodities to sustain thousands of people over decades was limited. Thus, the food security of Palmyra was intricately linked to the productivity of its hinterland. During the first two centuries CE, the city could depend on the hinterland food production capacity considerably exceeding the city’s needs. This would have allowed the city to rapidly grow, and invest in urban expansion, monumental architecture and other reflections of considerable disposable income [38]. Nevertheless, at the same time as the population increased, the hinterland’s ability to feed the ever-growing number of people gradually decreased along with the climatic downturn. This gradual process culminated during the middle decades of the third century—a key period for socio-political shifts in Palmyra.

A dramatic change in the administrative framework of the city, likely mirroring a social transition, took place in the mid-third century. Through the first two centuries CE, Palmyra had been ruled according to the common pattern in the Roman East, by a council, a citizens’ assembly and a corps of elected magistrates [137]. Traditional, lineage-based authority certainly existed parallel to the civic structures and likely influenced their operation to a considerable extent [46, 48, 50]. In the course of the third century, however, several aristocratic families were able to monopolise power, and Septimius Odaenathus (ruled c.240-267) became the first lord (*rsˇ’*) and later king (*mlk*) of Palmyra, followed by his widow Zenobia, who ruled until 272 in the name of their under-age son Vaballathus [55, 56, 138]. The “one ruler” regime was a clear departure from the socio-administrative framework based on tribal hierarchies the Palmyrenes had for centuries.

The “caravan city” has also become highly militarized in the third century CE. Beforehand Palmyra operated within the framework of the empire, occasionally supporting Roman troops with military contingents. But during the third century its military capacity increased dramatically [55]. By 260 CE, after the defeat of the Roman imperial army by the Sassanians, the Palmyrene army was able to beat back the invaders, even sacking the Persian capital. The Palmyrene army was later able to quickly take over large parts of the Roman east [139, 140], which the Roman army at the time could not contest. The city’s military confidence was so high that Zenobia even suggested joint rule between her son Vaballathus and the new emperor Aurelian (270–275 CE). The caravan city was then acting like a military power on its own right, able to challenge (if only briefly) Rome itself.

The rise and fall of the Palmyrene’s Empire fall on the key period when the growing population of the city coincided with a decrease in the hinterland food production capacity and an increase in climatic variability. The repercussions for food security of this climatic shift would have been significant. The lowest point in rainfall availability is difficult to pinpoint down to a year, given the resolution of the palaeoclimatic data but it is certain to be extremely low during the mid and late third century. The fragility of Palmyra’s food supply would have forced the city to develop alternative strategies to deal with food security. It would have been a powerful incentive to take drastic and unprecedented action: the election of Odaenathus as *Ras* and the mobilization of an army to fight off the Sassanians and stabilise the region. Ensuring that the city’s main source of income, long-distance trade, could go on uninterrupted, and to supply the city with grain beyond what was possible through regular trade, by tribute and taxation, might have been important priorities for Odaenathus. As the crisis persisted, Zenobia’s grab of imperial power might have been motivated not only by personal and dynastic ambition but also by the need to secure the continued prosperity of her city in face of environmental pressure. While it is not possible to prove a direct causal link between the historical events of the third century and an increased pressure on food security, it provides a compelling counter narrative that what is regarded as Palmyra’s golden age might have been anything but. Being chronically vulnerable to food shortages due to its isolated location in a marginal environment the city might have found itself in a desperate spot, which triggered an unprecedented reaction leading to historical events as described in written sources.

## 5. Conclusions

Palmyra’s location in the middle of a climatically and landscape-wise inhospitable semi-arid zone was dictated by its role in the trading routes connecting the Mediterranean world with the East. Nevertheless, it is an impressive feat of human ingenuity and societal resilience that it grew into a sizable urban centre with enough wealth and power to challenge the dominating geopolitical forces of the time.

Here we explored the effects of changes in climate, and more specifically, rainfall variation on the food security of this desert city and possible implications to its historical trajectory. As discussed, the currently available paleoclimatic evidence is not ideal, and higher resolution palaeoclimatic proxies closer to Palmyra would make a more firm assessment possible. That said, the evaluation of the most reliable nearby proxies, the Dead Sea and the Soreq Cave, in conjunction with general regional trends, suggests a pattern of decreasing rainfall between the first and third centuries, with a possible low point around the mid third century CE. These changes would significantly affect Palmyra’s subsistence base and the city’s food security, and our model suggests that the culmination of this process would have coincided with socio-political changes and historical events such as the Persian invasion. If that happened, it would have placed significant stress on the city and could have been a factor in its militarization and the shift toward autocratic political regime. Causal inference linking of Palmyra’s historical trajectory with climatic change disrupting the city’s food security remains provisional until more lines of evidence converge, but it provides a new line of reasoning and pushes the limits of the conversation forward.

This formal modelling of the agricultural productivity of Palmyra’s hinterland provides the upper limits of what was realistically possible in terms of the population size. Earlier estimates of up to 200,000 people are demonstrably unfeasible, and it is unlikely that a population of 90,000 could be sustained over a long period. However, it also shows the viability of feeding a population of up to 45,000 people given adequate water management and infrastructure and favourable climate conditions.

On the methodological ground, we now have a formal, explicitly defined model of agricultural production, which allows us to quantify food production in the region. On this basis, hypotheses can be tested. How would an Umayyad dating of the Harbaqah Dam affect the food supply for Roman Palmyra? How would a lower water collection efficiency affect agriculture in At-Tarfa? How could small scale run-off irrigation in the numerous wadis in the Syrian Desert help supply the city? These are now new avenues for investigation, made possible by formal modelling and we encourage researchers to take advantage of the data and scripts published alongside this paper.

While for any urban system in antiquity food availability was a key factor in their sustainability, it is also the key consideration for many modern agglomerations. If the required food cannot be grown locally, it has to be brought from outside, which is much facilitated nowadays thanks to modern means of transport but nevertheless imposes a cost and infrastructure consideration. Food security and environmental change have been repeatedly linked to civil unrest, social transformations and conflict in numerous modern contexts. Here, we provide means of evaluating its role in ancient and historical phenomena. Being chronically vulnerable to food shortages due to its isolated location in a marginal environment Palmyra might have found itself in a desperate spot, which triggered an unprecedented reaction leading to rapid military expansion and subsequent collapse as described in written sources. While this scenario has been developed based on data from a particular ancient city, short lived, so-called “boom-and-bust” historical trajectories are well documented for many communities across the world and history. This research pipeline provides a tool for establishing whether food security could have played a role in such historical events. Opening such comparative potential of historical and archaeological data would have far-reaching impact on our ability to reveal social and political consequences of climate change, sources of urban resilience or the lack of thereof and the mechanisms relating food security to conflict.

## Supporting information

S1 File Collected list of the main datasets employed in the analysis. It contains references, access dates, and access links.
(DOCX)

S2 File Detailed steps for the creation of the site catchments (cost envelopes). It contains detailed instructions, as well as screenshots of the process and relevant references.
(DOCX)

S3 File Detailed steps for the creation of the hinterland carrying capacity model. It contains detailed instructions, as well as screenshots of the process and relevant references.
(DOCX)

S4 File Details of reviewed rainfall proxies. It contains a short discussion on climatic proxies for the area of study.
(DOCX)

S5 File Hinterland model, together with results at different rainfall levels. It contains the finished model, which can be used to calculate outputs simply by introducing an average yearly palmyrene rainfall level in mm. It also contains the main excel tables with agricultural productivity calculations extracted from AquaCrop.
(XLSX)

S6 File Workforce requirements from ethnographic, historical and experimental sources. It contains the averages employed to calculate workforce requirements, following the methodology employed by Laabs and Knitter (2021).
(XLSX)

S7 File Yield estimates for ancient agriculture. It contains a collection of ancient agricultural productivity as found in archaeological literature. Mostly collected from Solonakis (2017).
(XLSX)

S8 File Data file with the results of the model. The upper and lower carrying capacity, as well as the workforce requirements for different rainfall levels are included. The file is prepared for use with the S9 File.ipynb script.
(TXT)

S9 File Python script with data analysis. The file is prepared for use with Jupyter Notebook. Data analysis for climate proxies and estimates of carrying capacity over time.
(IPYNB)

S10 File (ZIP)
