# Supplementary material for: Food security in Roman Palmyra (Syria) in light of paleoclimatological evidence and its historical implications
Source: PLoS One. 2022 Sep 21;17(9):e0273241. doi: 10.1371/journal.pone.0273241 (PMC9491547; doi:10.1371/journal.pone.0273241)
Supplement: S1 File — It contains references, access dates, and access links. (DOCX) [file pone.0273241.s001.docx]

Datasets used in the study

The digital elevation model used for our model is the Shuttle Radar Topography Mission (SRTM) 1 Arc-Second Global elevation raster from USGS’ Earth Explorer (<https://earthexplorer.usgs.gov/>), accessed in 07/11/2020. The dataset is available at <https://doi.org/10.5066/F7PR7TFT>.

The aerial imagery used for our model is the SPOT Controlled Image Base 10 meters (CIB-10) from USGS’ Earth Explorer (<https://earthexplorer.usgs.gov/>), accessed in 21/05/2021. The dataset is available at <https://doi.org/10.5066/F7DB809V>.

The Syrian Desert Water Sources dataset was compiled by Seland (2019) and published in DataverseNO (<https://dataverse.no/>), and was accessed in 24/11/2020. The dataset is available at <https://doi.org/10.18710/CEY9QR>.

The location of Palmyra was taken from John W. Hanson’s Roman Cities Database (2016), published in OxREP’s webpage (<http://oxrep.classics.ox.ac.uk/databases/cities/>) and accessed in 24/11/2020. The dataset is available at <https://doi.org/10.5287/bodleian:eqapevAn8>.

Impassable areas were taken from the “Levant 1/500 000 Provisional "Goings" Overprint” (1942), accessed in 08/11/2020. The dataset is available at <http://nla.gov.au/nla.obj-2237684527>.

The “Country administrative areas (boundaries) of Syria” (version 1.0) was downloaded from DivaGIS (<https://www.diva-gis.org/gdata>) and it was accessed in 11/11/2020.

Our soil data came from the Harmonized World Soil Database (version 1.2), published in FAO’s webpage (<https://www.fao.org/soils-portal/data-hub/soil-maps-and-databases/harmonized-world-soil-database-v12/en/>), accessed in 17/05/2021.

Our 10 arc-minute geographic climate rasters (monthly rainfalls and monthly minimum and maximum temperatures) came from WorldClim (version 2.1), published in its webpage (<https://www.worldclim.org/data/worldclim21.html>) and accessed in 14/07/2021.

Historical climate data for Palmyra was collected from NOAA’s Global Summary of the Year (GSOY), Version 1, published in NOAA’s website (<https://www.ncdc.noaa.gov/cdo-web/datasets/GSOY/stations/GHCND:SY000040061/detail>) and accessed in 15/04/2021. The data originally comes in an unwieldy format, and was rearranged in five columns: DATE (the year), PRCP (annual rainfall), TAVG (yearly average temperature), TMAX, and TMIN (maximum and minimum yearly temperatures respectively).

Rainfall reconstructions above the Dead Sea were obtained from Morin, Efrat, Tamar Ryb, Ittai Gavrieli, and Yehouda Enzel. 2019. ‘Mean, Variance, and Trends of Levant Precipitation over the Past 4500 Years from Reconstructed Dead Sea Levels and Stochastic Modeling’, Quaternary Research, 91.2 (Cambridge University Press): 751–67. It was accessed in 23/06/2021. The data for our graphs was collected from table 4, page 761. The data was organized in three columns: “year”, that being the initial year of the interval; “rain”, or the initial MAP, and “%”, or the percentage of the 779 mm modern annual average rainfall. The article is available at <https://doi.org/10.1017/qua.2018.98>.

The Soreq cave speleothem data was collected from NOAA (<https://www.ncei.noaa.gov/access/paleo-search/study/23098>), and comes from Orland, Ian J, Miryam Bar-Matthews, Noriko T Kita, Avner Ayalon, Alan Matthews, and others. 2009. ‘Climate Deterioration in the Eastern Mediterranean as Revealed by Ion Microprobe Analysis of a Speleothem That Grew from 2.2 to 0.9 Ka in Soreq Cave, Israel’, Quaternary Research, 71.1: 27–35. It was accessed in 28/06/2021. The columns were renamed “age_yrBP”, “d18O_VPDB”, and “d18O_VPDB_err” to simplify coding.
