# Supplementary material for: Food security in Roman Palmyra (Syria) in light of paleoclimatological evidence and its historical implications
Source: PLoS One. 2022 Sep 21;17(9):e0273241. doi: 10.1371/journal.pone.0273241 (PMC9491547; doi:10.1371/journal.pone.0273241)
Supplement: S2 File — It contains detailed instructions, as well as screenshots of the process and relevant references. (DOCX) [file pone.0273241.s002.docx]

Version of QGIS: 3.10.11”A Coruña”, Grass

Version of operating system: Microsoft Windows 10 Education, Version 10.0.19041, Build 19041

QGis plug-ins:

- DB Manager (0.1.20)
- Fonctions SIGMOÉ (3.0.1)
- Georeferencer GDAL (3.1.9)
- MetaSearch Catalog Client (0.3.5)
- Processing (2.12.99)


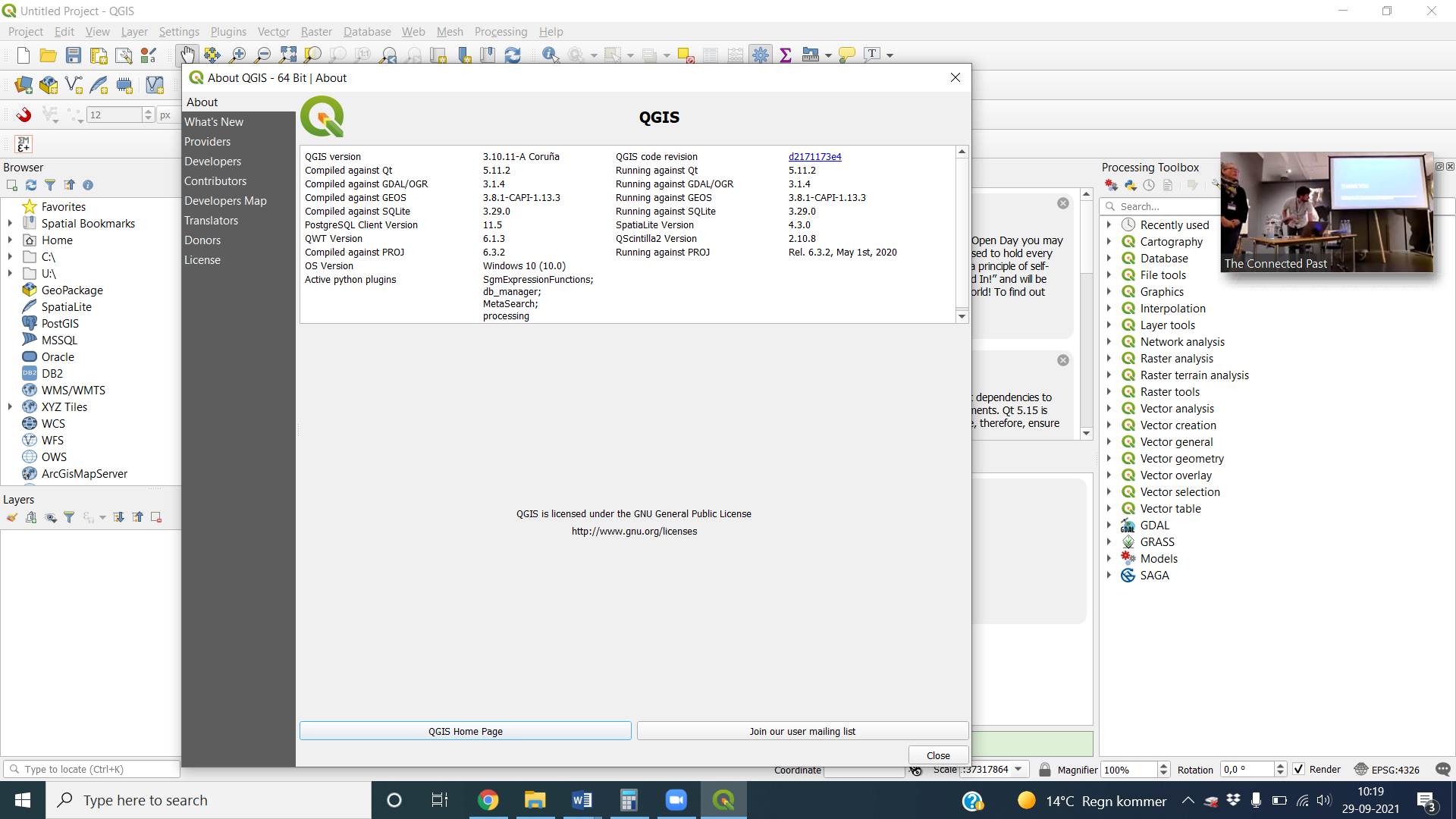


**Steps**

***Generating all inputs***

1. **Project > Properties … > CRS > UTM zone 37N**
2. Get the **Shuttle Radar Topography Mission (SRTM) 1 Arc-Second Global elevation raster** (1) from USGS’ Earth Explorer (https://earthexplorer.usgs.gov/). Download the rasters n33_e035_1arc_v3, n33_e036_1arc_v3, n33_e037_1arc_v3, n33_e038_1arc_v3, n33_e039_1arc_v3, n33_e040_1arc_v3, n34_e035_1arc_v3, n34_e036_1arc_v3, n34_e037_1arc_v3, n34_e038_1arc_v3, n34_e039_1arc_v3, n34_e040_1arc_v3, n34_e041_1arc_v3, n35_e035_1arc_v3, n35_e036_1arc_v3, n35_e037_1arc_v3, n35_e038_1arc_v3, n35_e039_1arc_v3, n35_e040_1arc_v3, n35_e041_1arc_v3, n36_e035_1arc_v3, n36_e036_1arc_v3, n36_e037_1arc_v3, n36_e038_1arc_v3, n36_e039_1arc_v3, n36_e040_1arc_v3 and n36_e041_1arc_v3. Add them to the map by going to **Layer> Add Layer> Add Raster Layer**… Source type is “File”.

**Process: Processing Toolbox> GDAL> Raster Miscellaneous> Merge (gdal:merge)**

**Inputs:** n33_e035_1arc_v3, n33_e036_1arc_v3, n33_e037_1arc_v3, n33_e038_1arc_v3, n33_e039_1arc_v3, n33_e040_1arc_v3, n34_e035_1arc_v3, n34_e036_1arc_v3, n34_e037_1arc_v3, n34_e038_1arc_v3, n34_e039_1arc_v3, n34_e040_1arc_v3, n34_e041_1arc_v3, n35_e035_1arc_v3, n35_e036_1arc_v3, n35_e037_1arc_v3, n35_e038_1arc_v3, n35_e039_1arc_v3, n35_e040_1arc_v3, n35_e041_1arc_v3, n36_e035_1arc_v3, n36_e036_1arc_v3, n36_e037_1arc_v3, n36_e038_1arc_v3, n36_e039_1arc_v3, n36_e040_1arc_v3 and n36_e041_1arc_v3

**Parameters:**

- **Output data type:** Float32
- **Input data value to treat as “nodata” (optional):** not set
- **Assign specified “nodata” value to output (optional):** not set
- **Additional creation options (optional):** Leave blank (Profile: Default)
- **Additional command-line parameters (optional):** Leave blank

**Output:** Combined_DEM_Syria.tiff

**
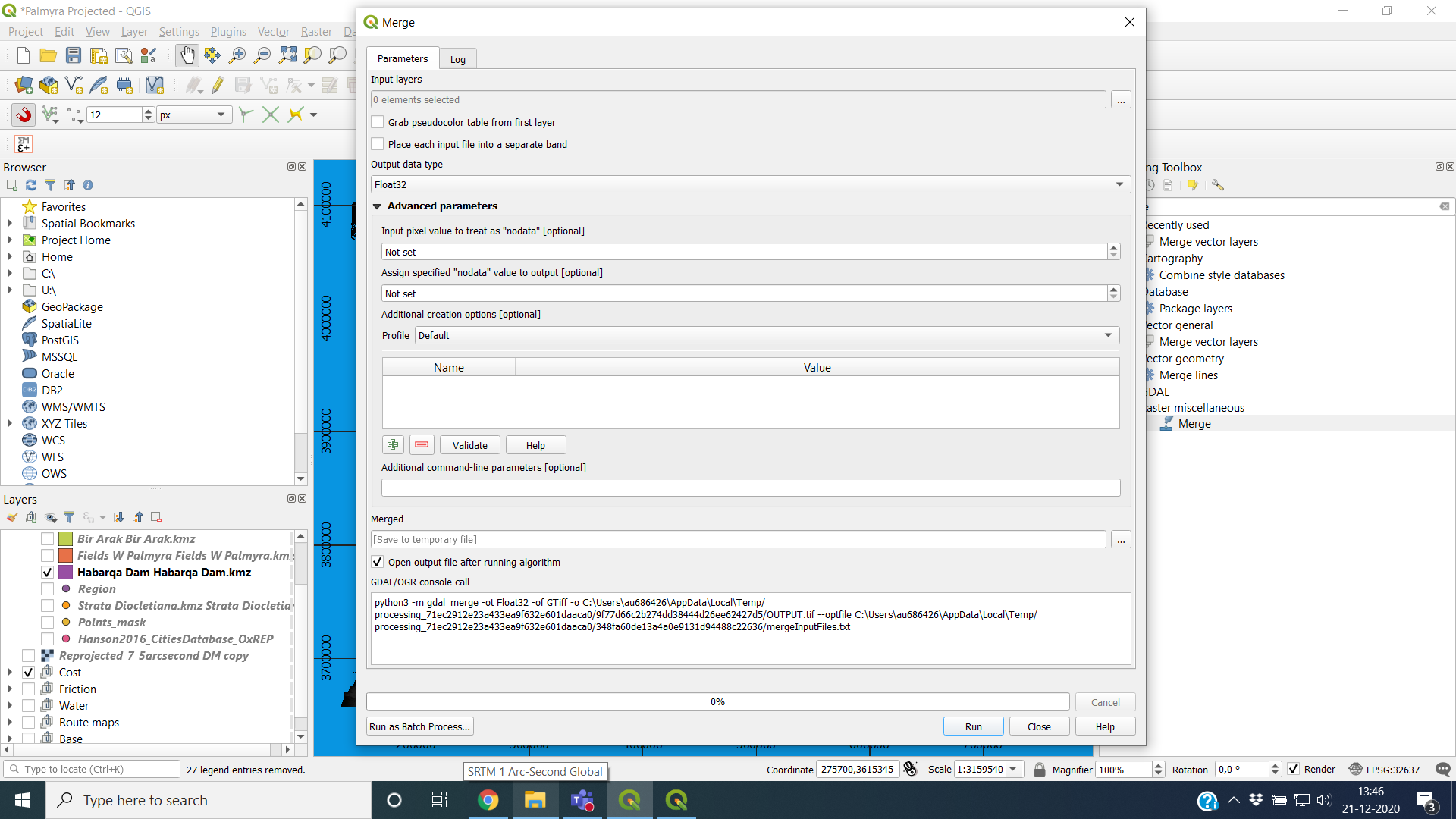
**

1. **Layer> Add layer> Add vector layer ….** Source type is “File”. Load the four .kml files in Seland (2), the water-sources in the Syrian Desert (1.0), with the cisterns, the springs, and the major and minor wells. Then, select each layer, right-click and **Export > Save features as ...** the four files.

**Process: Export> Save features as …**

**Input:** “SyrianDesert(Water Source Type)”

**Parameters:**

- **Format:** ESRI Shapefile
- **File name:** (Keep the same name for each file, which are “SyrianDesertCisternPoolReservoir”, “SyrianDesertMajorWells”, “SyrianDesertMinorWells”, and “SyrianDesertSprings”, respectively)
- **Layer name:** Leave Blank
- **CRS:** Project CRS: EPSG:32637 - WGS 84/ UTM zone 37N
- **Encoding:** UTF-8
- **Select fields to export and their export options:** Select All
- **Geometry type:** Automatic
- **Extent:** Leave unchecked, as (current: layer)
- **Layer Options:** RESIZE = NO, SHPT = Leave blank
- **Custom options:** Leave blank

**Output:** 4 point shapefiles, reprojected to the correct Coordinate System.

Erase the original .kml layers


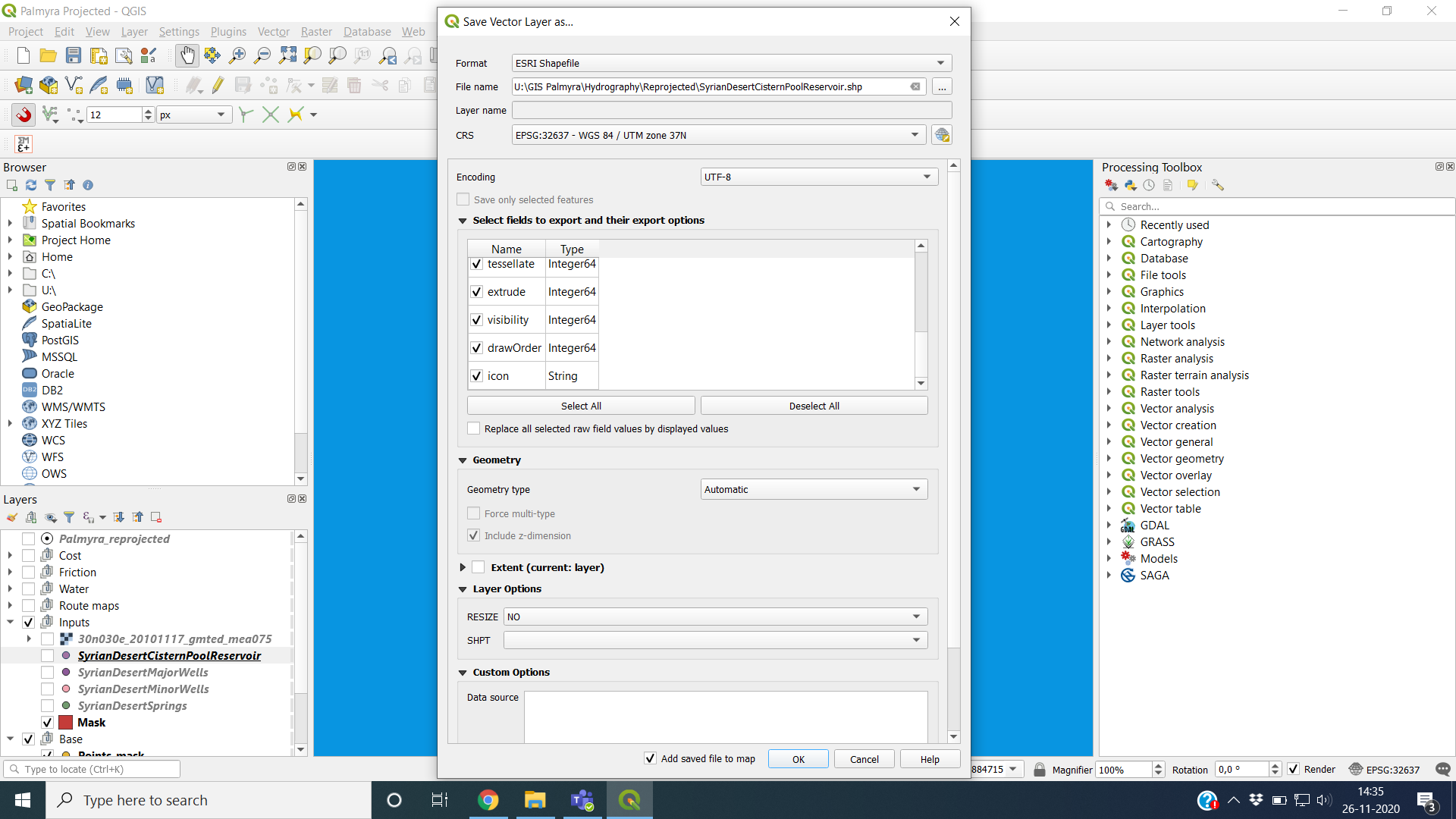


1. Go to OXREP´s Roman cities database (3,4) DOI: <<https://doi.org/10.5287/bodleian:eqapevAn8>>. Download the “Cities” table in .csv format.
2. Load “Hanson2016_Cities_OxREP.csv” as a delimited text layer in QGis. Go to **Layer> Add layer> Add delimited text layer ...**

**Input:** Hanson2016_Cities_OxREP.csv

**Parameters:**

- **Layer Name:** Hanson2016_Cities_OxREP
- **Encoding:** UTF-8
- **File Format:** CSV
- **Record and Fields Options:**
  - Number of Header lines to discard = 0
  - Tick “First record has field names”
  - Tick “Detect field types”
- **Geometry Definitions:**
  - Tick “Point coordinates”
  - X field = Longitude (X)
  - Y field = Latitude (Y)
- **Geometry CRS** = Default CRS: EPSG:4326 - WGS 84
- **All other parameters are left blank**

**Output:** A new point layer, “Hanson2016_Cities_OxREP”, is added to the project.


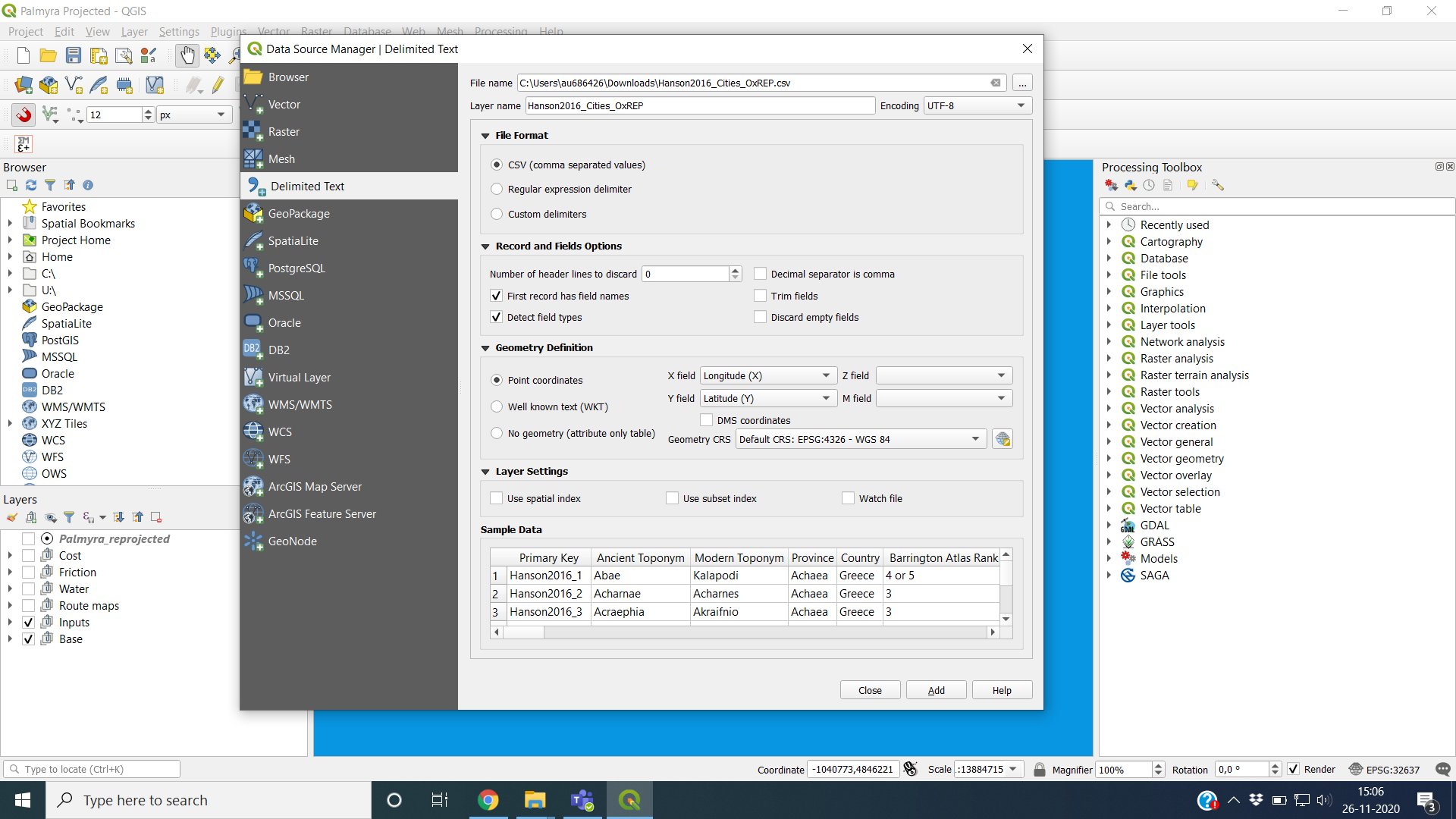


1. Select the “Hanson2016_Cities_OxREP” layer, and then select the point for Palmyra. Right click the layer and go to **Export> Save selected features as ...**

**
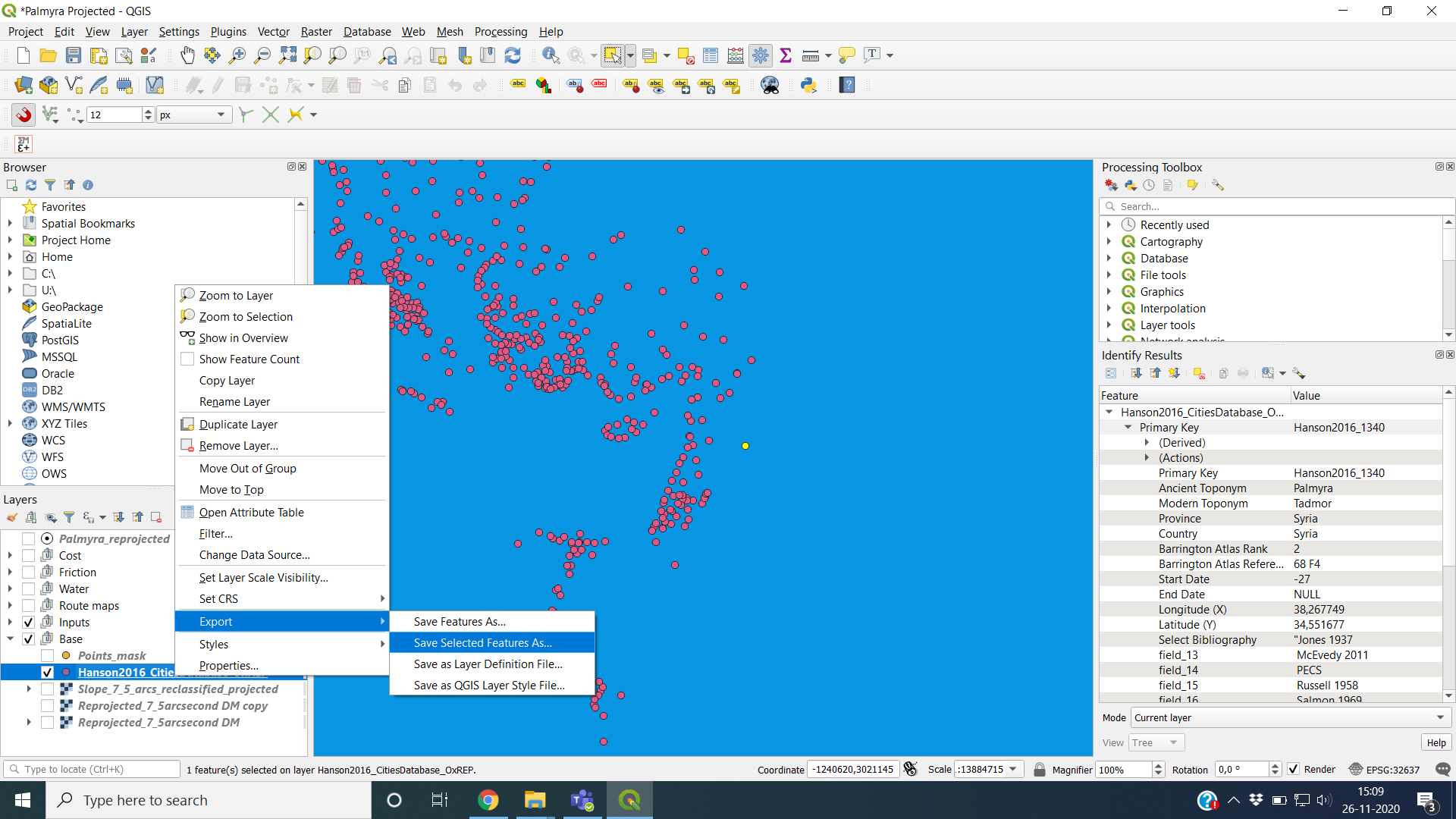
**

**Process: Export> Save selected features as …**

**Input:** “Hanson2016_Cities_OxREP”

**Parameters:**

- **Format:** ESRI Shapefile
- **File name:** Palmyra_Reprojected.shp
- **Layer name:** Leave Blank
- **CRS:** Project CRS: EPSG:32637 - WGS 84/ UTM zone 37N
- **Encoding:** UTF-8
- **Save only selected features:** Yes
- **Select fields to export and their export options:** Select All
- **Geometry type:** Automatic
- **Extent:** Leave unchecked, as (current: layer)
- **Layer Options:** RESIZE = NO, SHPT = Leave blank
- **Custom options:** Leave blank

**Output:** Palmyra_Reprojected.shp.

Add the new shapefile to the project.


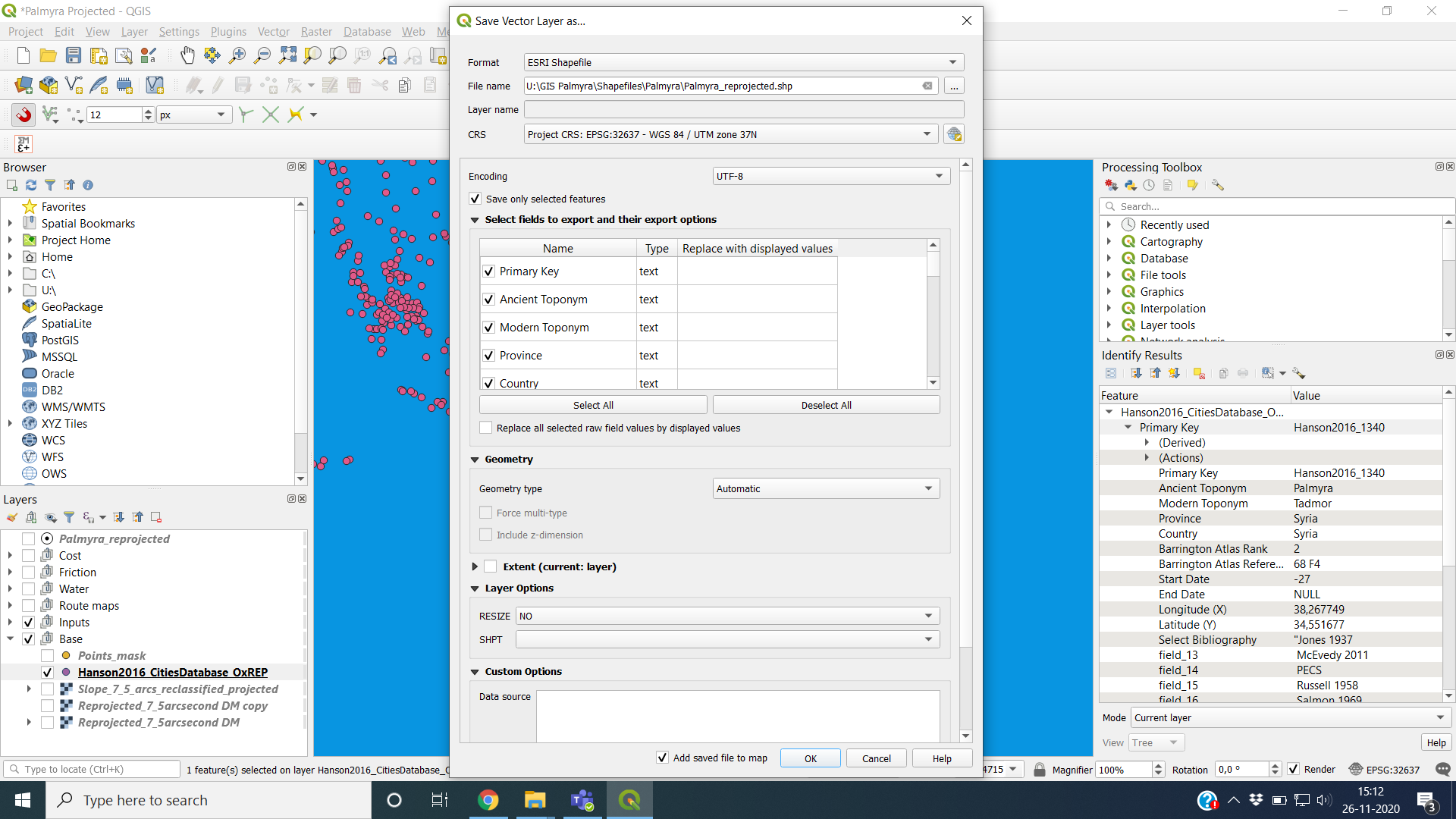


1. Georeference the Levant 1 / 500 000 : provisional "Goings" overprint map of Syria, (5).

**Process: Raster> Georeferencer**

**Inputs: The 4 sheets (1, 2, 4 and 5) of the map surrounding Palmyra**

**Parameters:**

- **Transformation type:** Thin Plate Spline
- **Resampling method:** Linear
- **Target SRS:** ESRI:104140 - GCS_Voirol_1879_Grad
- **Compression:** None
- **Save GCP points:** Yes
- **Use 0 for transparency when needed:** No
- **Set target resolution:** No
- **Generate PDF map:** Leave blank
- **Generate PDF report:** Leave blank

**Output:** British_Map_1.tif; British_Map_2.tif; British_Map_4.tif; British_Map_5.tif

**
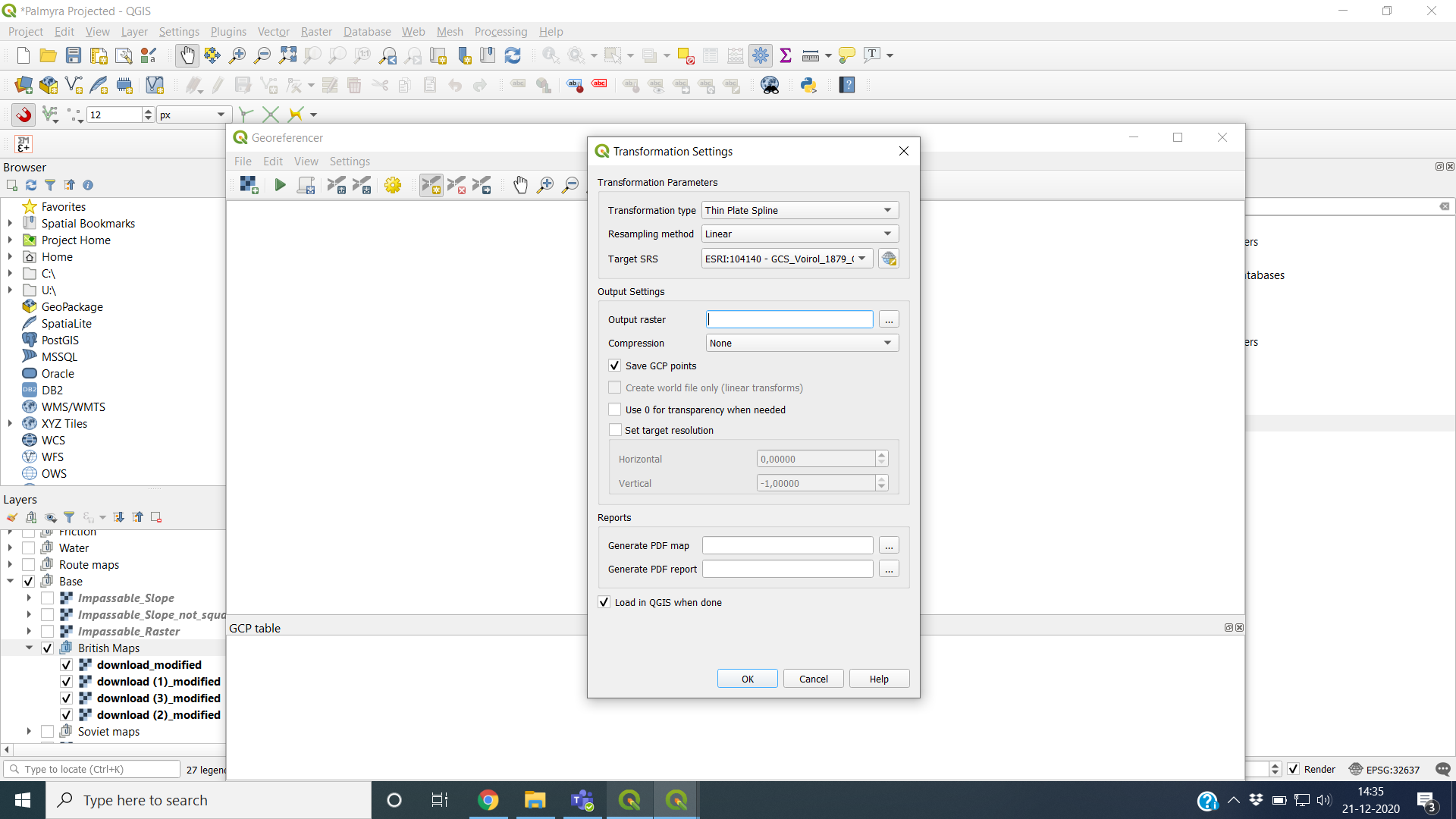
**

1. Generate a new vector layer with the Impassable areas

**Process: Layer> Create layer> New shapefile layer…**

**Input:** -

**Parameters:**

- **File name:** Impassable.shp
- **File Encoding:** UTF-8
- **Geometry Type:** Polygon
- **Additional dimensions:** None
- **CRS:** Project CRS: EPSG:32637 - WGS 84/ UTM zone 37N
- **New Field:** Flat_Increase.
- **Fields List:** Name=id, Type=Integer, Length=10, Precision= (blank)

Name=Flat_Increase, Type=Float, Length=10, Precision=3

**Output:** Impassable.shp

1. Select the “Impassable.shp” Layer. Toggle “**Toggle editing”** and select “**Add Polygon Feature”**. Manually generate polygons for each of the blue “Impassable” areas in the British maps in a rough 100-130 km radius of Palmyra. When done, right click and assign a sequential number (1, 2, 3 …) to each polygon to the “id” field. Assign the value 50 to the Flat_Increase field. Select **“Save Layer Edits”** and then **“Toggle editing”** again.

***Generating the Slope-Based Friction Surface***

1. **Process: Warp (Reproject) (Processing Toolbox> GDAL> Raster Projections> Warp (Reproject))**

**Inputs:** the Raster “Combined_DEM_Syria.tif”

**Parameters:**

- **Source CRS:** EPSG:4326 - WGS 84
- **Target CRS:** EPSG:32637 - WGS 84/UTM zone 31N
- **Resampling method to use:** Nearest Neighbour
- **NoData value for output bands:** Not set
- **Output file resolution in target georeferenced units:** 30
- **Additional creation options:** Leave blank
- **Output data type:** Use Input Layer Data type
- **Georeferenced extent of output file to be created:** Leave blank
- **Use Project CRS =** Yes
- **Use Multithreaded warping implementation =** No
- **Additional command-line parameters:** Leave blank

**Output:** Reprojected_1arcsecond_DM.tif

**
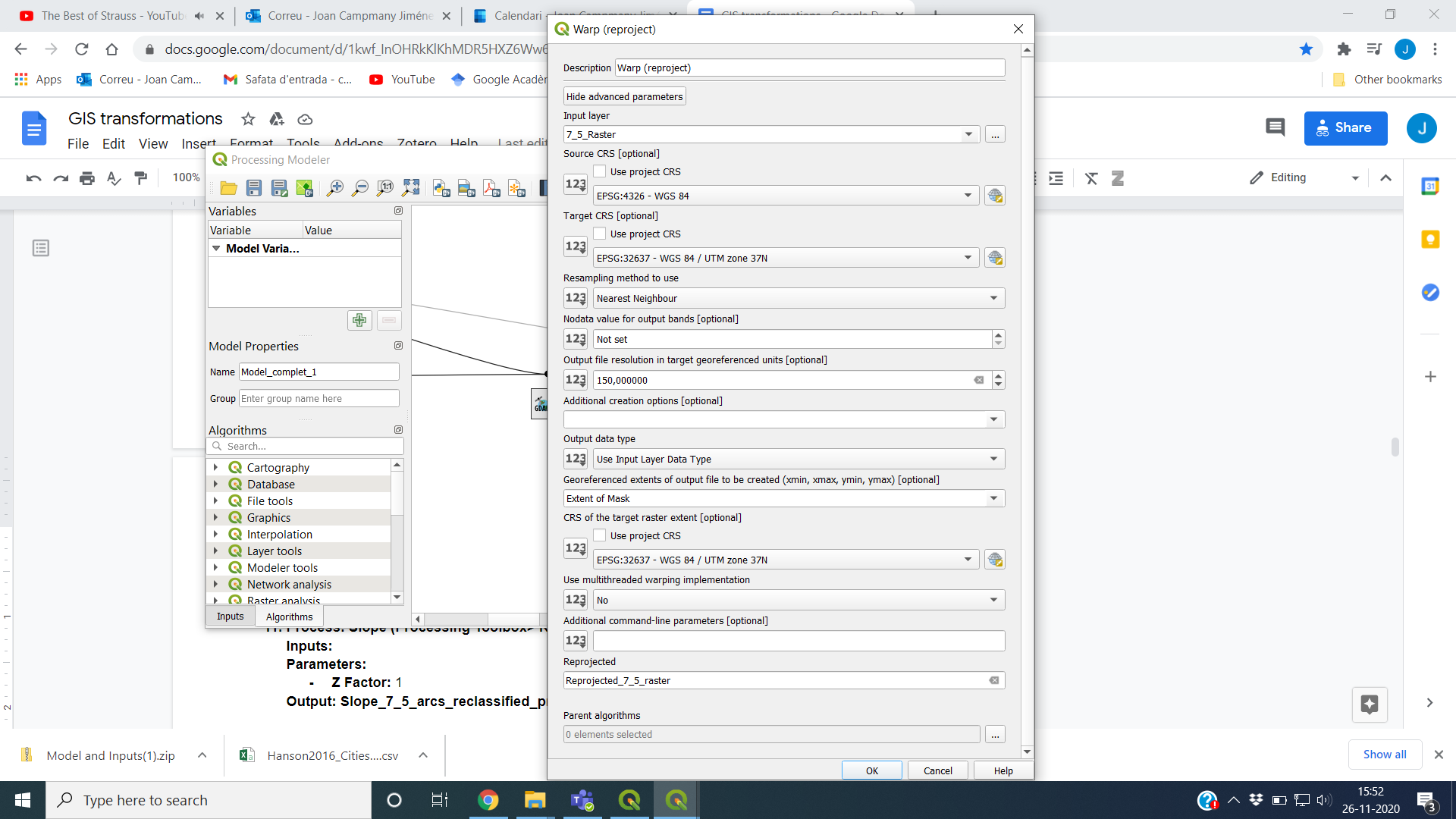
**

1. **Process: Slope** **(Processing Toolbox> Raster Terrain Analysis> Slope)**

**Inputs:** Reprojected_1arcsecond_DM.tif

**Parameters:**

- **Z Factor:** 1

**Output:** Slope_1_arcs_reclassified_projected.tif


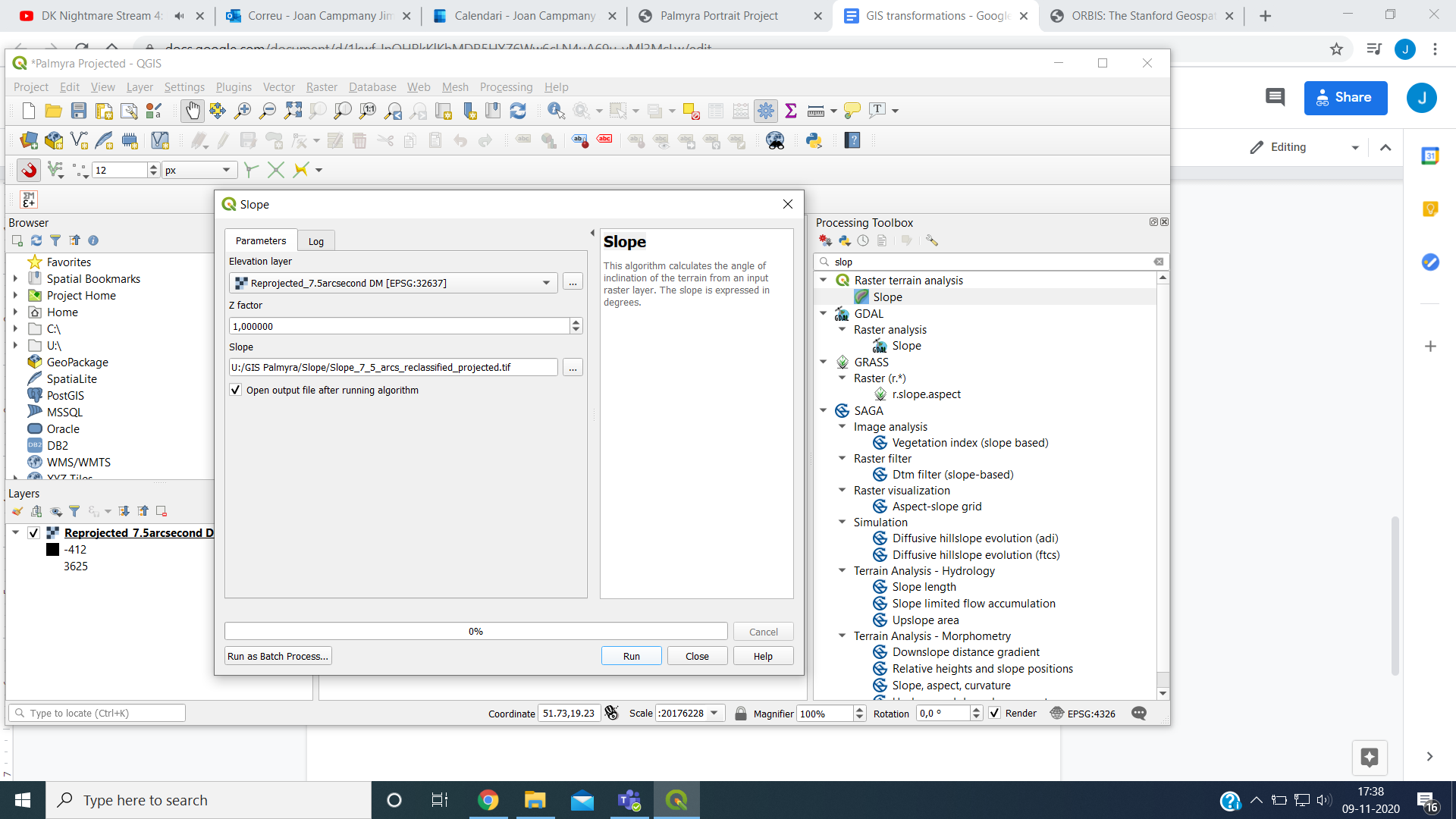


1. To modify the slope raster before its transformation into a Friction Raster, we will need to rasterize the Impassable areas.

**Process: Rasterize (SAGA> Raster creation tools> Rasterize)**

**Inputs:** Impassable.shp

**Parameters:**

- **Attribute:** 2
- **Output values:** [2] attribute
- **Method for Multiple Values:** [3] maximum
- **Method for Lines:** [1] thick
- **Method for Lines:** [1] cell
- **Prefered Target Grid Type:** [3] Floating Point (4 byte)
- **Output Extent (xmin, xmax, ymin, ymax) [optional]:** Extent of Slope_1_arcs_reclassified_projected.tif
- **Cellsize:** 30,000000
- **Fit:** [1] cells

**Output:** Impassable_Areas.tif

**
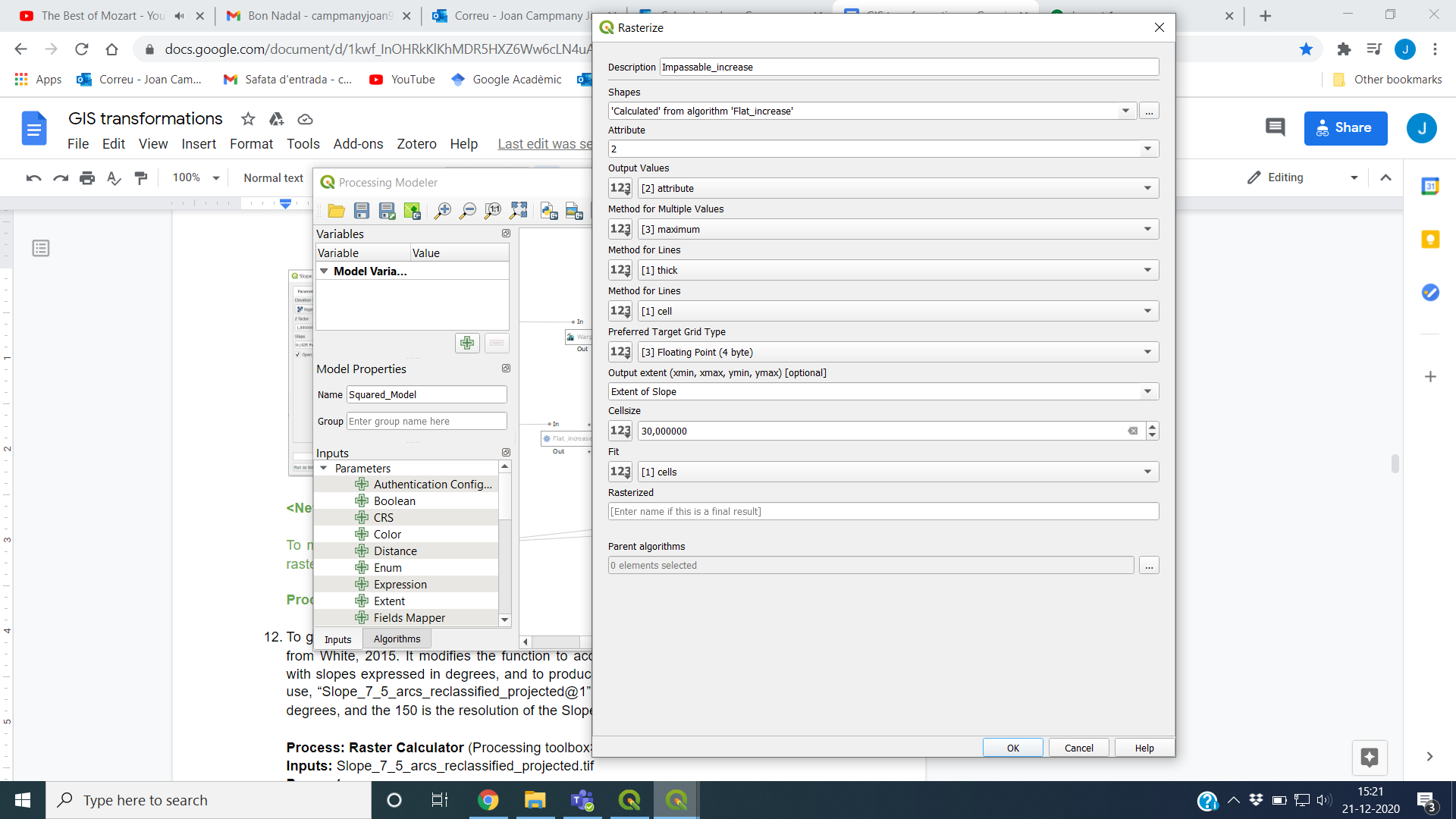
**

1. Run “r.null” **(grass7:r.null)**, and add a value (0) to Null cells.

**Process: r.null (Processing toolbox> GRASS> Raster (r.*)> r.null)**

**Input:** Impassable_Areas.tif

**Parameters:**

- **List of cell values to be set to NULL (optional):** Leave blank
- **The value to replace the null value by (optional):** 0,000000

**Output:** Impassable_Null.tif

**
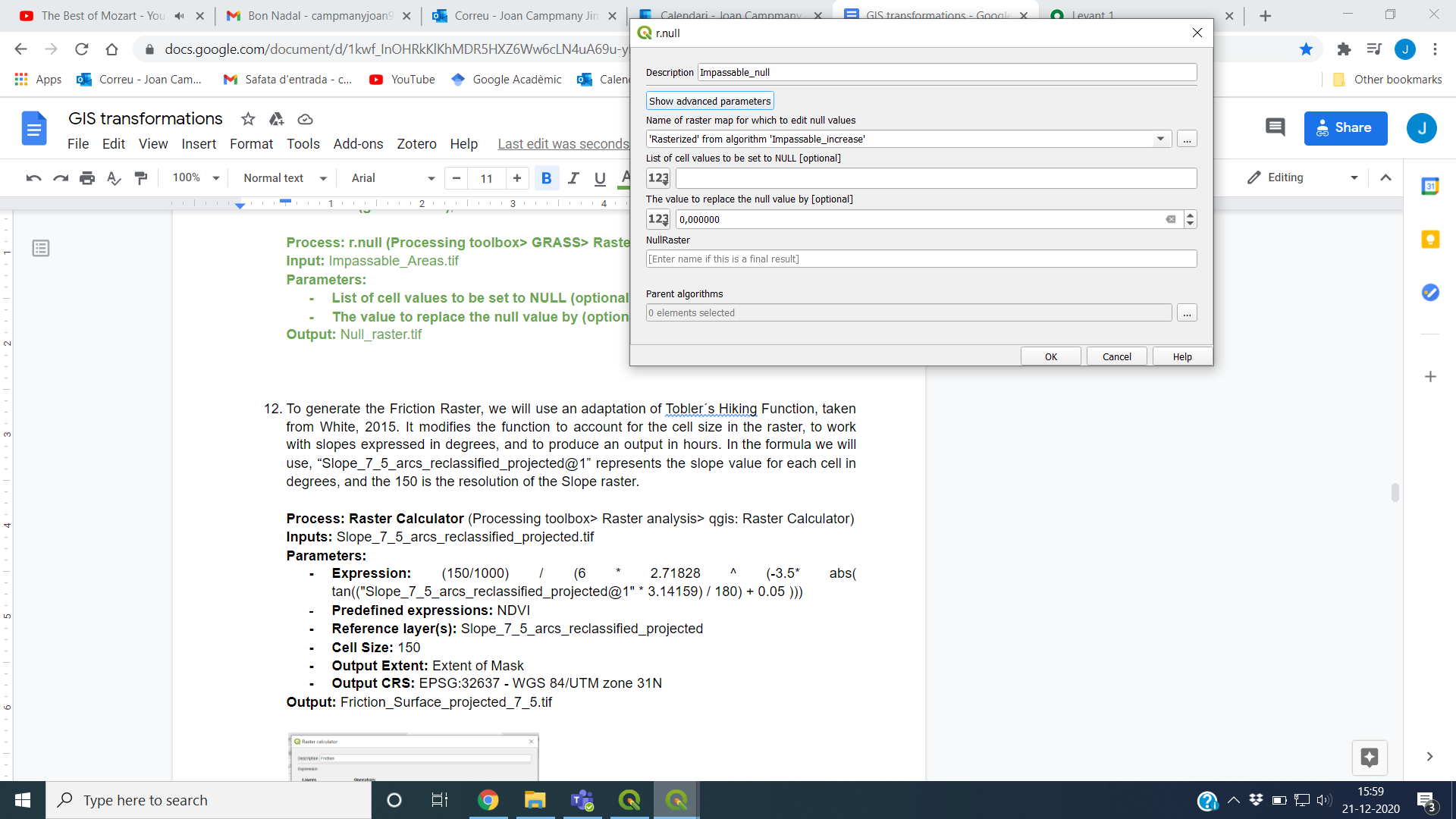
**

1. Now, we need to modify the slope raster to increase the effect of slopes on movement and to make certain areas, as taken from the British Army “Goings” map, almost impassable. To do this, we will first add 50 degrees to all slopes within the impassable areas

**Process: r.series (Processing toolbox> GRASS> Raster (r.*)> r.null)**

**Input:** Impassable_null.tif, Slope_1_arcs_reclassified_projected.tif

**Parameters:**

- **Propagate NULLs:** Yes
- **Aggregate operation (optional):** sum
- **Quantile to calculate for method=quantile (optional):** Leave blank
- **Weighting factor for each input map, default value in 1,0 (optional):** Leave blank

**Output:** Amplified_Impassable_1.tif


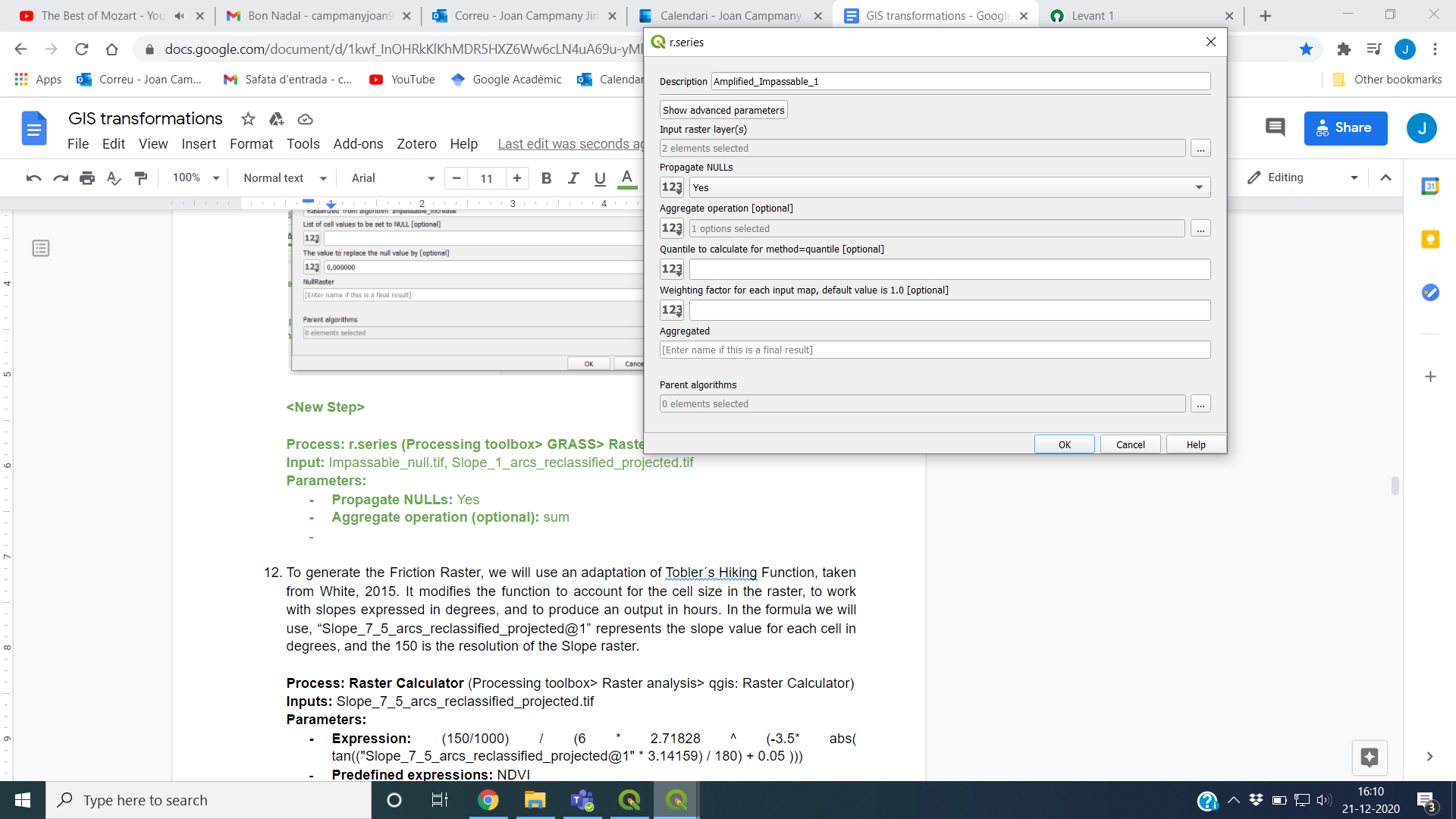


1. **Process: Raster Calculator** (Processing toolbox> Raster analysis> qgis: Raster Calculator)

**Inputs:** Amplified_Impassable_1.tif

**Parameters:**

- **Expression:** ”Amplified_Impassable_1@1”^2
- **Predefined expressions:** NDVI
- **Reference layer(s):** Reprojected_1arcsecond_DM.tif
- **Cell Size:** 30
- **Output Extent:** Extent of Reprojected_1arcsecond_DM.tif
- **Output CRS:** EPSG:32637 - WGS 84/UTM zone 31N

**Output:** “Amplified_Impassable_2.tif

1. To generate the Friction Raster, we will use an adaptation of Tobler´s Hiking Function (6), taken from White (7). Our output will not be in hours as in the original function, but in flat areas close to water sources the difference will be minimal. In the formula we will use, “Amplified_Impassable_2@1” represents the slope value for each cell in degrees, and the 30 is the resolution of the modified Slope raster.

**Process: Raster Calculator** (Processing toolbox> Raster analysis> qgis: Raster Calculator)

**Inputs:** Amplified_Impassable_2.tif

**Parameters:**

- **Expression:** (30/1000) / (6 * 2.71828 ^ (-3.5* abs( tan(("Amplified_Impassable_2@1" * 3.14159) / 180) + 0.05 )))
- **Predefined expressions:** NDVI
- **Reference layer(s):** Amplified_Impassable_2.tif
- **Cell Size:** 30
- **Output Extent:** Extent of Amplified_Impassable_2.tif
- **Output CRS:** EPSG:32637 - WGS 84/UTM zone 31N

**Output:** Friction_Impassable.tif


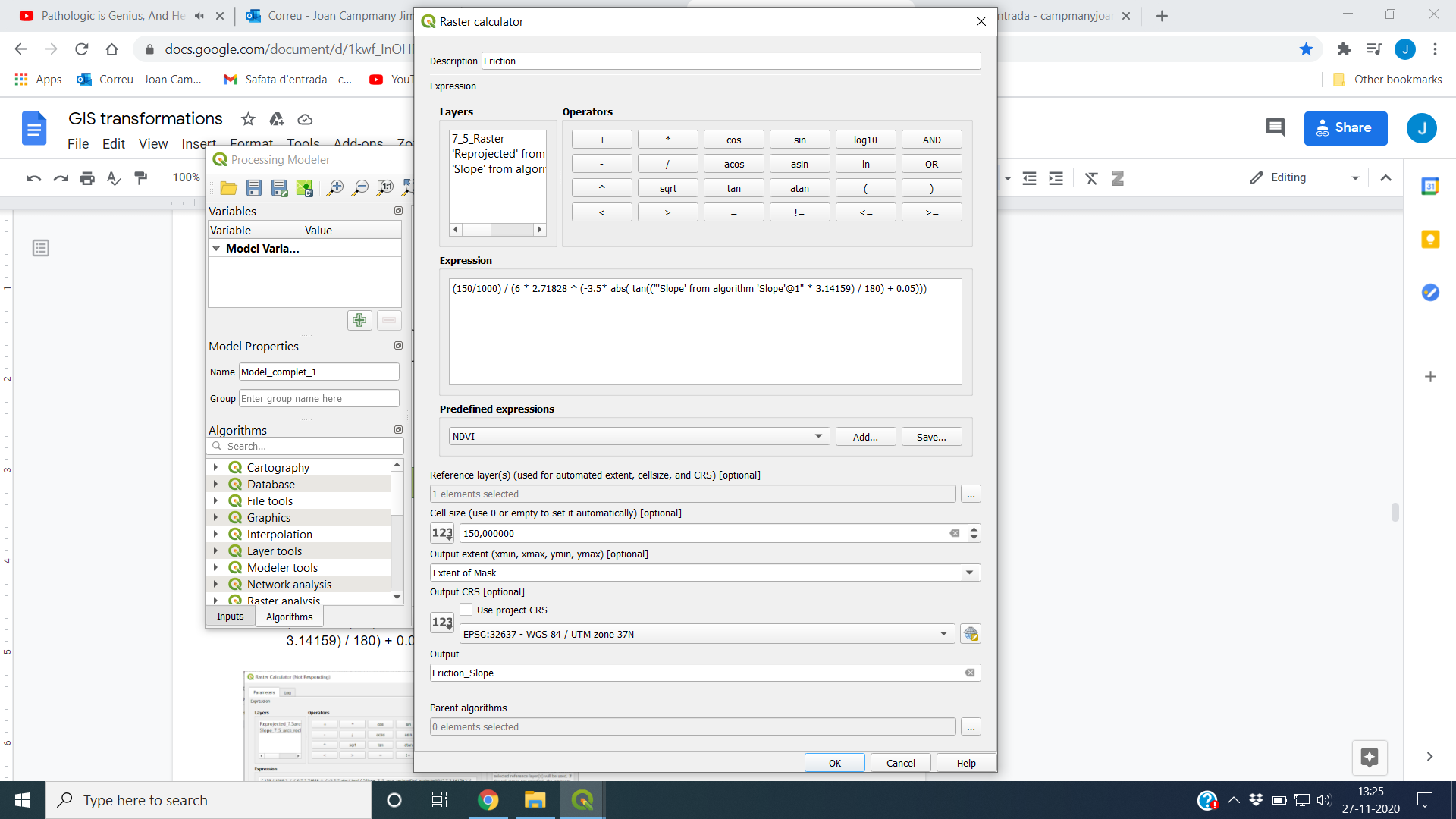


***Slope-based accumulated cost raster***

**Process: r.cost (Processing Toolbox> GRASS> Raster (r.*)> r.cost)**

**Inputs:** Friction_Impassable.tif, Palmyra_reprojected.shp

**Parameters:**

- **Coordinates of Starting point(s), (E, N)(optional):** Leave blank
- **Coordinates of Stoping point(s), (E, N)(optional):** Leave blank
- **Use the “Knight’s move”, slower, but more accurate:** Yes
- **Keep null values in output raster layer:** Yes
- **Start Points (optional):** Palmyra_reprojected
- **Stop Points (optional):** Leave blank
- **Name of starting raster points map (optional):** [Not set]
- **Maximum cumulative Cost:** Leave blank
- **Cost assigned to Null cells. By defaut, Null cells are excluded (optional):** Not set
- **Maximum memory to be used in MB:** 300

**Outputs:** Cost_Impassable.tif **(Cumulative cost)** , Cost_Impassable_Backlink.tif **(Movement Directions). The cost allocation map is not necessary, and can be omitted.**


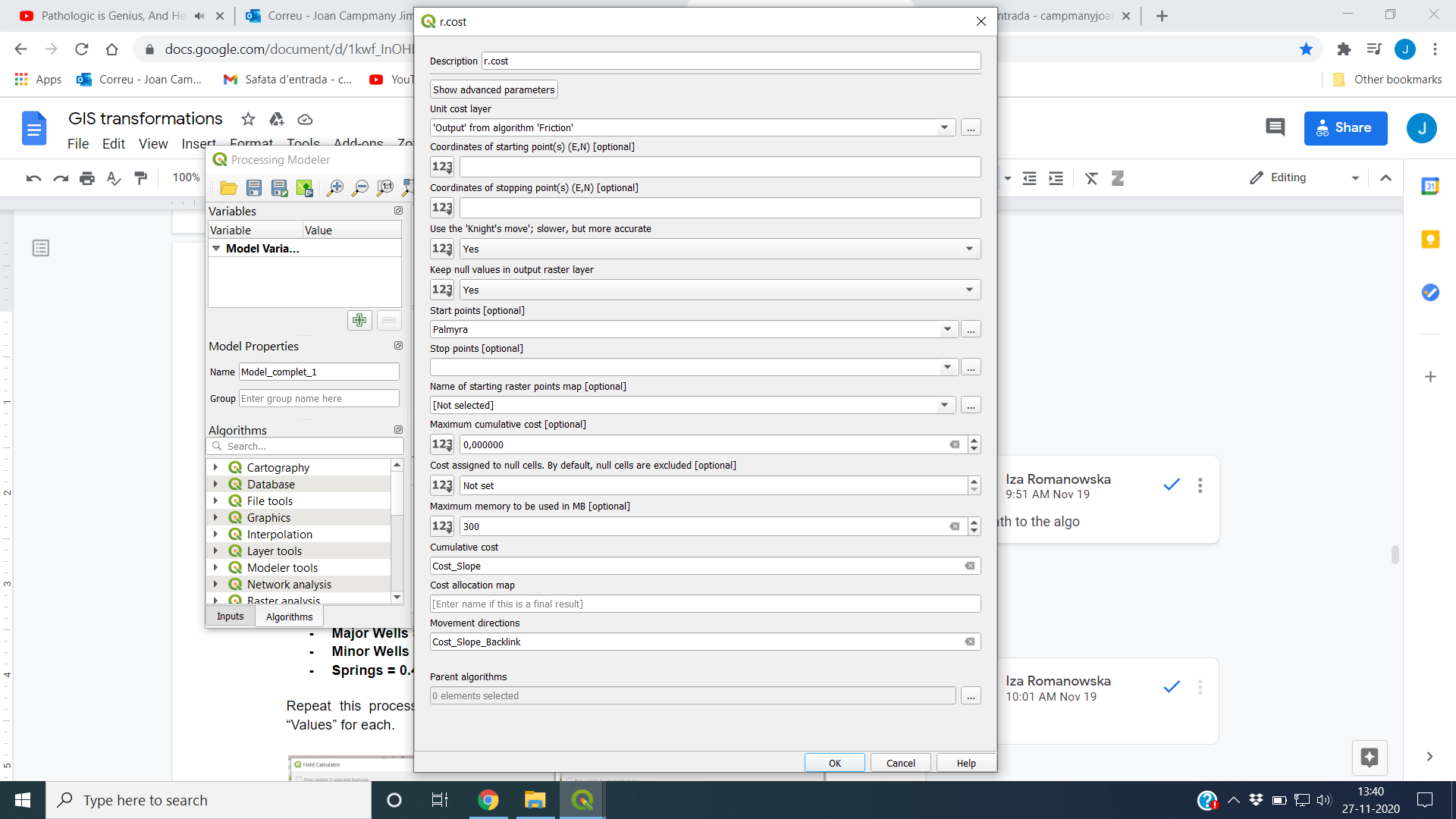


***Hydrology-based friction surface***

1. **The following process needs to be repeated once for each of the water source shapefiles, a total of 4 times.**

**Process: Field Calculator (Processing Toolbox> Vector table> Field Calculator)**

**Inputs:** SyrianDesertCisternPoolReservoir.shp, SyrianDesertMajorWells.shp, SyrianDesertMinorWells.shp, and SyrianDesertSprings.shp

**Parameters:**

- **Result field name:** Type
- **Field type:** String
- **Field length:** 10
- **Field precision:** 3
- **Create new field =** Yes
- **Formula (or Expression):** ‘(Write here the type of water source, for example Cistern or Major Well)’

**Output:** A new attribute is added to the table, “Type”.

**
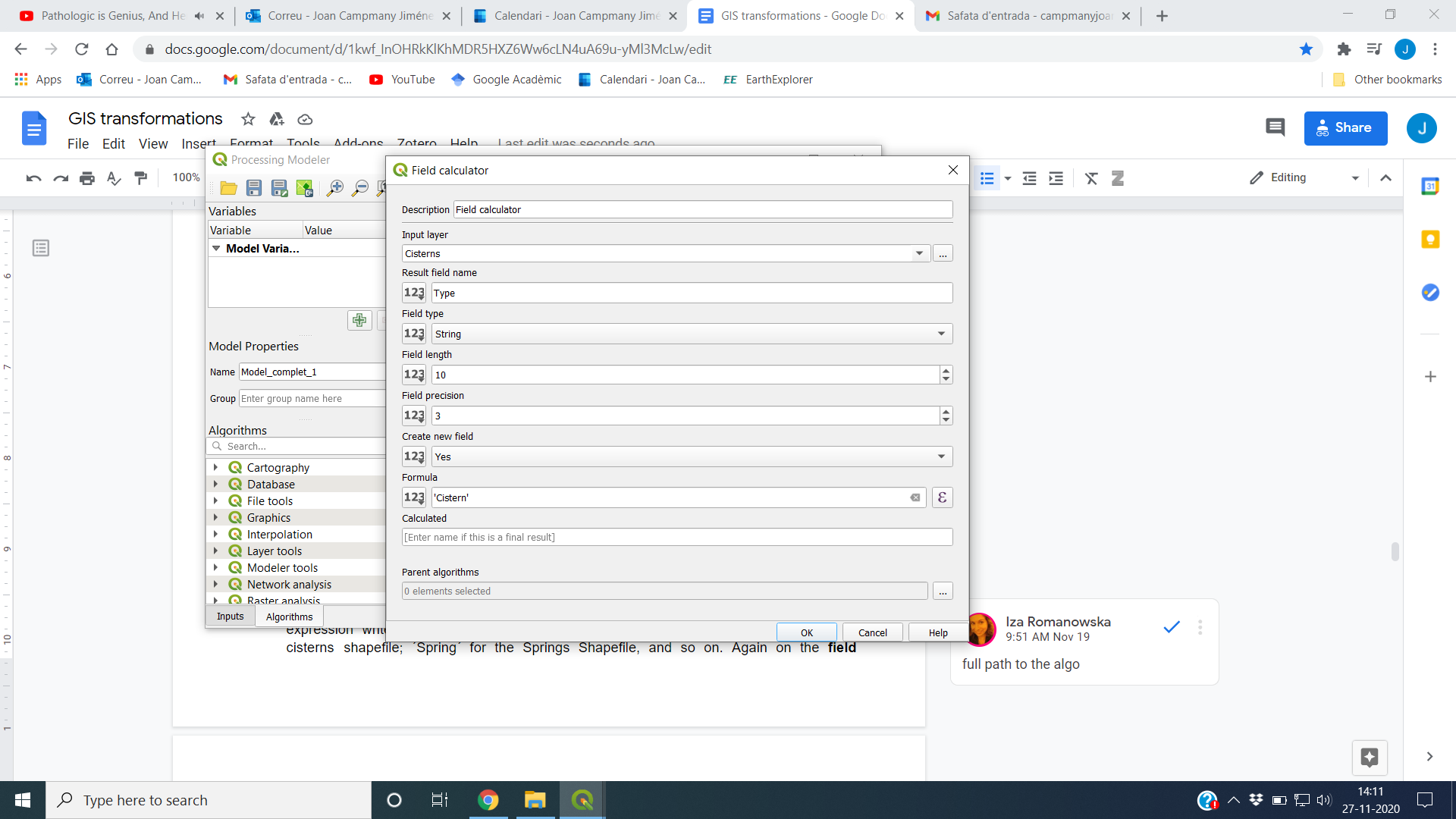
**
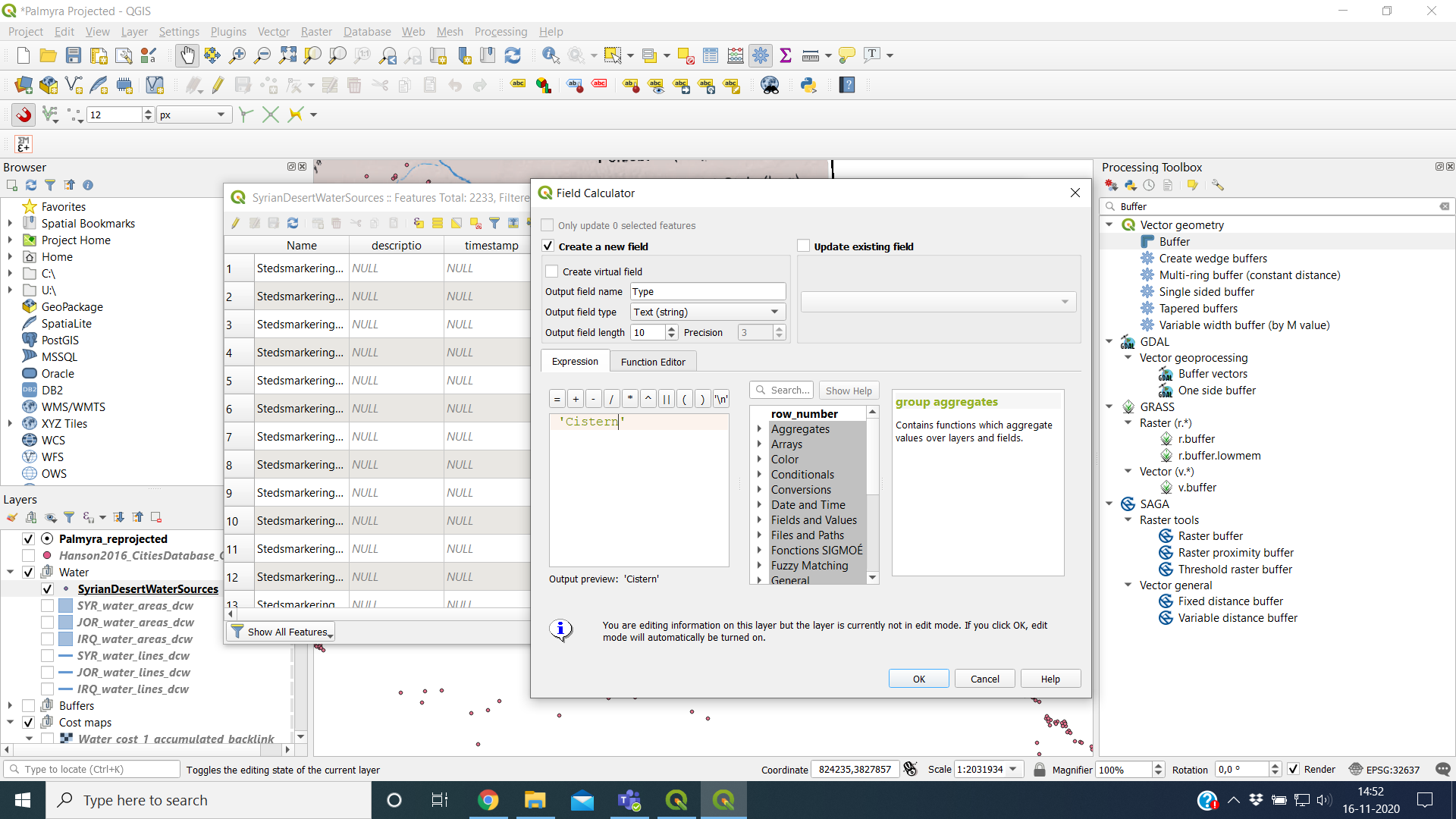


**Process: Field Calculator (Processing Toolbox> Vector table> Field Calculator)**

**Inputs:** SyrianDesertCisternPoolReservoir.shp, SyrianDesertMajorWells.shp, SyrianDesertMinorWells.shp, and SyrianDesertSprings.shp

**Parameters:**

- **Result field name:** Reliable
- **Field type:** Decimal Number (real)
- **Field length:** 10
- **Field precision:** 3
- **Create new field =** Yes
- **Formula (or Expression):** ‘(Write here the value assigned to each source type, like 1 or 0.8)’

**Output:** A new attribute is added to the table, “Reliable”.


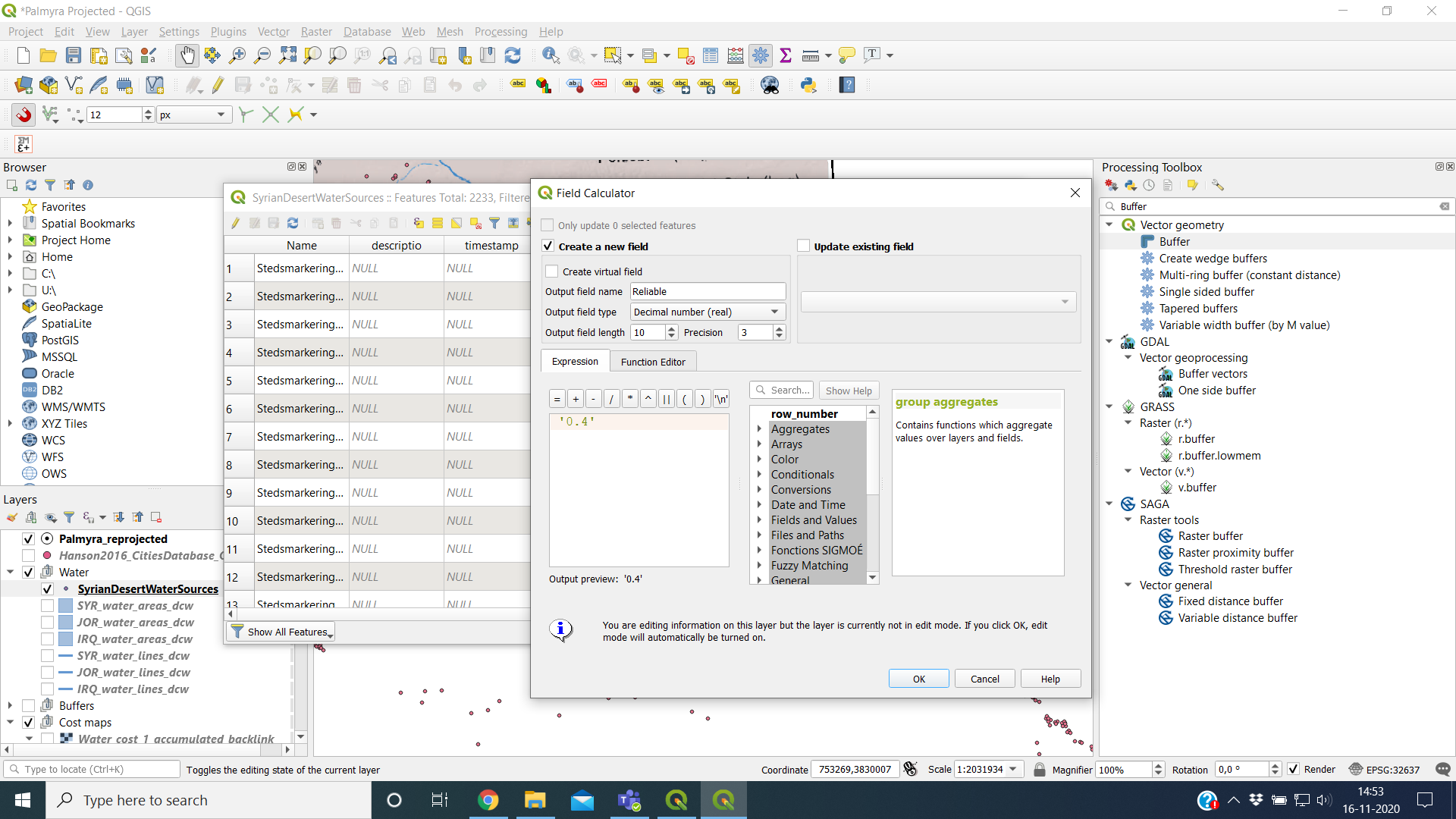


| ***File Name*** | ***Value for “Type”*** | ***Provisional value for “Reliable”*** |
| --- | --- | --- |
| SyrianDesertCisternPoolReservoir.shp | ‘Cistern’ | ‘0.7’ |
| SyrianDesertMajorWells.shp | ‘Major Well’ | ‘0.9’ |
| SyrianDesertMinorWells.shp | ‘Minor Well’ | ‘0.8’ |
| SyrianDesertSprings.shp | ‘Spring’ | ‘1’ |

1. **Merge** all of the water-source shapefiles.

**Process: Merge vectors layers (Processing toolbox> Vector general> Merge vector layers)**

**Inputs:** SyrianDesertCisternPoolReservoir.shp, SyrianDesertMajorWells.shp, SyrianDesertMinorWells.shp, SyrianDesertSprings.shp

**Parameters:**

- **Destination CRS:** EPSG:32637 - WGS 84/UTM zone 31N

**Output:** SyrianDesertWaterSources.shp


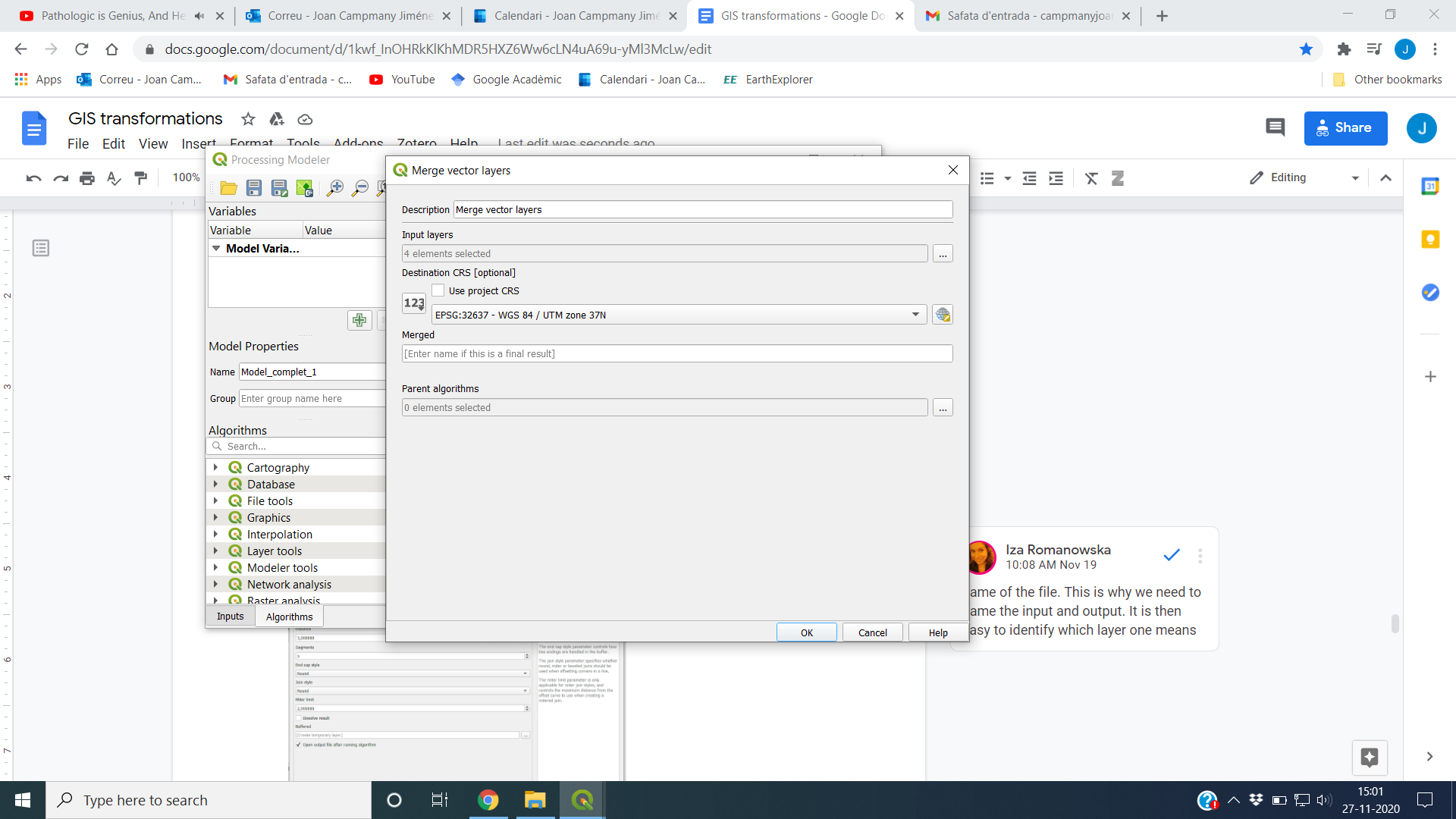


1. Create a **Buffer (native:buffer)** for the water source dots, at 10 km.

**Process: Buffer (Processing toolbox> Vector geometry> Buffer)**

**Input:** SyrianDesertWaterSources.shp

**Parameters:**

- **Distance:** 10km
- **Segments:** 5
- **End cap style:** Round
- **Join style:** Round
- **Miter limit:** 2
- **Dissolve result:** No

**Output:** 10kmbuffer.shp


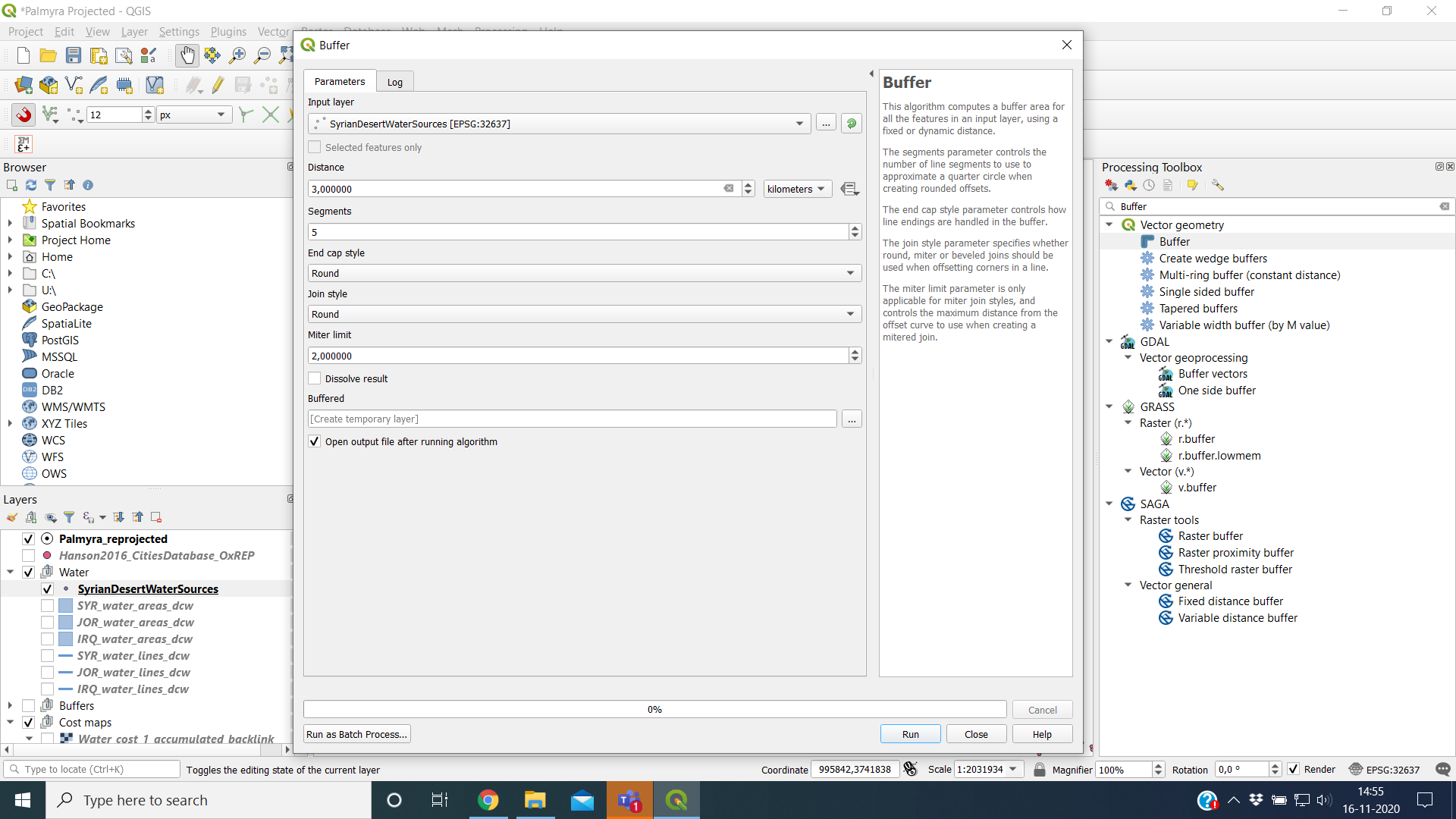


1. **Rasterize (saga:rasterize)** the buffer layer.

**Process: Rasterize (SAGA> Raster creation tools> Rasterize)**

**Input:** 10kmbuffer.shp

**Parameters:**

- **Attribute:** Reliable
- **Output Values:** [2] attribute
- **Method for Multiple Values:** [3] maximum
- **Method for Lines:** [1] thick
- **Method for Lines:** [1] cell
- **Preferred Taget Grid Type:** [3] Floating Point (4 byte)
- **Output extent:** Extent of Reprojected_1arcsecond_DM.tif
- **Cellsize:** 30
- **Fit:** [1] cells

**Output:** Buffer_rasterized.tif


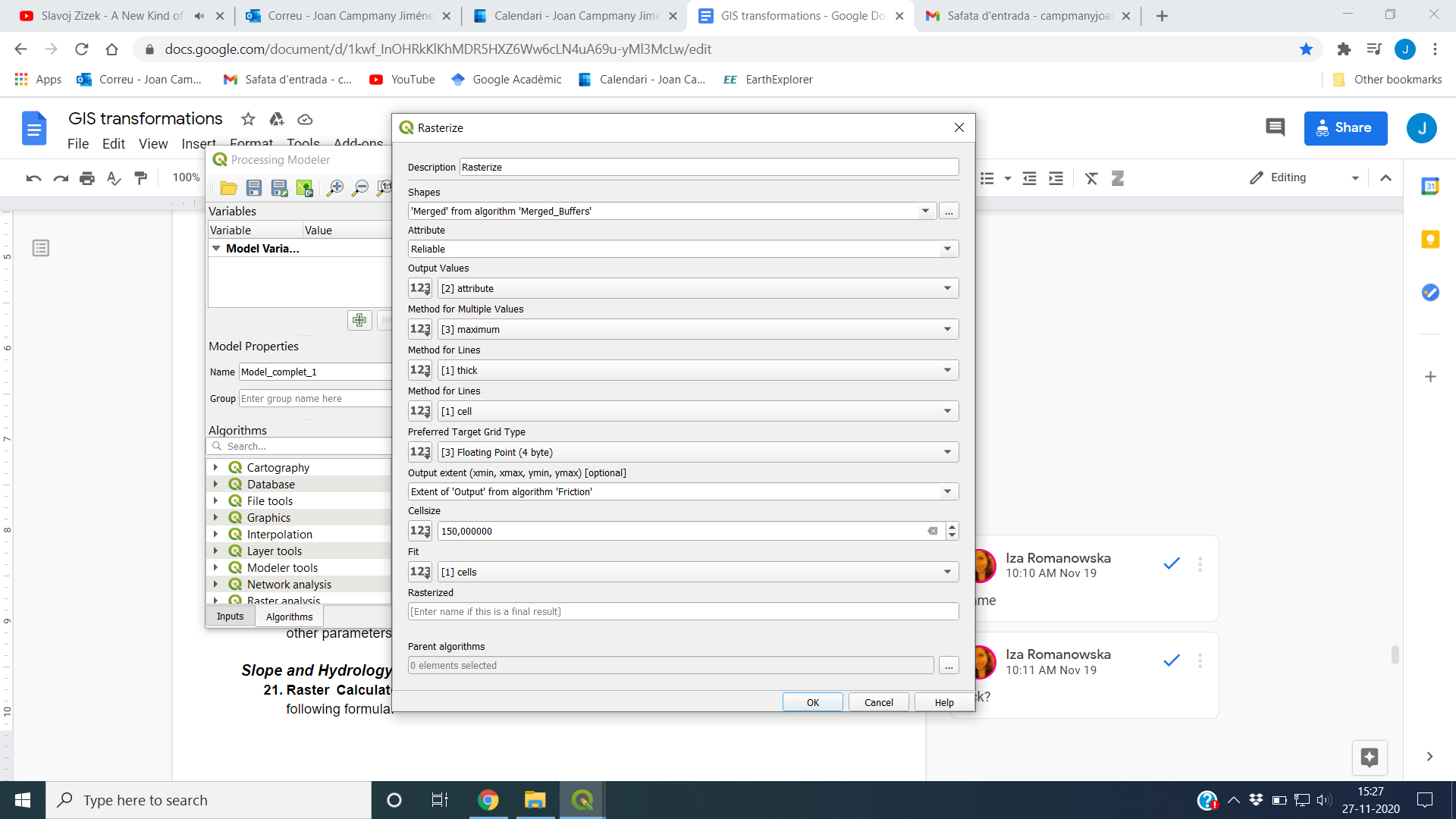


1. **r.null**> Run “r.null” **(grass7:r.null)**, and add a value to Null cells.

**Process: r.null (Processing toolbox> GRASS> Raster (r.*)> r.null)**

**Input:** Buffer_rasterized.tif

**Parameters:**

- **List of cell values to be set to NULL (optional):** Leave blank
- **The value to replace the null value by (optional):** 0,5

**Output:** Null_raster.tif


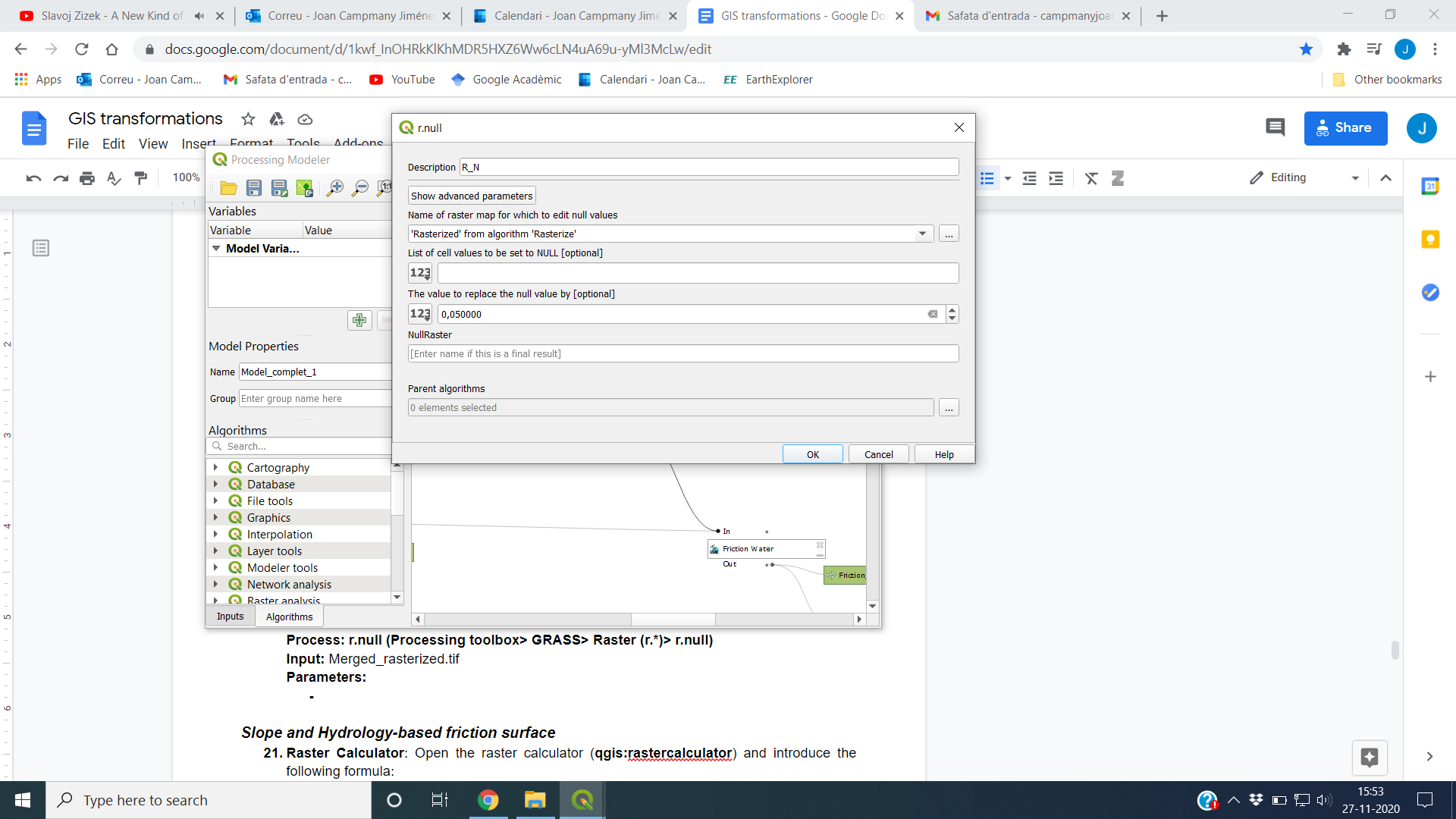


***Slope and Hidrology-based friction surface***

1. **Process: Raster Calculator (Processing Toolbox> GDAL> Raster miscellaneous> Raster calculator)**

**Inputs:** Friction_Impassable.tif, Null_raster.tif

**Parameters:**

- **Input layer A:** Friction_Impassable.tif
- **Number of raster band for A:** 1
- **Input layer B:** Null_raster.tif
- **Number of raster band for B:** 1
- **Input layer C to F:** [Not selected]
- **Number of raster band for C to F:** [Not set]
- **Calculation in gdalnumeric syntax using +-/* or any numpy array functions (i.e. logical_and()):** A/B
- **Set output nodata value (optional):** Not set
- **Output raster type:** Float32

**Output:** Friction_Impassable_water.tif


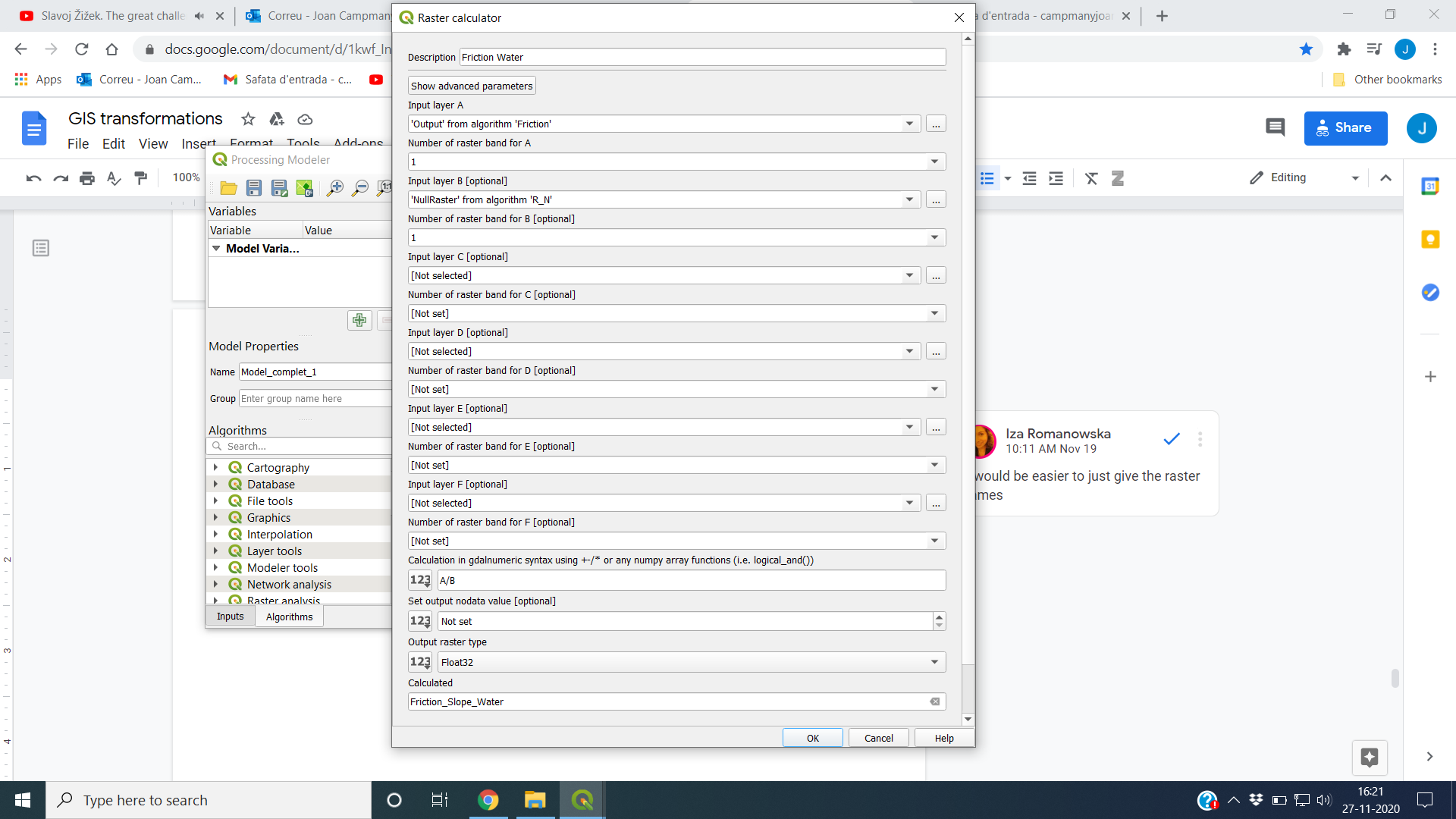


This will generate a new Friction raster incorporating both Slope and hydrology.

***Slope and Hidrology-based accumulated cost***

**Process: r.cost (Processing Toolbox> GRASS> Raster (r.*)> r.cost)**

**Input:** Friction_Impassable_water.tif

**Parameters:**

- **Coordinates of Starting point(s), (E, N)(optional):** Leave blank
- **Coordinates of Stoping point(s), (E, N)(optional):** Leave blank
- **Use the “Knight’s move”, slower, but more accurate:** Yes
- **Keep null values in output raster layer:** Yes
- **Start Points (optional):** Palmyra_reprojected
- **Stop Points (optional):** Leave blank
- **Name of starting raster points map (optional):** [Not selected]
- **Maximum cumulative Cost:** Leave blank
- **Cost assigned to Null cells. By defaut, Null cells are excluded (optional):** Not set
- **Maximum memory to be used in MB:** 300

**Outputs:** Water_Cost_3.tif **(Cumulative cost)** , Water_Cost_3_backlink.tif **(Movement Directions). The cost allocation map is not necessary, and can be omitted.**

**
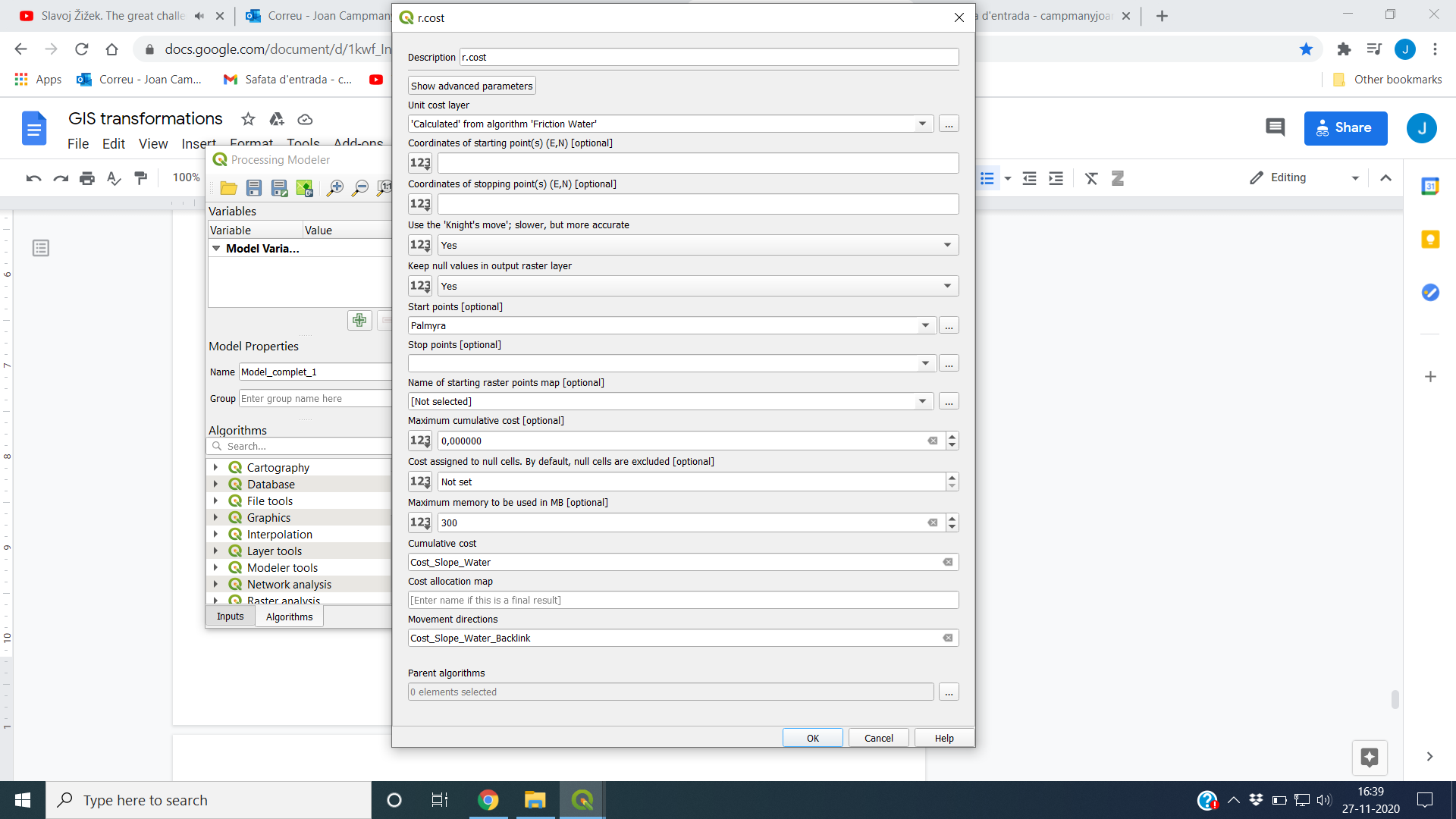
**

***Catchment areas***

1. We need to get contours for the different catchment areas surrounding palmyra. Since our Raster has too much resolution to do this practically, we will first have to lower it.

**Process: Warp (Reproject) (Processing Toolbox> GDAL> Raster Projections> Warp (Reproject))**

**Inputs:** Water_Cost_3_accumulated.tif

**Parameters:**

- **Source CRS:** EPSG:4326 - WGS 84/UTM zone 31N
- **Target CRS:** EPSG:32637 - WGS 84/UTM zone 31N
- **Resampling method to use:** Nearest Neighbour
- **NoData value for output bands:** Not set
- **Output file resolution in target georeferenced units:** 900
- **Additional creation options:** Leave blank
- **Output data type:** Use Input Layer Data type
- **Georeferenced extent of output file to be created:** Leave blank
- **Use Project CRS =** Yes
- **Use Multithreaded warping implementation =** No
- **Additional command-line parameters:** Leave blank

**Output:** Water_Cost_3_Reclassified_900.tif

**
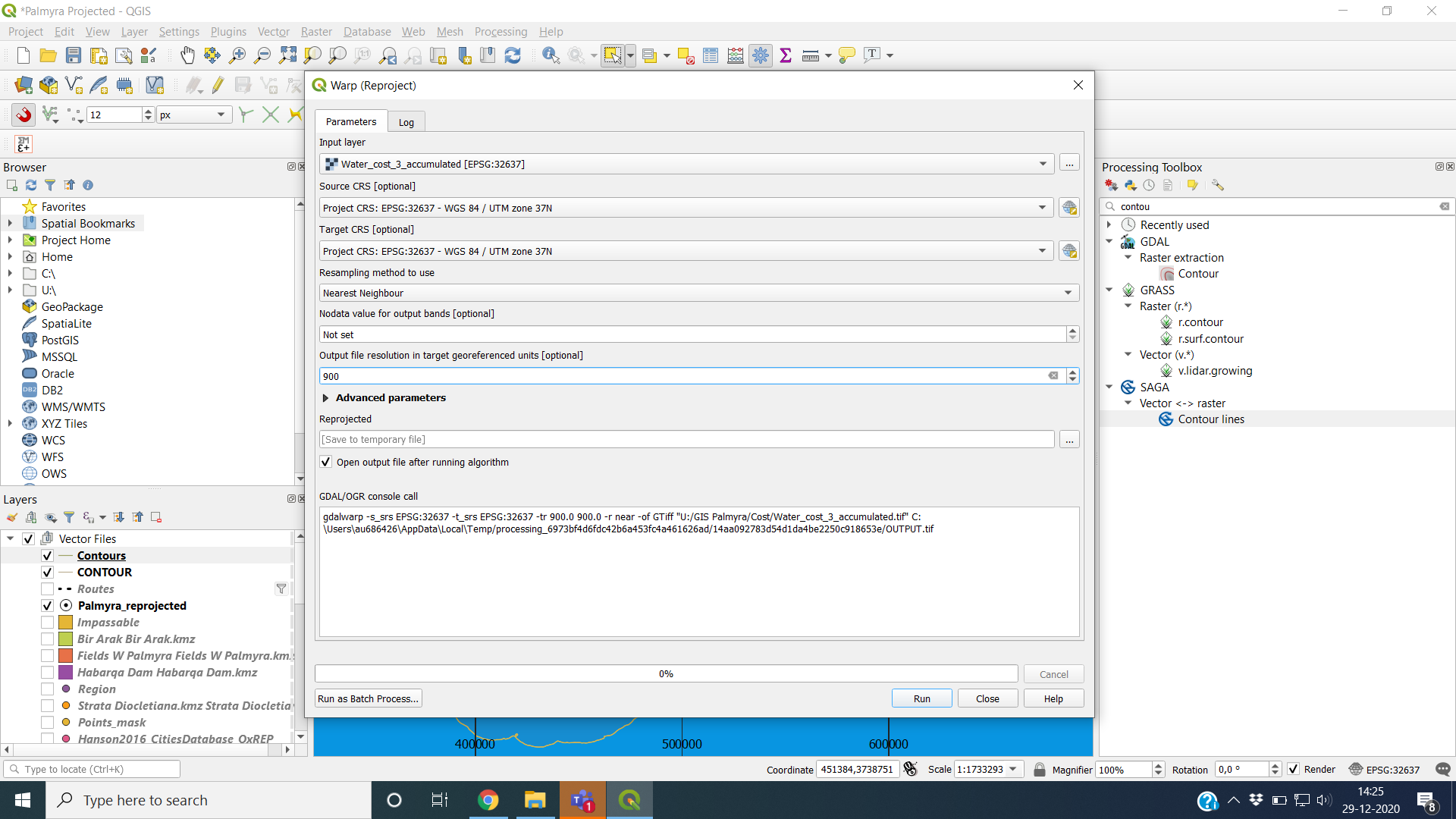
**

1. Once we have the lower resolution raster, we can now extract the contours

**Process: Contour Lines (Processing Toolbox> SAGA> Vector <-> Raster > Contour Lines)**

**Inputs:** .tif

**Parameters:**

- **Grid:** Water_Cost_3_reclassified_900
- **Vertex type:** [0] x, y
- **Minimum Contour Value:** 6
- **Maximum Contour Value:** 30
- **Equidistance:** 6

**Output:** Contours.shp


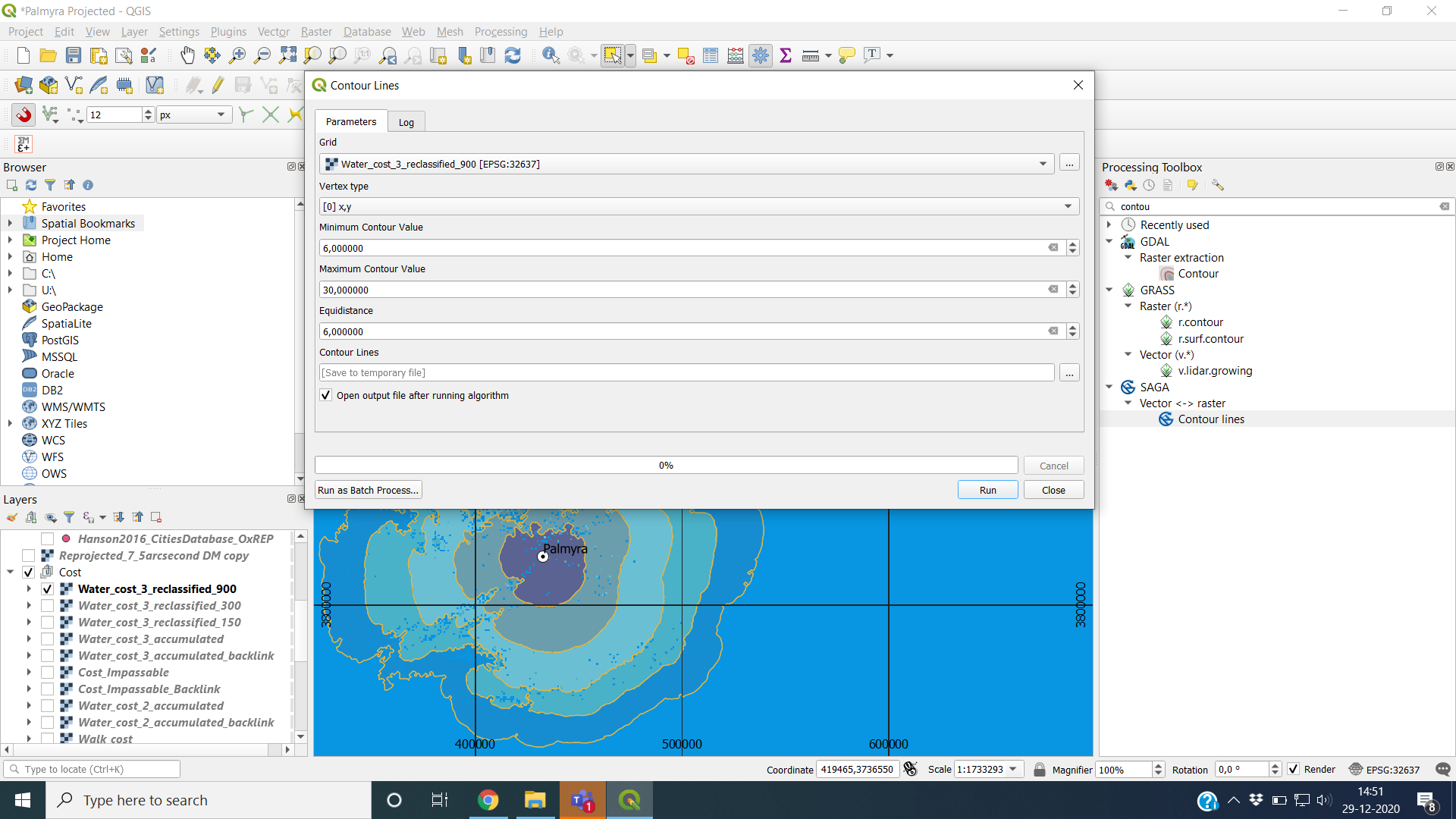


1. Now we must manually select the vector lines that envelop the areas we are interested in, more specifically, the 6, 18 and 30 intervals. Once we have them selected, we must save each in a different file.

**Process: Export> Save selected features as …**

**Input:** Contours.shp

**Parameters:**

- **Format:** ESRI Shapefile
- **File name:** Contour_6.shp/Contour_18.shp/Contour_30.shp
- **Layer name:** Leave Blank
- **CRS:** Project CRS: EPSG:32637 - WGS 84/ UTM zone 37N
- **Encoding:** UTF-8
- **Save only selected features:** yes
- **Select fields to export and their export options:** Select All
- **Geometry type:** Automatic
- **Extent:** Leave unchecked, as (current: layer)
- **Layer Options:** RESIZE = NO, SHPT = Leave blank
- **Custom options:** Leave blank

**Output:** Contour_6.shp, Contour_18.shp and Contour_30.shp

1. To make the polygons, the lines must first be simplified.

**Process: Line Simplification (Processing Toolbox> SAGA> Vector Line Tools> Line Simplification)**

**Inputs:** Contour_6.shp, Contour_18.shp and Contour_30.shp

**Parameters:**

- **Tolerance:** 1

**Output:** Contour_Near.shp, Contour_Medium.shp, Contour_Far.shp


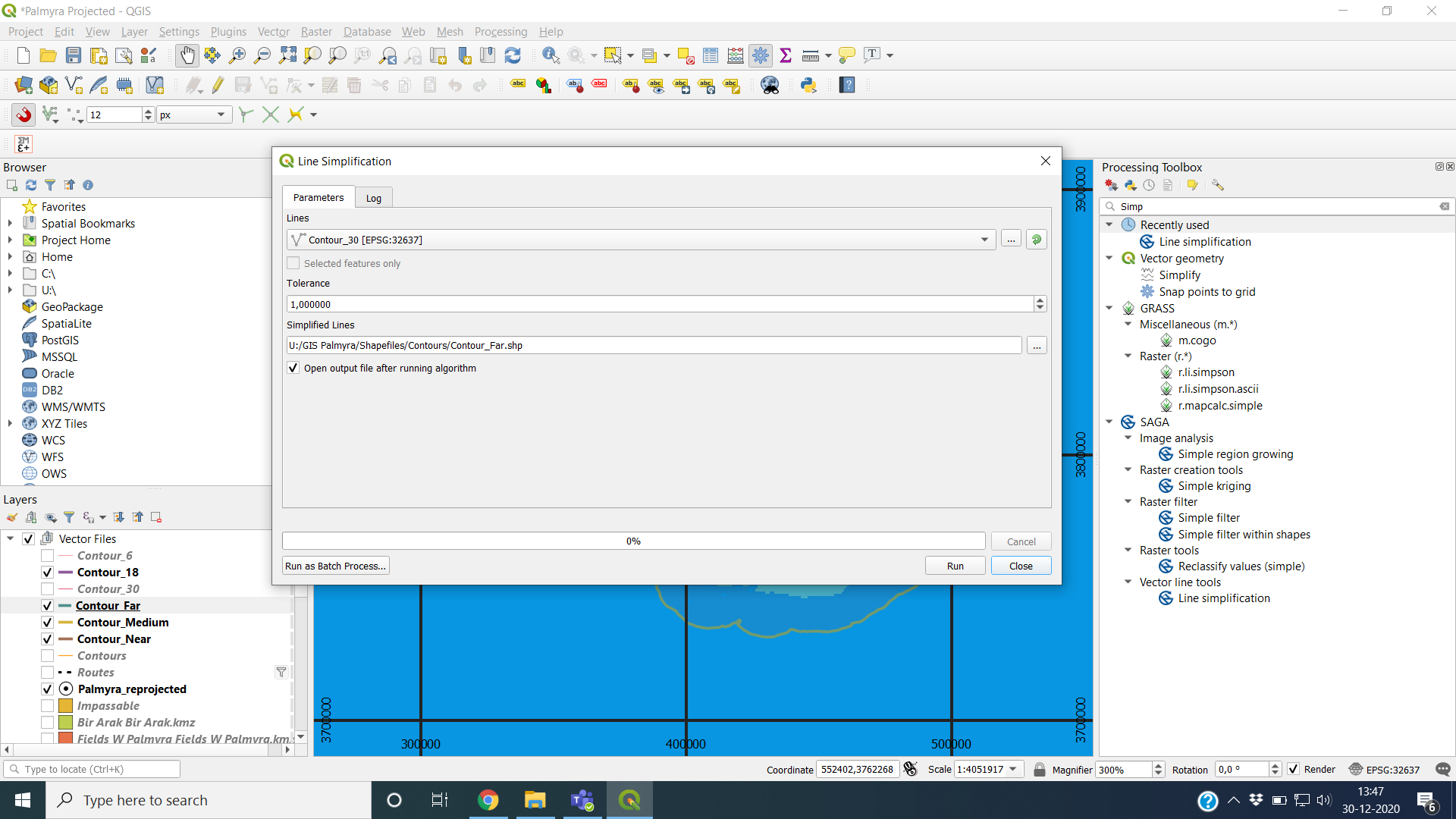


1. Finally, the Polygons need to be generated

**Process: Lines to Polygons (Processing Toolbox> Vector Geometry> Lines to Polygons)**

**Inputs:** Area_Near.shp, Area_Medium.shp, Area_Far.shp


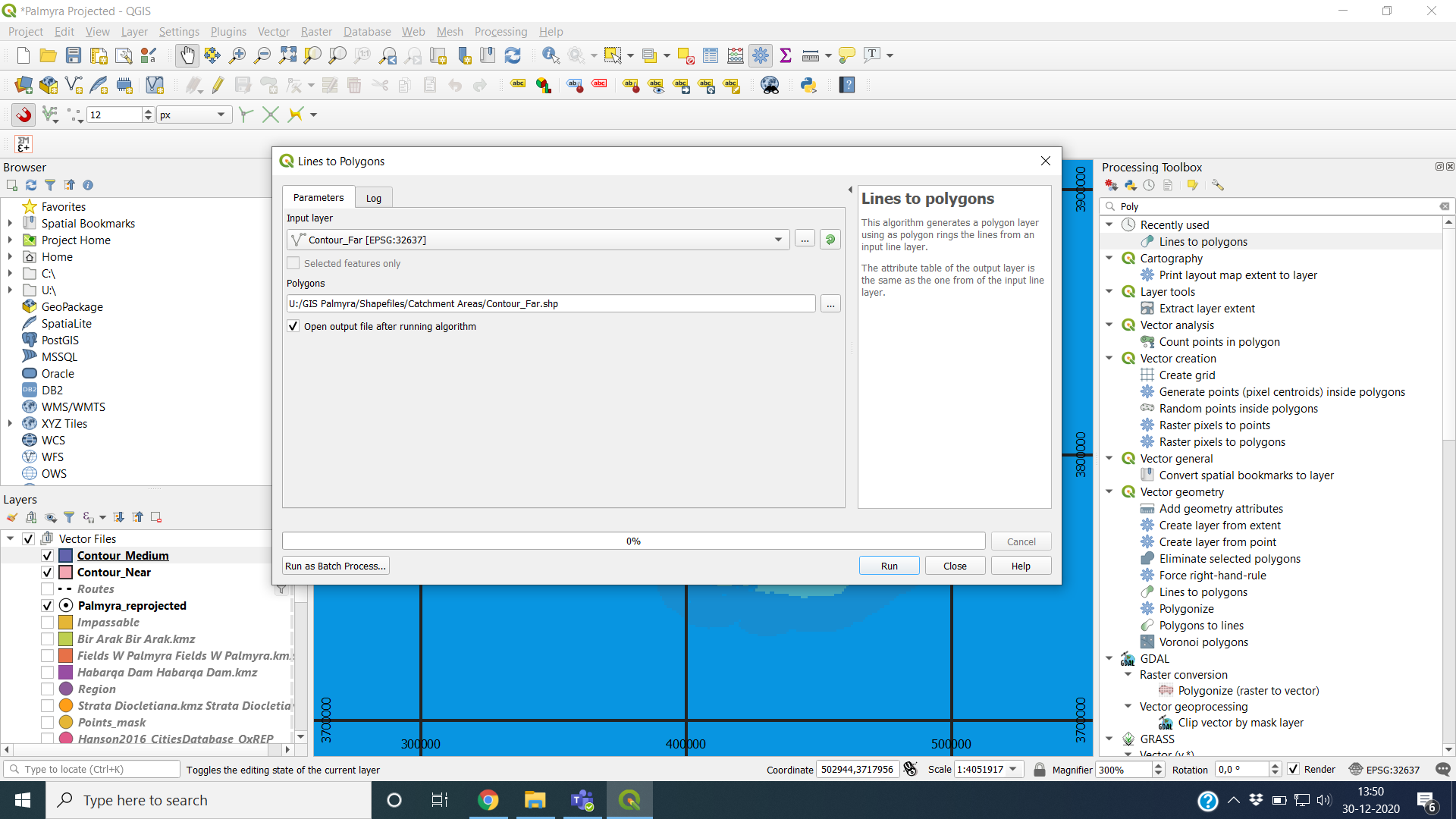


**Values (revised)**

| ***Step and Algorithm*** | ***Input*** | ***Parameter*** | ***Value*** |
| --- | --- | --- | --- |
| **STEP 18.1: Field Calculator (Processing Toolbox> Vector table> Field Calculator)** | SyrianDesertCisternPoolReservoir.shp | Formula or Expression | ‘Cistern’ |
| **STEP 18.1: Field Calculator (Processing Toolbox> Vector table> Field Calculator)** | SyrianDesertMajorWells.shp | Formula or Expression | ‘Major Well’ |
| **STEP 18.1: Field Calculator (Processing Toolbox> Vector table> Field Calculator)** | SyrianDesertMinorWells.shp | Formula or Expression | ‘Minor Well’ |
| **STEP 18.1: Field Calculator (Processing Toolbox> Vector table> Field Calculator)** | SyrianDesertSprings.shp | Formula or Expression | ‘Spring’ |
| **STEP 18.2: Field Calculator (Processing Toolbox> Vector table> Field Calculator)** | SyrianDesertCisternPoolReservoir.shp | Formula or Expression | ´0.7’ |
| **STEP 18.2: Field Calculator (Processing Toolbox> Vector table> Field Calculator)** | SyrianDesertMajorWells.shp | Formula or Expression | ´0.9’ |
| **STEP 18.2: Field Calculator (Processing Toolbox> Vector table> Field Calculator)** | SyrianDesertMinorWells.shp | Formula or Expression | ‘0.8’ |
| **STEP 18.2: Field Calculator (Processing Toolbox> Vector table> Field Calculator)** | SyrianDesertSprings.shp | Formula or Expression | ‘1’ |
| **STEP 20:** **Buffer (Processing toolbox> Vector geometry> Buffer)** | SyrianDesertWaterSources.shp | Formula or Expression | ‘10’ |
| **STEP 22: (Processing toolbox> GRASS> Raster (r.*)> r.null)** | Buffer_rasterized.tif | The value to replace the null value by (optional) | 0,5 |

**References**

1. USGS EROS Archive. Shuttle Radar Topography Mission (SRTM) 1 Arc-Second Global [Internet]. USGS EROS Archive - Digital Elevation - Shuttle Radar Topography Mission (SRTM) 1 Arc-Second Global. 2015 [cited 2020 Jul 11]. Available from: https://doi.org/10.5066/F7PR7TFT

2. Seland EH. Water Sources in the Syrian Desert [Internet]. V2 ed. DataverseNO. 2019 [cited 2020 Nov 24]. Available from: https://doi.org/10.18710/CEY9QR

3. Hanson JW. Cities Database (OXREP databases) Version 1.0 [Internet]. 2016 [cited 2020 Nov 24]. Available from: http://oxrep.classics.ox.ac.uk/databases/cities/; DOI: https://doi.org/10.5287/bodleian:eqapevAn8

4. Hanson JW. An Urban Geography of the Roman World, 100 BC to AD 300. Vol. 18. Oxford: Archaeopress Oxford; 2016.

5. 19th Field Survey Company, Royal Engineers, Service géographique, Forces françaises libres au Levant. Levant 1 / 500 000 : Provisional “Goings” Overprint [Internet]. Cairo: Middle East Drawing and Reproduction; 1942 [cited 2021 Aug 11]. Available from: https://nla.gov.au/nla.obj-2237684441/view

6. Tobler W. Three Presentations on Geographical Analysis and Modeling. National Center for Geographic Information and Analysis; 1993. Report No.: 93–1.

7. White DA. The Basics of Least Cost Analysis for Archaeological Applications. Adv Archaeol Pract. 2017/01/16 ed. 2015;3(4):407–14.
