# Supplementary material for: Food security in Roman Palmyra (Syria) in light of paleoclimatological evidence and its historical implications
Source: PLoS One. 2022 Sep 21;17(9):e0273241. doi: 10.1371/journal.pone.0273241 (PMC9491547; doi:10.1371/journal.pone.0273241)
Supplement: S3 File — It contains detailed instructions, as well as screenshots of the process and relevant references. (DOCX) [file pone.0273241.s003.docx]

Building the model

Version of QGIS: 3.10.11”A Coruña”, Grass

Version of operating system: Microsoft Windows 10 Education, Version 2004, Build 19041.985

QGis plug-ins:

- DB Manager (0.1.20)
- Fonctions SIGMOÉ (3.0.1)
- Georeferencer GDAL (3.1.9)
- MetaSearch Catalog Client (0.3.5)
- Processing (2.12.99)
- Point sampling tool (0.5.3)
- QuickMapServices (0.19.11.1)


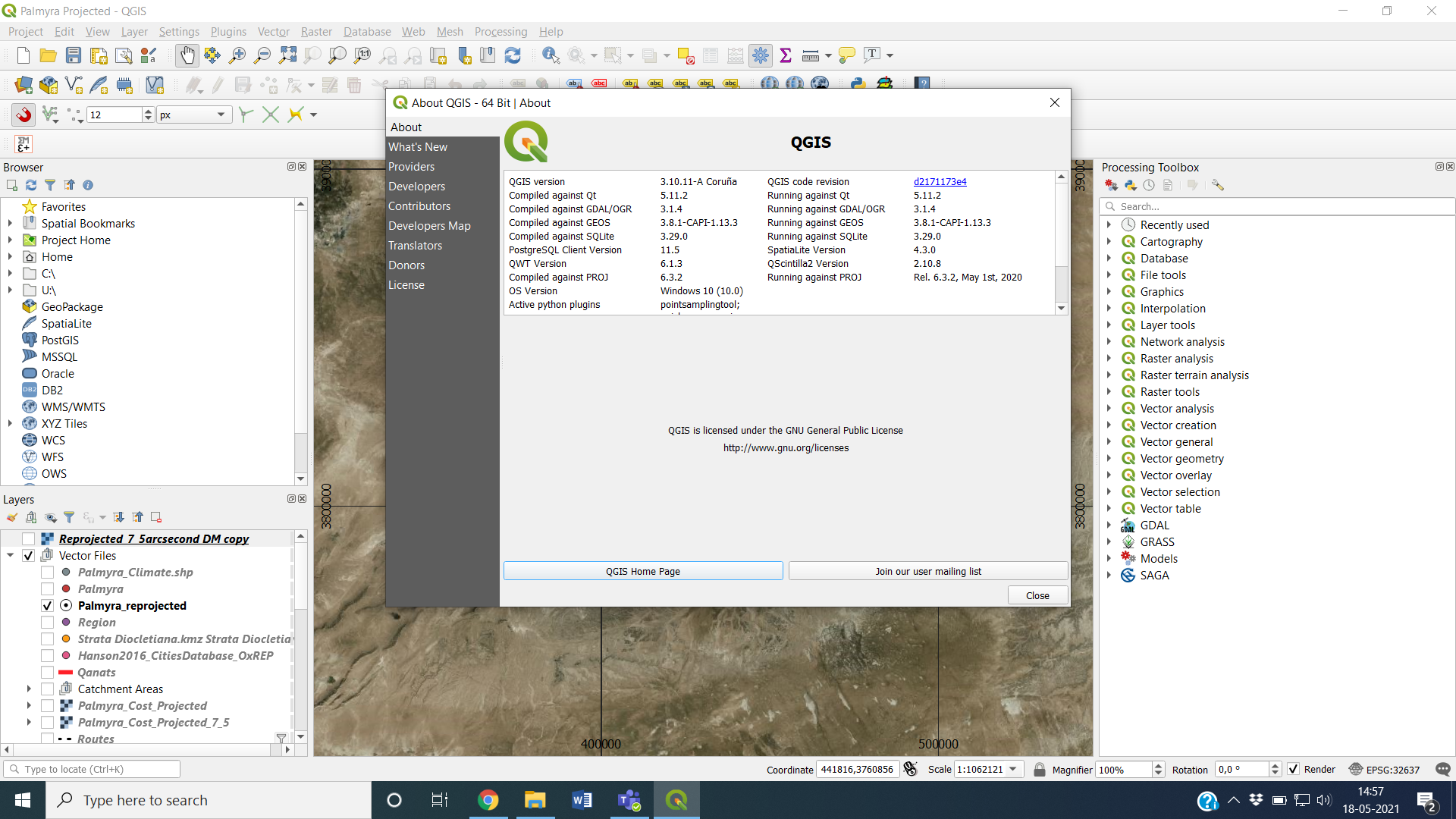


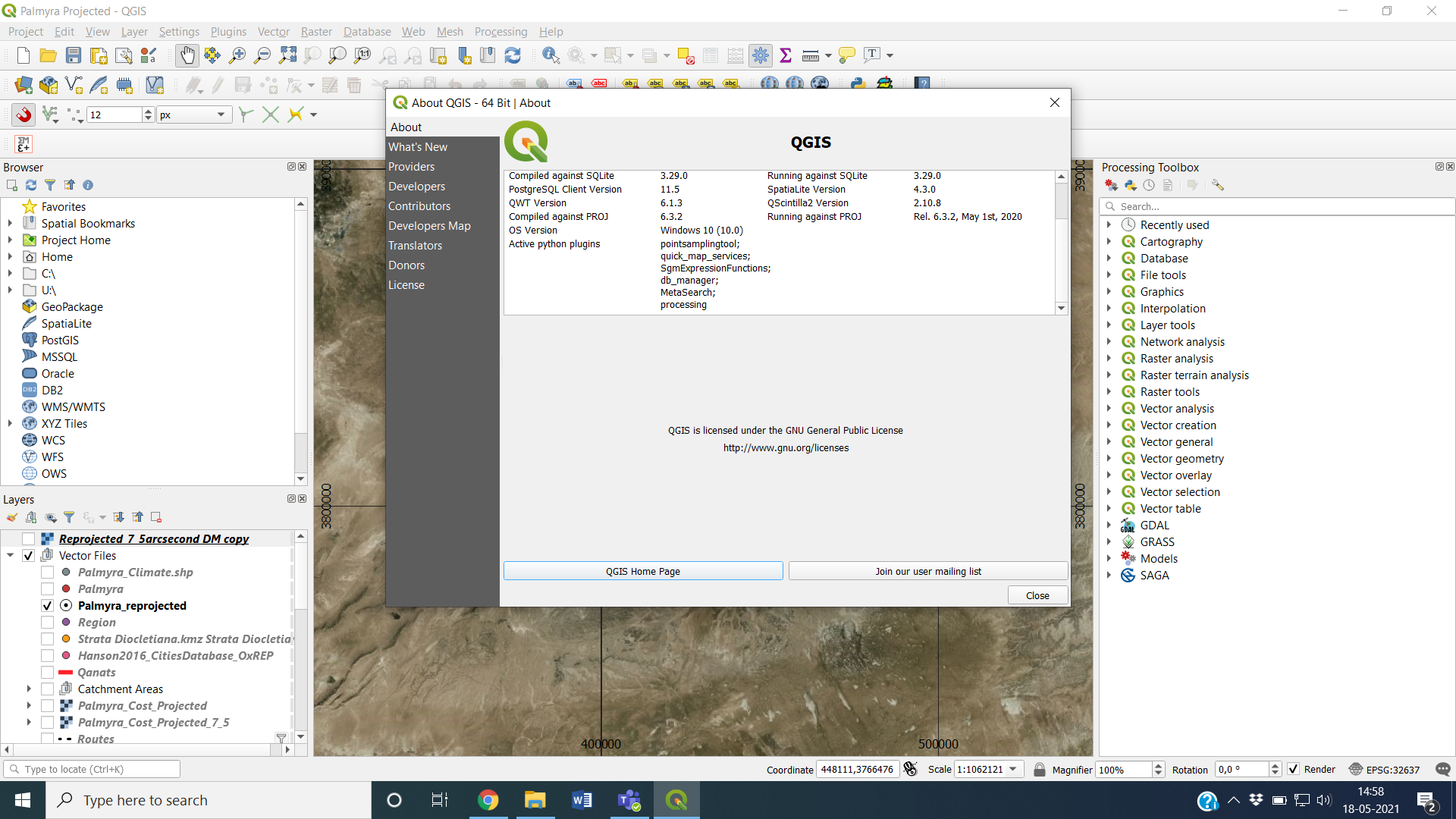


1. **Steps in QGIS**
2. Firstly, since the HWSD raster (1) is really large, covering all of the world, we need to cut it down. We will download a shapefile of the borders of Syria (2, the Administrative Boundaries of Syria, specifically, SYR_adm0.shp).

**Process: Clip Raster by Mask Layer (GDAL>Raster extraction>Clip Raster by Mask Layer)**

**Input:** hwsd.bil

**Parameters:**

- **Mask layer:** SYR_adm0.shp
- **Source CRS:** EPSG:4326-WGS 84
- **Target CRS:** EPSG:32637-WGS 84/UTM zone 37N
- **Assign a specified Nodata value to output bands:** Not set
- **Match the extent of the clipped raster to the extent of the mask layer:** Yes
- **Keep resolution of Input Raster:** Yes
- **All other parameters:** Leave blank or Not set

**Output:** HWSD_Syria.tif


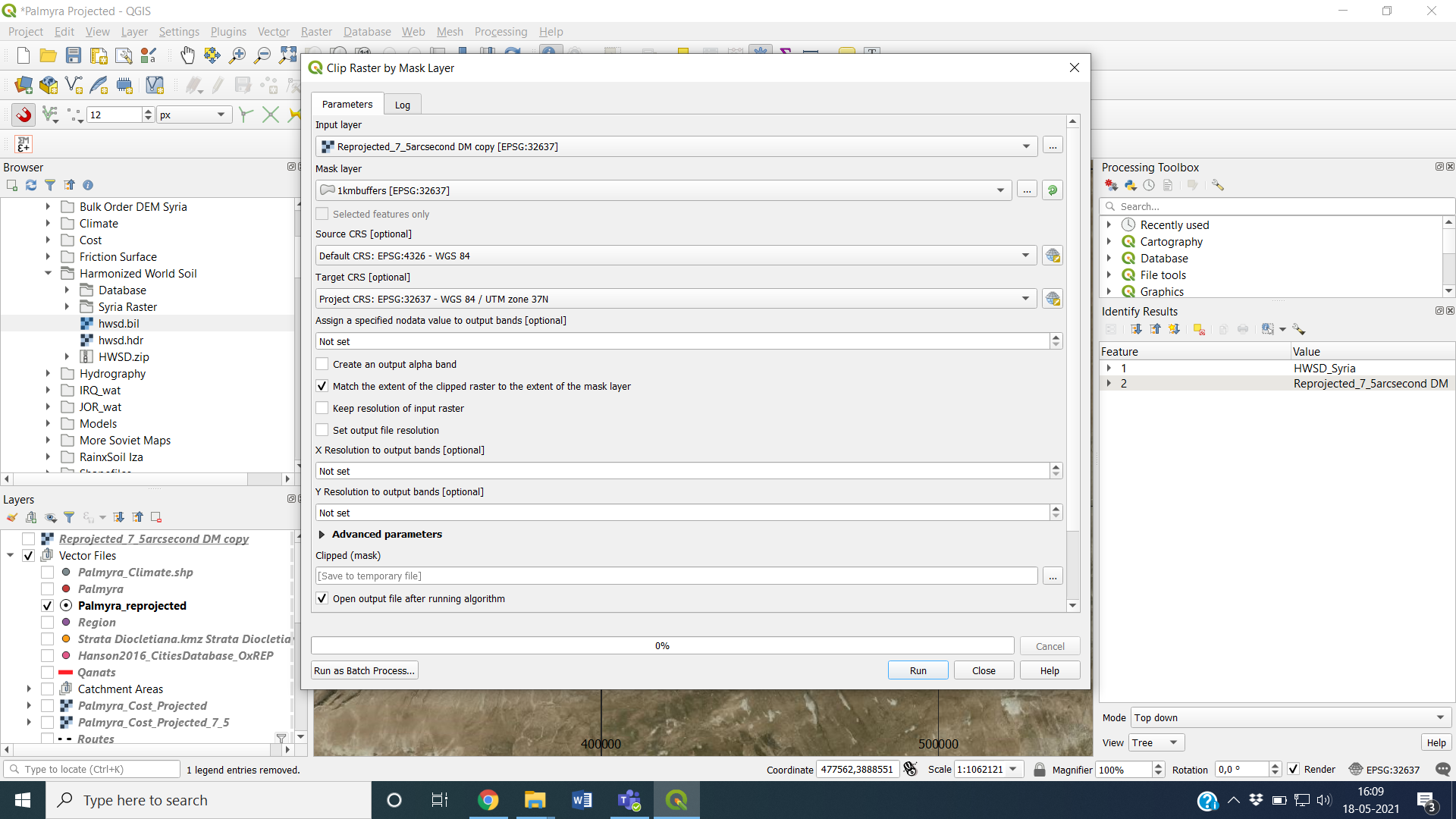


1. Now the raster needs to become a vector file.

**Process: Polygonize (Raster to Vector) (GDAL>Raster Conversion>Polygonize (Raster to Vector))**

**Input:** HWSD_Syria.tif

**Parameters:**

- **Band Number:** Band 1 (Gray)
- **Name of the field to create:** DN
  - **All other parameters:** Leave blank or Not set

**Output:** Soil_Polygons.shp


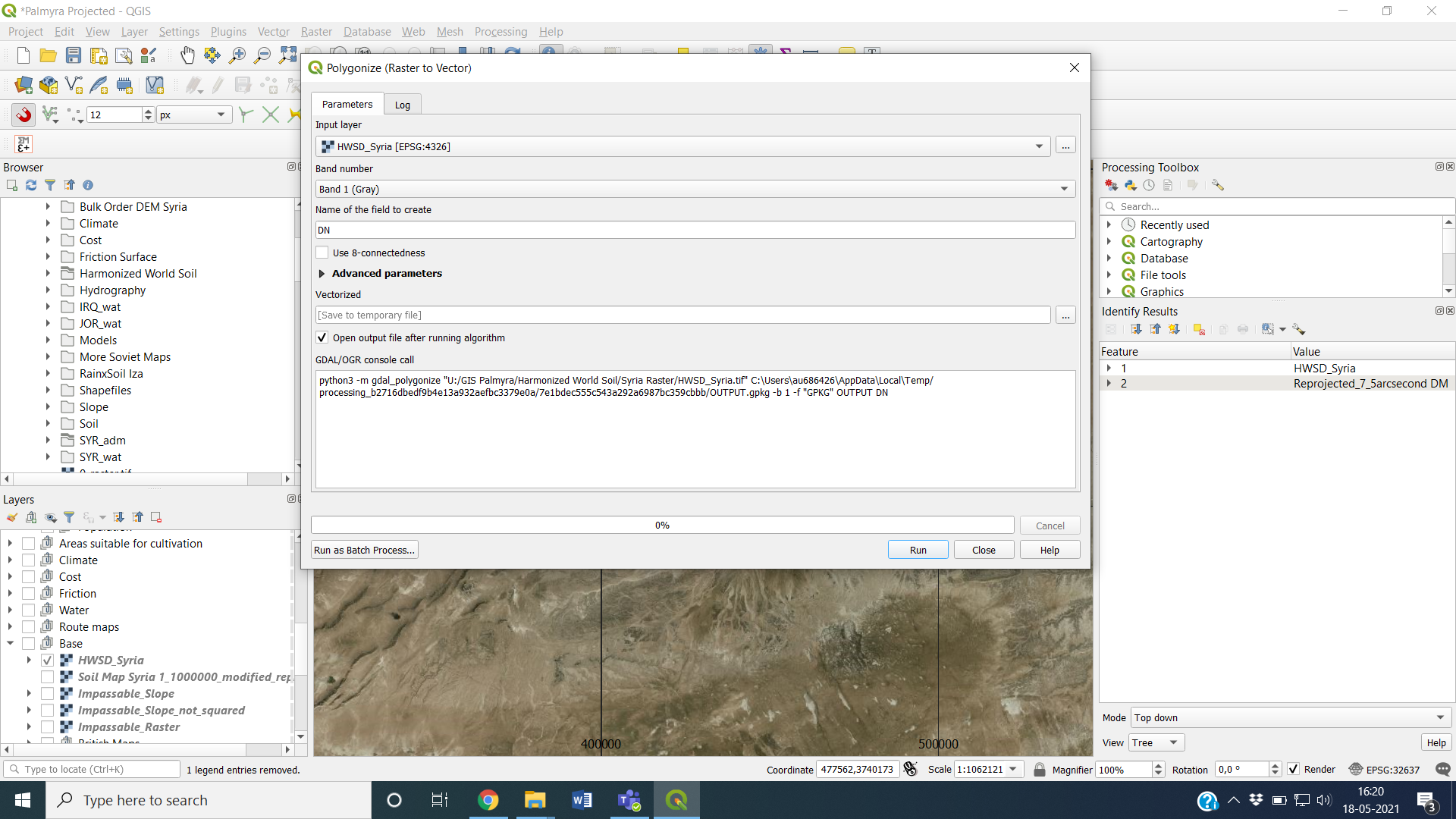


1. Reproject the polygons to a projected coordinate system

**Process: Reproject layer (QGIS>Vector General>Reproject layer)**

**Input:** Soil_Polygons.shp

**Parameters:**

- - **Target CRS:** EPSG:32637-WGS 84/UTM zone 37N

**Output:** Soil_Polygons_Reprojected.shp


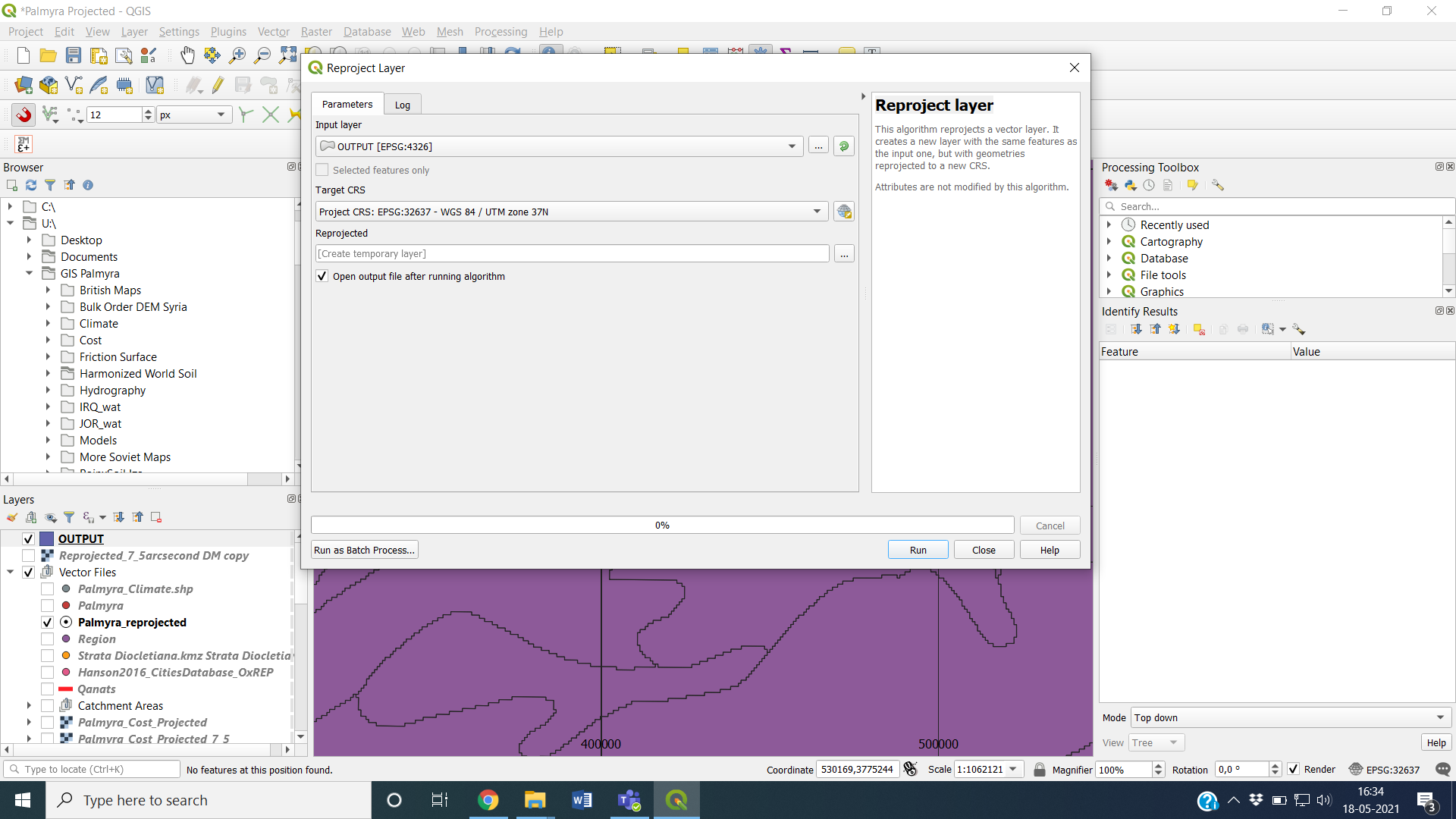


1. Now, we need to clip the soil areas with the cost envelopes for Palmyra, to establish the different soils within each of the envelopes.

**Process: Clip (QGIS>Vector Overlay>Clip)**

**Input:** Soil_Polygons_Reprojected.shp

**Parameters:**

- **Overlay layer:** Area_Far.shp, Area_Medium.shp, Area_Near.shp

**Output:** Temporary_Far.shp, Temporary_Medium.shp, Temporary_Near.shp


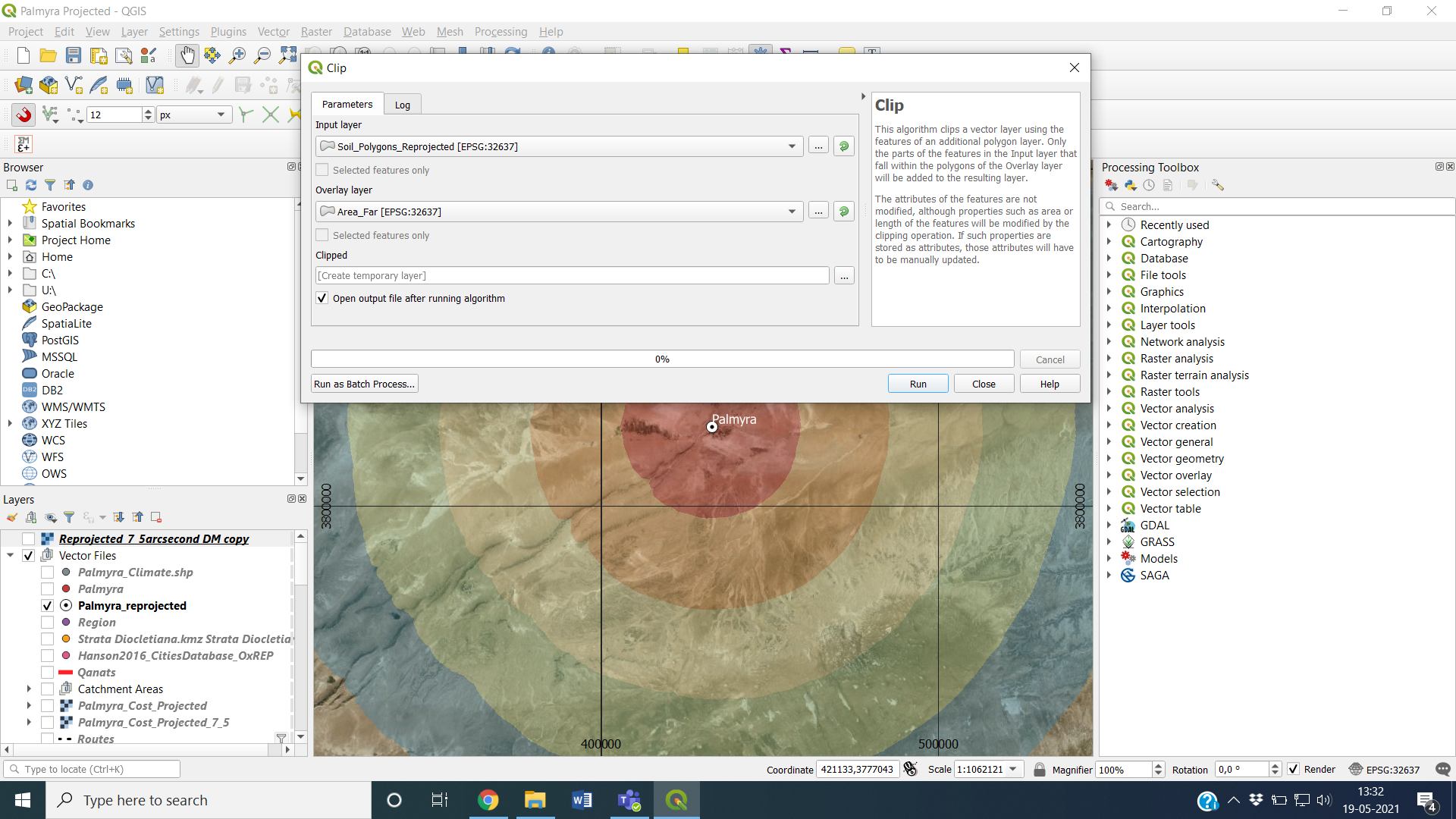


Some redundant, or repeated polygons may have to be erased.

1. Now, the areas for all of these polygons should be calculated.

**Process: Field calculator (QGIS>Vector table>Field calculator)**

**Input:** Temporary_Far.shp, Temporary_Medium.shp, Temporary_Near.shp

**Parameters:**

- **Create new field:** Yes
- **Output field name:** Area
  - **Output field type:** Float
  - **Output field width:** 20
  - **Precision:** 3
- **Expression:** $area

**Output:** Temporary_Far.shp, Temporary_Medium.shp, Temporary_Near.shp

1. The same should be done with the Cost Envelopes

**Process: Field calculator (QGIS>Vector table>Field calculator)**

**Input:** Area_Far.shp, Area_Medium.shp, Area_Near.shp

**Parameters:**

- **Create new field:** Yes
- **Output field name:** Area
  - **Output field type:** Float
  - **Output field width:** 20
  - **Precision:** 3
- **Expression:** $area

**Output:** Area_Far.shp, Area_Medium.shp, Area_Near.shp


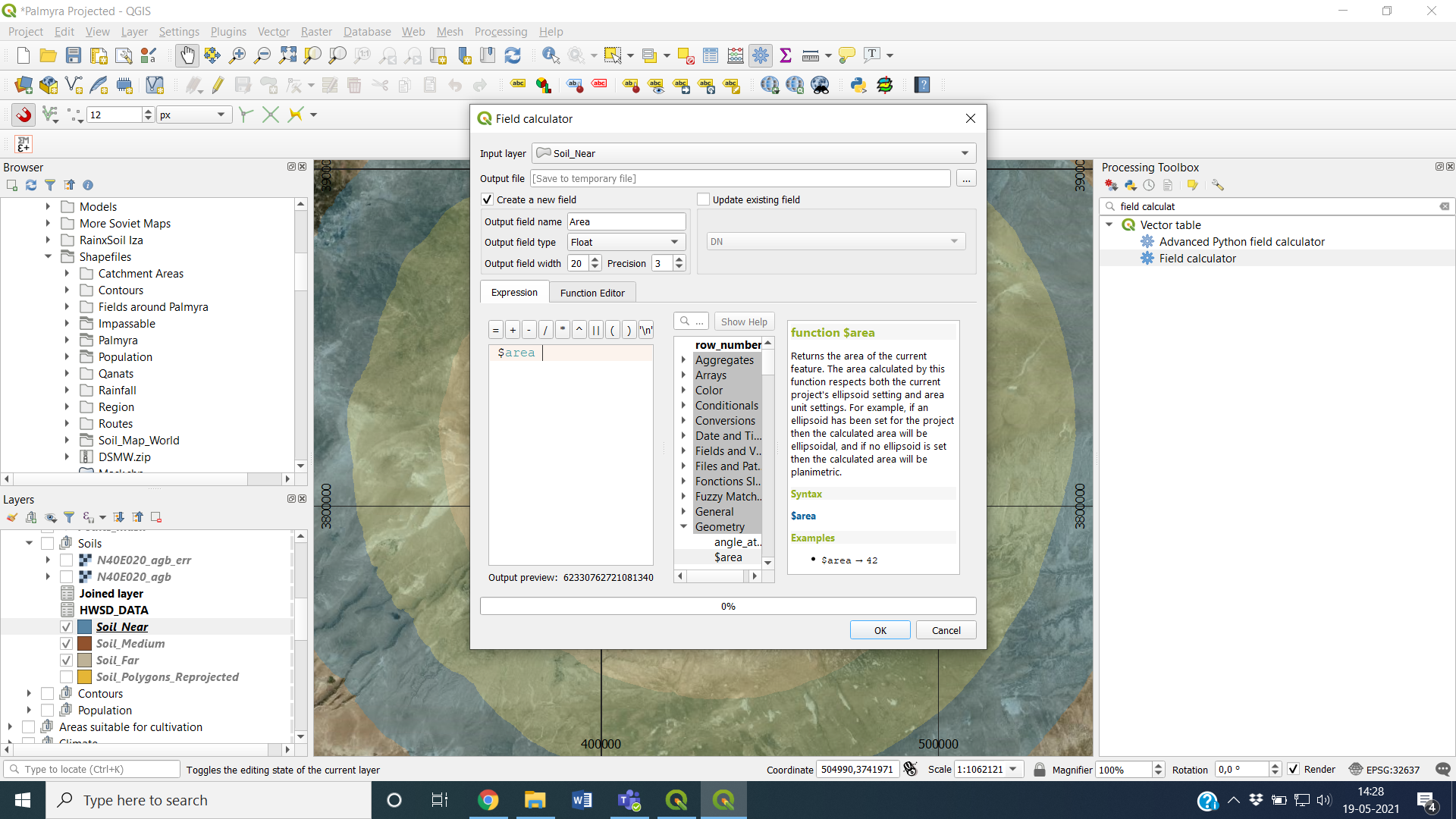


1. Now, the different tables from the database need to be joined in order to concentrate the relevant information and link it to the soil polygons. There are several ways to do this, ideally using the query builder in Microsoft Access to get a customized table, as the manual for the HWSD suggests. It could theoretically also be done in QGIS itself, by joining tables to each other in 1-1 joins. Both methods did not work for us, for reasons unknown.

Instead, we only linked the main table of the database (HWSD_DATA) to the soil polygons in a 1-N join. All the codes were then interpreted from the other tables in the database with the help of the manual. This is slow and should be avoided, but we were not able to. However, the method does not affect the result, only the speed at which it is reached.

Insert the main table of the database (HWSD_DATA) into QGIS.

**Process: Join attributes by field value (QGIS>Vector General> Join attributes by field value)**

**Input:** Temporary_Far.shp, Temporary_Medium.shp, Temporary_Near.shp

**Parameters:**

- **Table Field:** DN
- **Input Layer 2:** HWSD_DATA
  - **Table Field 2:** MU_GLOBAL
  - **Layer 2 fields to copy:** Leave blank
  - **Join type:** Create separate feature for each matching feature (one to many)
- **All other parameters:** Leave blank or Not set

**Output:** Soil_Far.shp, Soil_Medium.shp, Soil_Near.shp


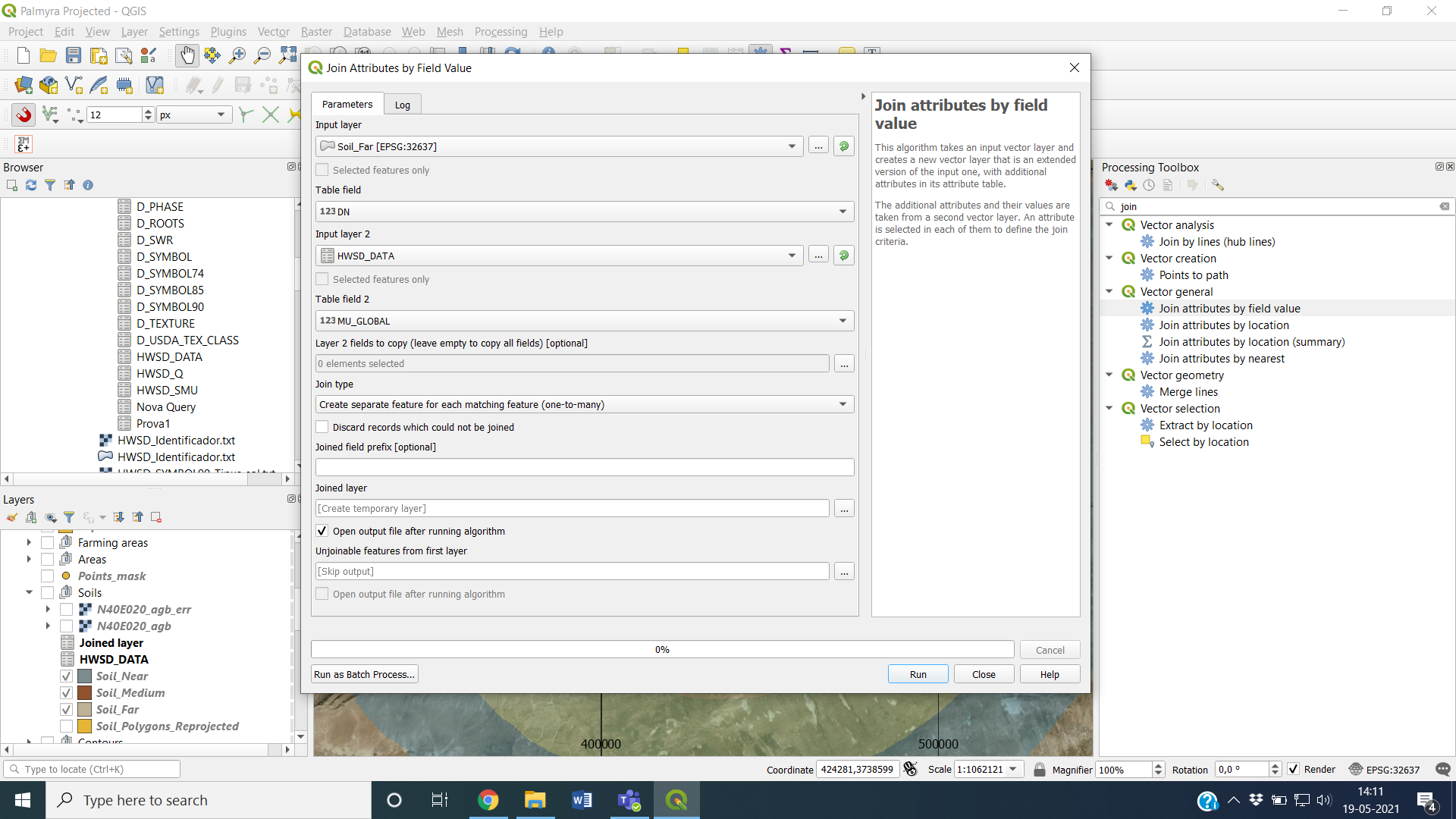


1. All of the monthly 30-year averages 10 minute rasters for maximum temperature, minimum temperature, and precipitation from WorldClim (3) must be introduced into QGIS.
2. Merge all montly precipitation rasters to create a yearly Rainfall raster.

**Process: Raster calculator (QGIS>Raster analysis>Raster Calculator)**

**Input:** wc2.1_10m_prec_01@1.tif, wc2.1_10m_prec_02@1.tif, wc2.1_10m_prec_03@1.tif, wc2.1_10m_prec_04@1.tif, wc2.1_10m_prec_05@1.tif, wc2.1_10m_prec_06@1.tif, wc2.1_10m_prec_07@1.tif, wc2.1_10m_prec_08@1.tif, wc2.1_10m_prec_09@1.tif, wc2.1_10m_prec_10@1.tif, wc2.1_10m_prec_11@1.tif, wc2.1_10m_prec_12@1.tif

**Parameters:**

- **Expression:** "wc2.1_10m_prec_01@1" + "wc2.1_10m_prec_02@1" + "wc2.1_10m_prec_03@1" + "wc2.1_10m_prec_04@1" + "wc2.1_10m_prec_05@1" + "wc2.1_10m_prec_06@1" + "wc2.1_10m_prec_07@1" + "wc2.1_10m_prec_08@1" + "wc2.1_10m_prec_09@1" + "wc2.1_10m_prec_10@1" + "wc2.1_10m_prec_11@1" + "wc2.1_10m_prec_12@1"
- **Predefined expressions:** NDVI
- **Reference layer(s):** wc2.1_10m_prec_01@1.tif
- **All other parameters:** Leave blank or Not set

**Output:** Yearly_precipitation.tif


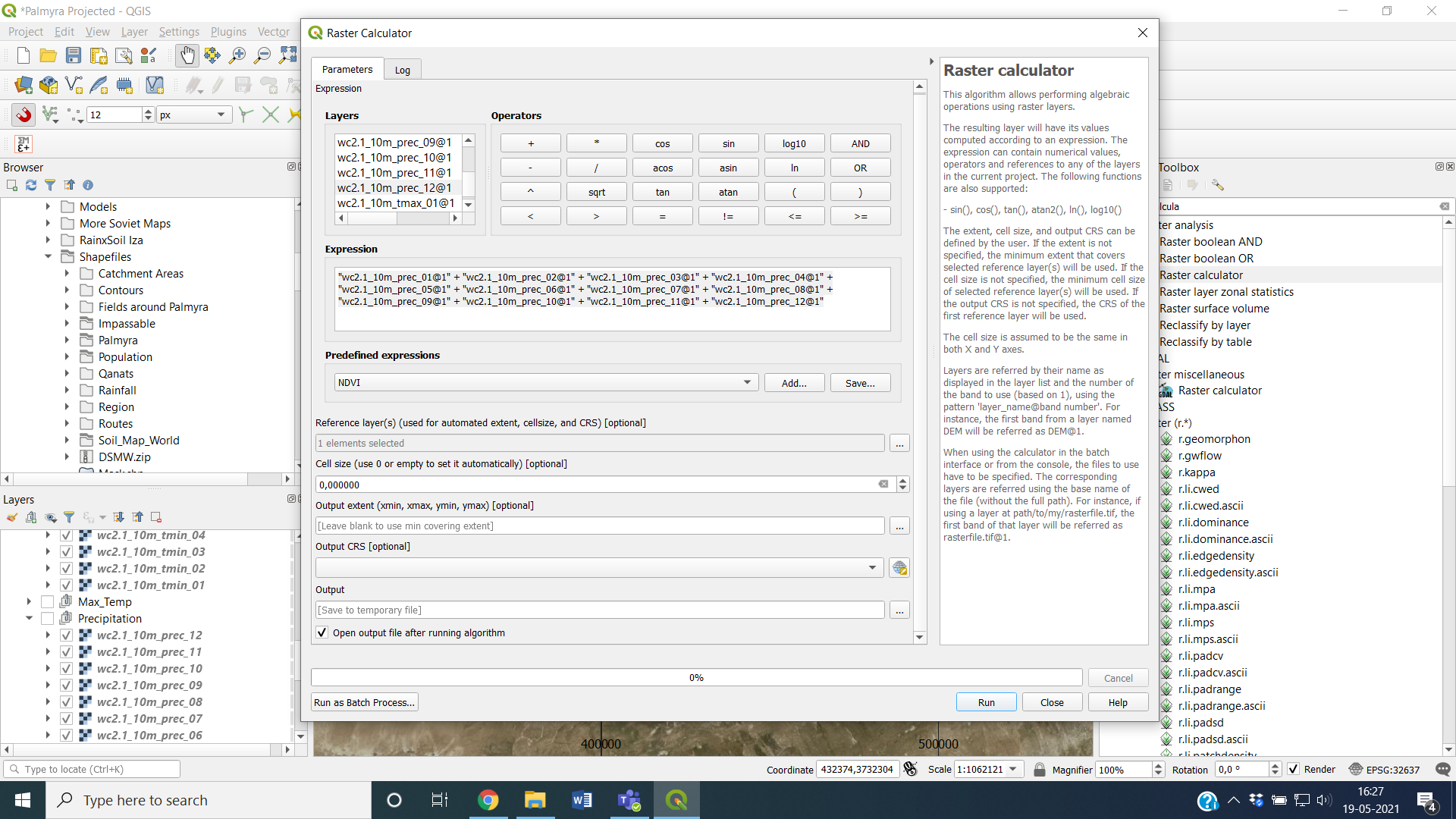


1. Rainfall contours will have to be extracted.

**Process: Contour (GDAL>Raster extraction>Contour)**

**Input:** Yearly_precipitation.tif

**Parameters:**

- **Band number:** Band 1 (Gray)
- **Interval between colour lines:** 25
  - **Attribute name:** ELEV
  - **Offset from zero relative to which to interpret interval:** 0
  - **All other parameters:** Leave blank or Not set

**Output:** Rainfall.shp


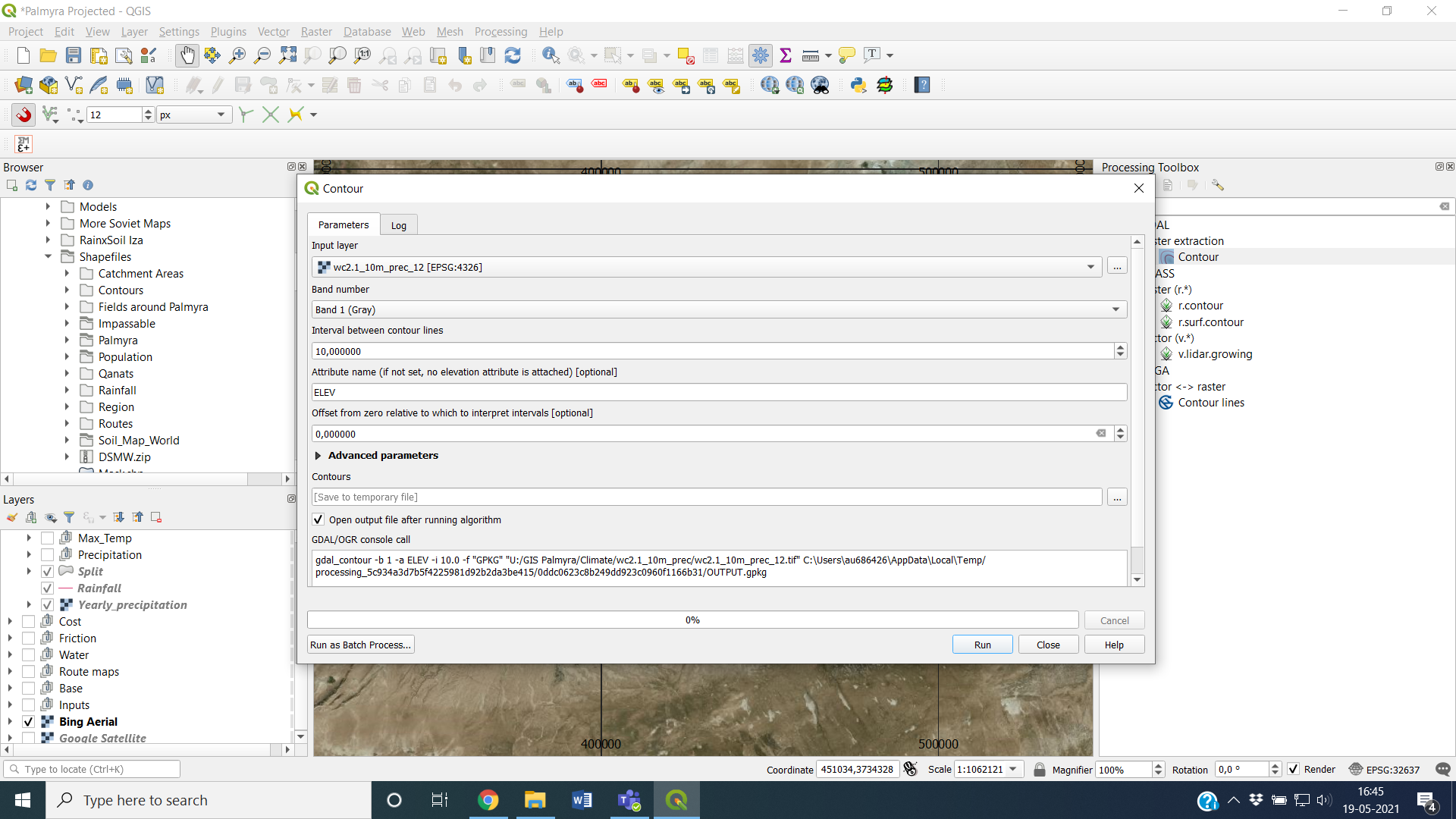


1. Point data for a few places needs to be taken. This data does not need to be extracted from any QGIS algorithm. The data needed is the following, and it all comes from the formerly introduced WorldClim Rasters:

Point 1: E 370775, N 3811664 (N of Harbaqah Dam)

| Mes | Precipitació (mm) | Temp min | Temp màx |
| --- | --- | --- | --- |
| Gener | 24 | 1,36 | 10,8965 |
| Febrer | 15 | 2,38 | 13,54775 |
| Març | 13 | 5,24 | 18,15625 |
| Abril | 14 | 9,42 | 23,82125 |
| Maig | 10 | 14,13 | 29,35325 |
| Juny | 0 | 17,74 | 33,54925 |
| Juliol | 0 | 19,61 | 35,4675 |
| Agost | 0 | 19,99 | 35,59 |
| Setembre | 0 | 17,06 | 32,67975 |
| Octuble | 8 | 12,32 | 27,14875 |
| Novembre | 16 | 6,05 | 19,163 |
| Desembre | 19 | 2,54 | 12,70025 |
| Total | 119 |  |  |

Point 2: E 433049, N 3823355 (Palmyra)

| Mes | Precipitació (mm) | Temp min | Temp màx |
| --- | --- | --- | --- |
| Gener | 23 | 1,6645 | 10,94 |
| Febrer | 15 | 2,8738 | 13,833 |
| Març | 14 | 5,8505 | 18,34125 |
| Abril | 16 | 10,7073 | 24,5435 |
| Maig | 9 | 15,5333 | 30,08575 |
| Juny | 0 | 19,2615 | 34,31225 |
| Juliol | 0 | 21,3000 | 36,56225 |
| Agost | 0 | 21,3745 | 36,3135 |
| Setembre | 0 | 18,4180 | 33,41725 |
| Octuble | 6 | 13,1593 | 27,575 |
| Novembre | 16 | 6,4133 | 19,223 |
| Desembre | 18 | 2,8540 | 12,57075 |
| Total | 117 |  |  |

Point 3: E 460701, N 3827401 (Fields south of Bir Arak)

| Mes | Precipitació (mm) | Temp min | Temp màx |
| --- | --- | --- | --- |
| Gener | 21 | 1,6445 | 11,29975 |
| Febrer | 13 | 3,06775 | 14,45875 |
| Març | 13 | 6,1205 | 18,9655 |
| Abril | 15 | 11,06525 | 25,385 |
| Maig | 9 | 15,8145 | 31,0625 |
| Juny | 0 | 19,755249 | 35,43275 |
| Juliol | 0 | 21,9365 | 37,77875 |
| Agost | 0 | 21,82775 | 37,45425 |
| Setembre | 0 | 18,608751 | 34,4425 |
| Octubre | 6 | 13,26075 | 28,29775 |
| Novembre | 14 | 6,458 | 19,78325 |
| Desembre | 16 | 2,94 | 13,054 |
| Total | 107 |  |  |

Point 4: E 401610, N 3848312 (Wadi Jihar)

|  | |
| --- | --- |
| Mes | Precipitació (mm) |
| Gener | 30 |
| Febrer | 21 |
| Març | 20 |
| Abril | 23 |
| Maig | 14 |
| Juny | 0 |
| Juliol | 0 |
| Agost | 0 |
| Setembre | 0 |
| Octuble | 10 |
| Novembre | 19 |
| Desembre | 26 |
| Total | 163 |

Point 5: E 409596, N 3835763 (Jebel Abyad)

|  | |
| --- | --- |
| Mes | Precipitació (mm) |
| Gener | 23 |
| Febrer | 14 |
| Març | 14 |
| Abril | 16 |
| Maig | 9 |
| Juny | 0 |
| Juliol | 0 |
| Agost | 0 |
| Setembre | 0 |
| Octuble | 7 |
| Novembre | 15 |
| Desembre | 18 |
| Total | 116 |

Point 6: E 432888, N 3851734 (Wadi Abyad)

| Wadi Abyad | |
| --- | --- |
| Mes | Precipitació (mm) |
| Gener | 28 |
| Febrer | 20 |
| Març | 19 |
| Abril | 22 |
| Maig | 13 |
| Juny | 0 |
| Juliol | 0 |
| Agost | 0 |
| Setembre | 0 |
| Octuble | 9 |
| Novembre | 18 |
| Desembre | 23 |
| Total | 152 |

1. For the area of the fields, the layer “Bing Aerial”, from Bing Maps must be introduced into the project, from the QuickMapServices plug-in. Alternatively, the freely available SPOT CIB-10 satellite imagery (4) can be used, both are useful. With these, the former 20th century fields can be measured roughly with the “Measure Area” tool. This does not need to be too precise, since it is merely to establish a plausible upper limit to to size of fields in Bir Arak, At-Tarqa, the Oasis and around the Umayyad Garden in Harbaqah.


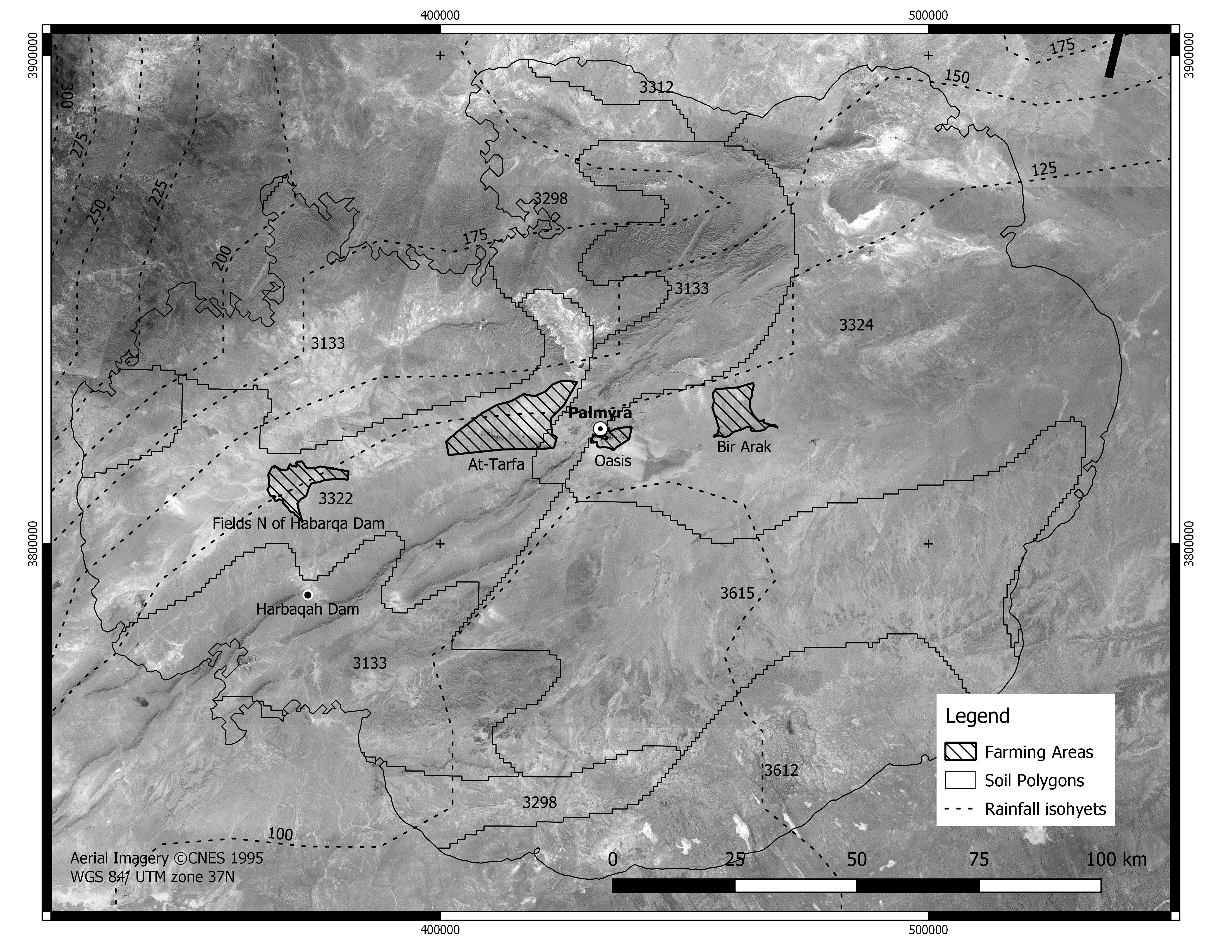


Our numbers are as follows:

Bir Arak: 76.887.840,326 m2

At Tarqa: 208.920.842,362 m2

Oasis: 22.818.400,823 m2

Fields North of Harbaqah: 81.103.873,011 m2

Another visible thing by superimposing the Soil_Far.shp and the aerial imagery is what soils they had:

Bir Arak: 3324 (MU_GLOBAL)

At Tarqa: 3322 (MU_GLOBAL)

Oasis: 3324 (MU_GLOBAL)

Fields North of Harbaqah: 3322 (MU_GLOBAL)

1. Finally, catchment areas for rainfall that could irrigate said areas can be measured directly on the Bing aerial maps layer. The areas measured for the irrigation of At-Tarqa are Jebel Abyad, Wadi Abyad, and Wadi Jihar. They can also be measured directly with the “Measure Area” tool.


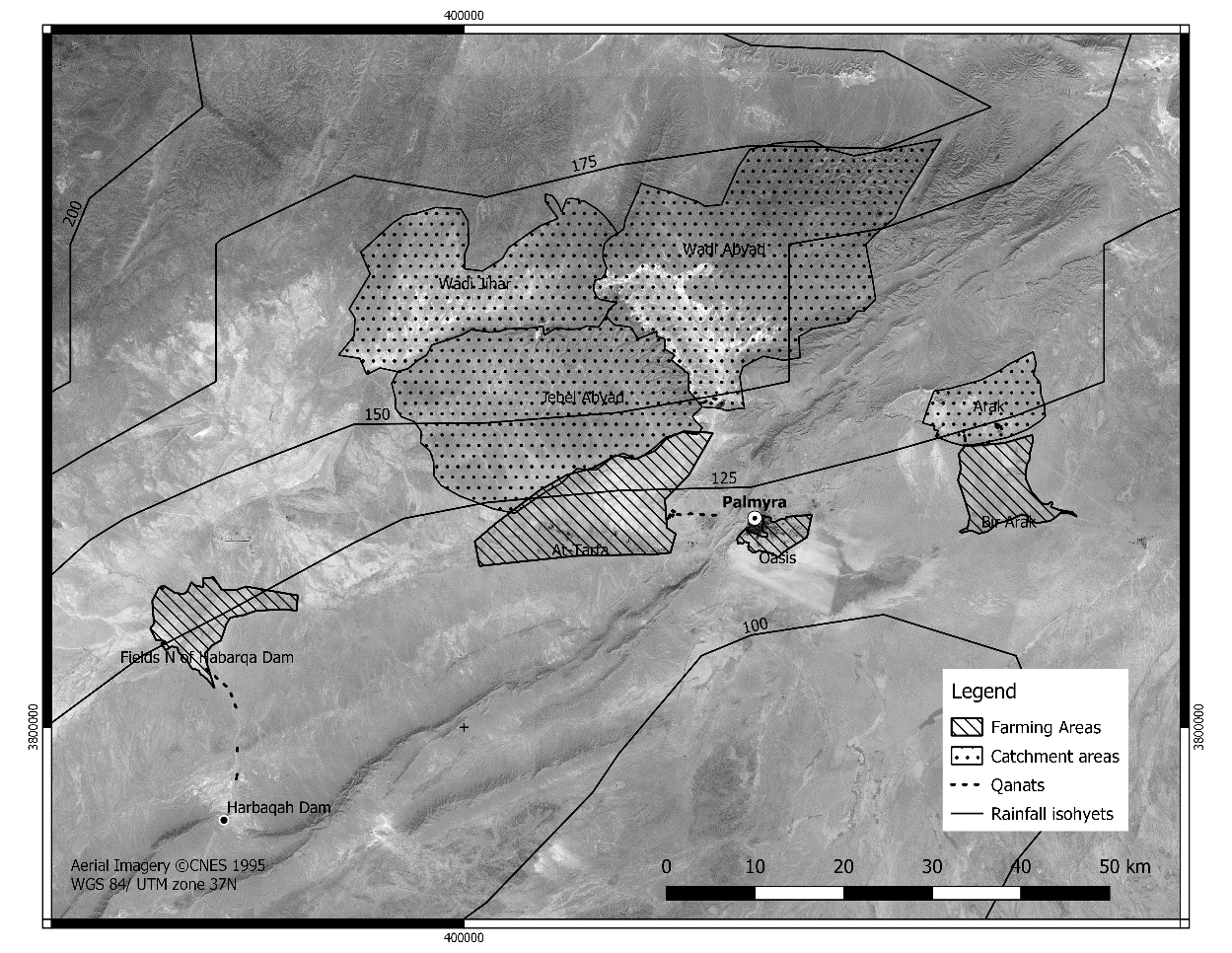


These are our numbers:

Arak: 9.420,266 ha

Jebel Abyad: 51.250,874 ha

Wadi Abyad: 60.138,6 ha

Wadi Jihar: 34.051,985 ha

These numbers will necessarily be approximate, and that is not a problem. Alternatively, shapefiles can be created to then measure the area. We did it to be able to create maps for documentation, but otherwise it is not necessary.

1. **Steps in AquaCrop**

AquaCrop 6.1


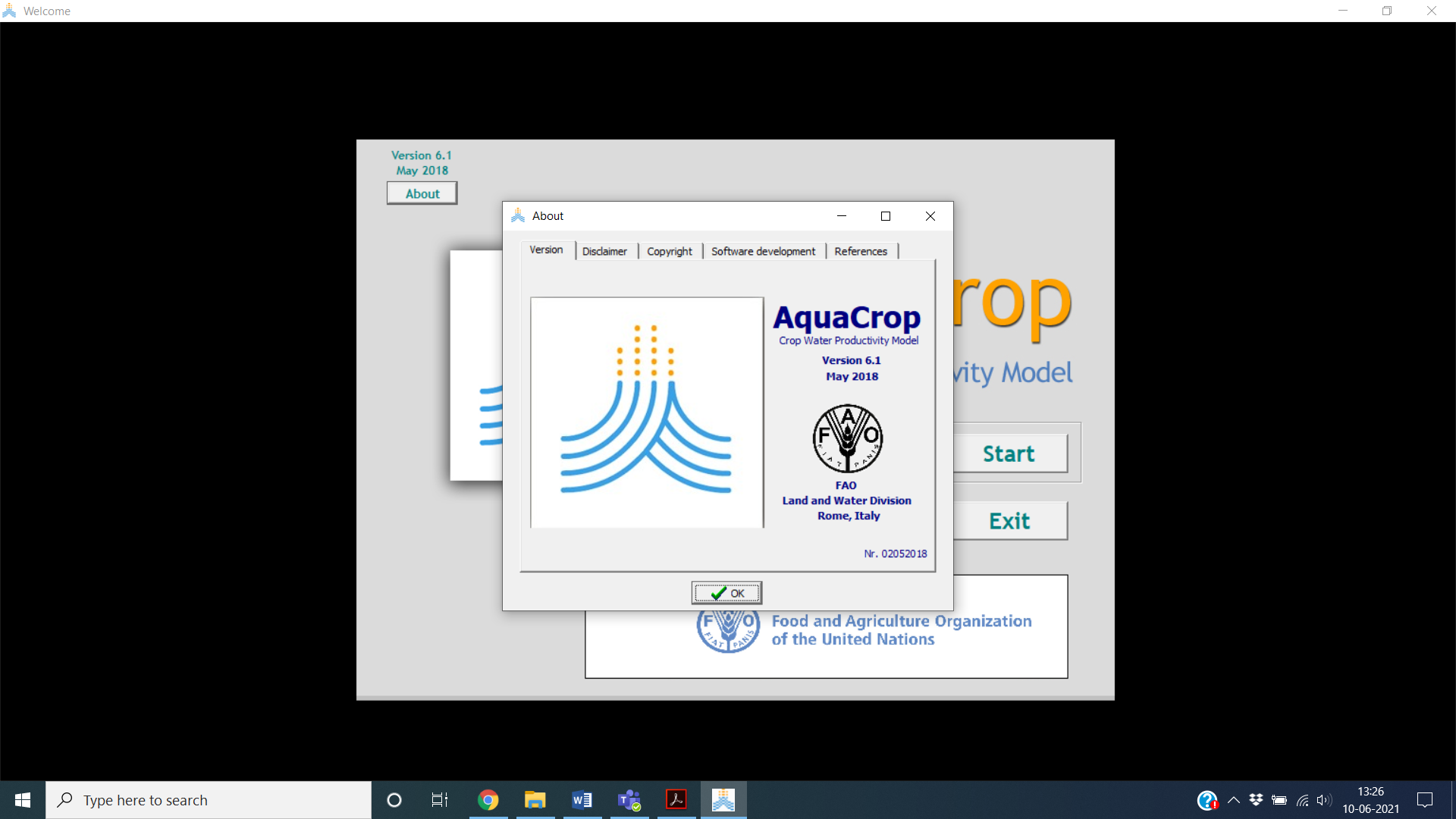


It is necessary to calculate the kg/ha outputs of all of the different soil areas for each of the 3 cost envelopes, as well as for each of the four main Farming areas around Palmyra. This requires preparing all of the files to input into the AquaCrop model (5–7). We will create a Soil file for each of the soils in the sequence of all soil areas (34 files), three climate files, two irrigation files and a field management file. All other files are going to be omitted or come included in the Program.

1. We will first create the three climate files. When selecting the climate file, we will select “Create Climate File”. First we will select the Rain file, and select “Create a new rain file”.

- **Input:** Monthly
- **NOT linked to a specific year:** Yes
- **From:** January
- **To:** December

We then will select “Create”. In the following table, we will introduce the Monthly rainfall data we have collected previously in QGIS (3).

This process needs to be repeated three times, creating each time a rainfall file for Palmyra, Bir Arak and the fields N of Harbaqah.

We will then select the Evapotranspiration (ETo) file, and select “Create a new ETo file”.

- **Input:** Monthly
- **NOT linked to a specific year:** Yes
- **From:** January
- **To:** December

We will then select “Create”. In the following table, we will introduce the Monthly ETo values for 2003 from Brunel et al.(8). We will then have created our Evapotranspiration (ETo) file for Palmyra.

Next, the Temperature files need to be created. We will select “temp” and then “Create a new temperature file”.

- **Input:** Monthly
- **NOT linked to a specific year:** Yes
- **From:** January
- **To:** December

We will then select “Create”. In the following table, we will introduce the Monthly Max and Monthly Min temperatures we have collected previously in QGIS (3).

This process needs to be repeated three times, creating each time a Temperature file for Palmyra, Bir Arak and Harbaqah.

With all of the Necessary files generated, the Climate files can now be assembled. For the atmospheric CO2, we will use world averages. Three files need to be created:

- **Inputs:**
  - Rain_Palmyra.PLU
  - ETo_Palmyra.ETo
  - Temp_Palmyra.TMP
  - Global_Average.CO2
- **Output:** Climate_Palmyra.CLI
- **Inputs:**
  - Rain_Arak.PLU
  - ETo_Palmyra.ETo
  - Temp_Arak.TMP
  - Global_Average.CO2
- **Output:** Climate_Arak.CLI
- **Inputs:**
  - Rain_Harbaqah.PLU
  - ETo_Palmyra.ETo
  - Temp_Harbaqah.TMP
  - Global_Average.CO2
- **Output:** Climate_Harbaqah.CLI


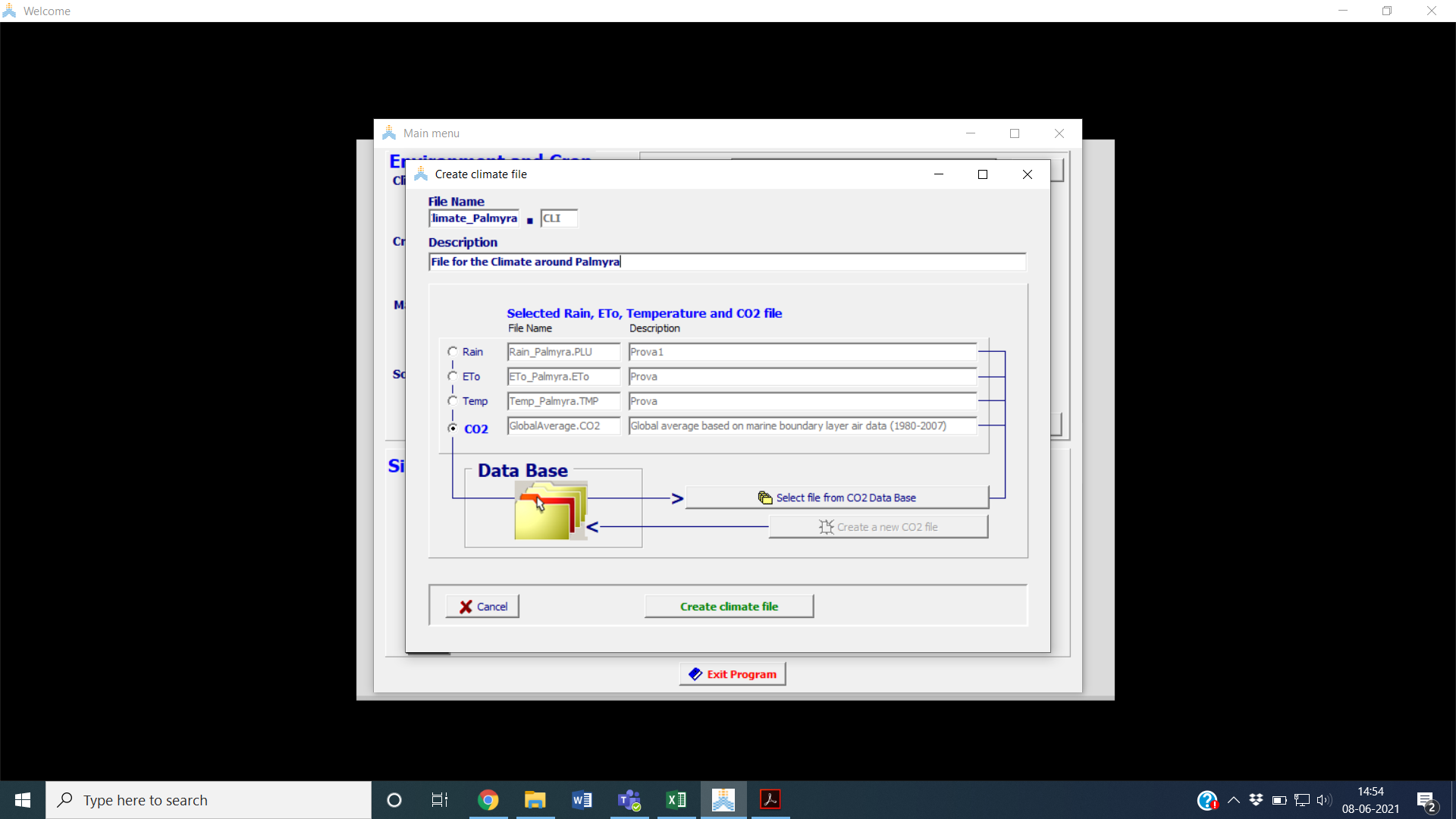


1. Now, two irrigation files have to be generated. “Generation of irrigation schedule” must be selected, and then “Create irrigation Schedule”.

- **Irrigation method:** Surface Irrigation - Border Irrigation
- **Percentage of soil surface wetted:** 100%
- **Time and Depth Criteria:**
  - **Valid from:** Day. No. 1
  - **Time Criteria>When?** (Interval:93 days)
  - **Fixed net application:** 75/275 mm
  - **Water quality:** Excellent

These steps have to be repeated twice, once for a file with 75 mm of irrigation, and another for 275 mm. These will be used to calculate the outputs of two different types of irrigation, extensive and intensive (200-400 mm respectively). It is impractical to create an irrigation file for each of possible necessary irrigation, since it varies with rainfall. This is why we assume the need for 75 and 275 mm, since this are the values needed to reach 200 and 400 mm if rainfall is 125 mm.


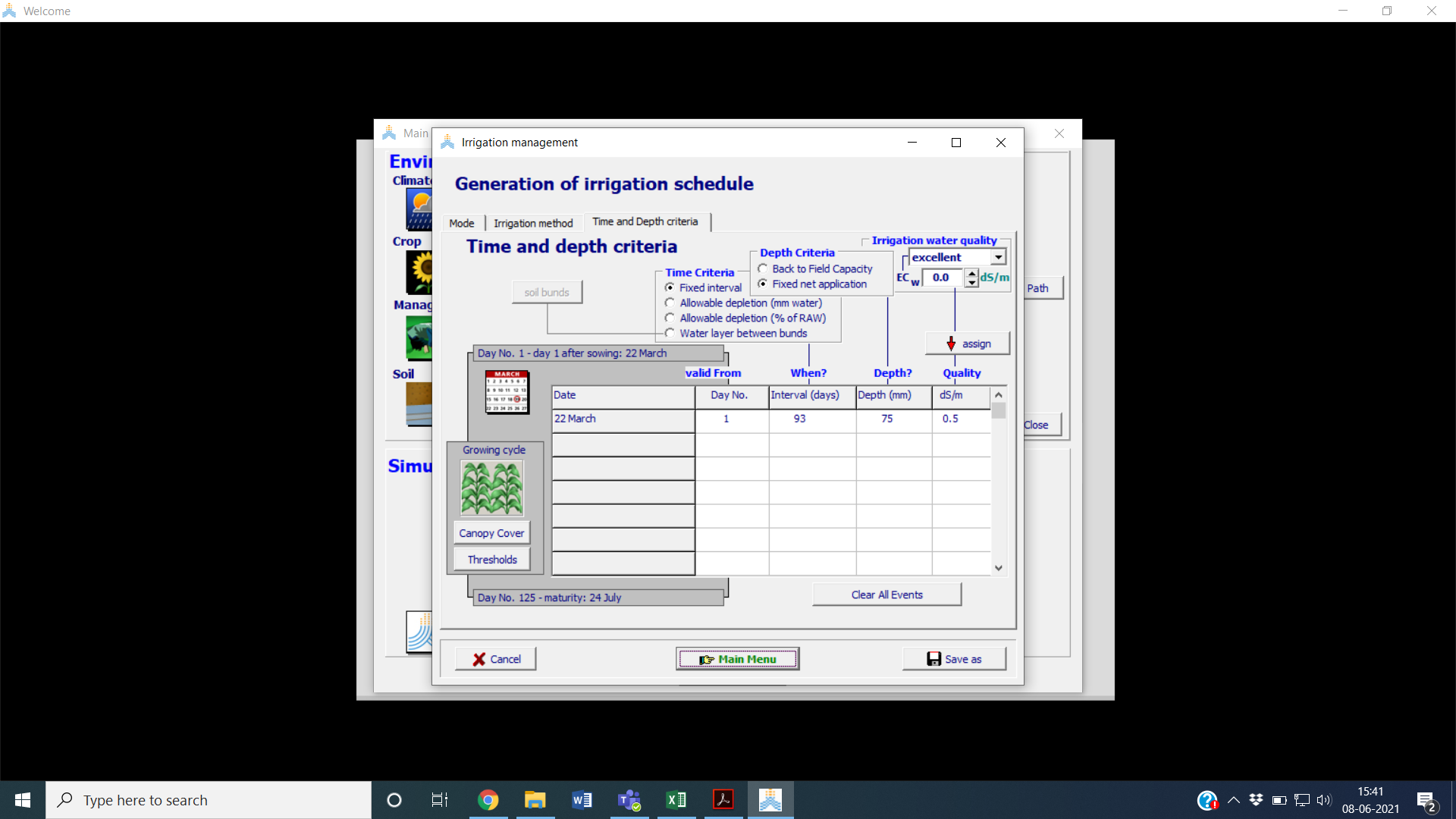


1. “Create field management File”.

- **Soil fertility:** non limiting, 100%
- **Soil surface cover:** none, 0%
- **Field surface practices, practices preventing runoff:** none
- **Soil bunds:** none
- **Weed management:** good (relative cover of weeds 15%)


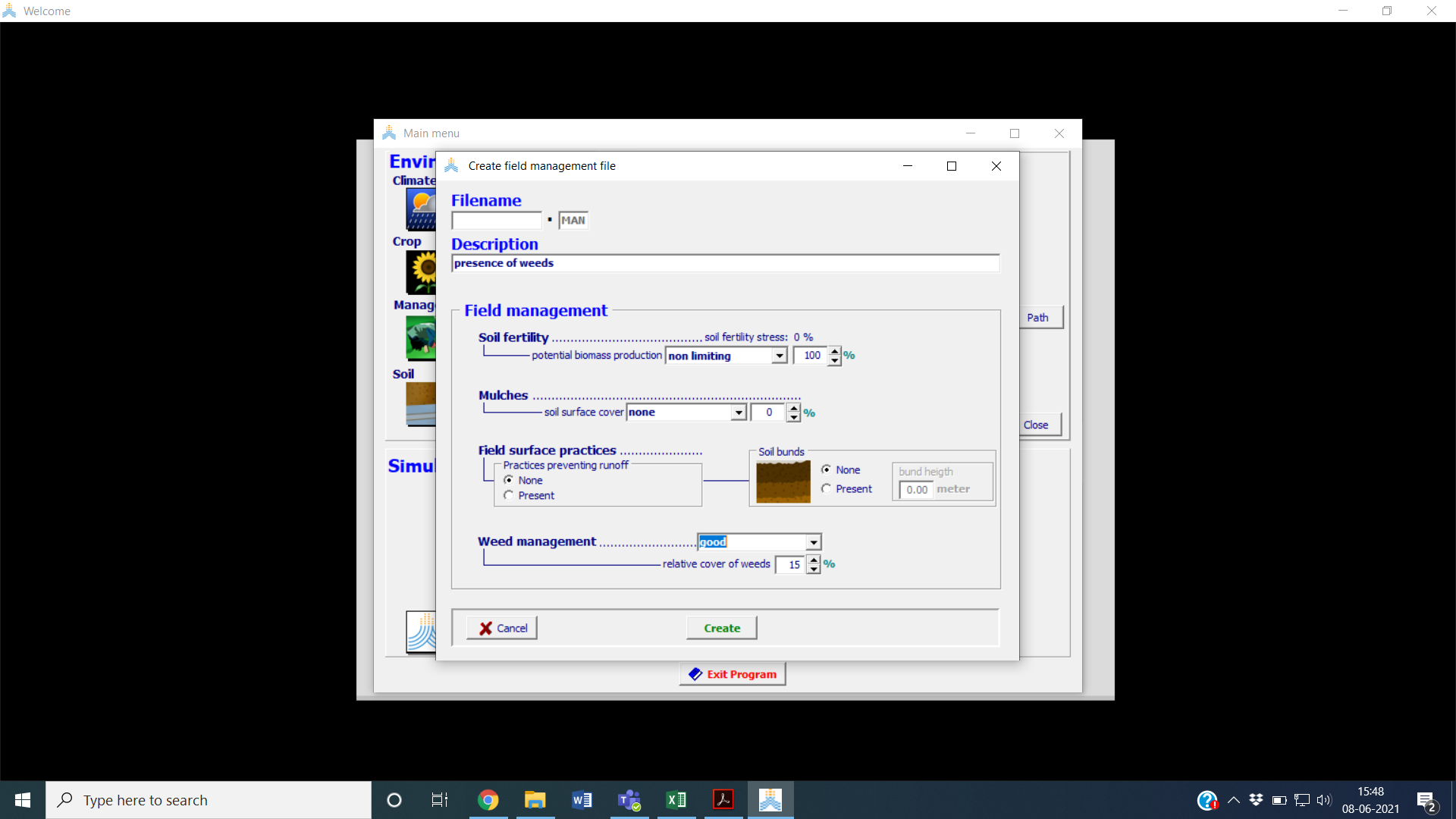


Only one such files is necessary.

1. Now, “Soil profile” files for each single type of soil in our cost envelopes must be created, for a total of 26 files. “Create Soil Profile file” must be selected.

- **Number of horizons:** 2
- **Soil textural Class1:** X
- **Thickness:** 0.10
- **Soil textural Class2:** Y
- **Thickness:** 1.00

Here, X is equivalent to the value corresponding to the soil type in the “T_USDA_TEX” attribute, and Y is the value of the soil type in the “S_USDA_TEX” attribute of the HWSD table (1,9).

Next, each file must be selected. After that, the the next step is “Display/Update soil profile characteristics” The “Characteristics of soil horizons” tab must then be accessed.

For each soil layer:

- **Soil water:** FC must be tweaked until the TAW matches the “AWC_Class” values
- **Stoniness:** Mass of gravel % must be set to the value of the attribute “T_GRAVEL” and “S_GRAVEL” for the first and second layers respectibly.
- **All other values:** Leave as they are.


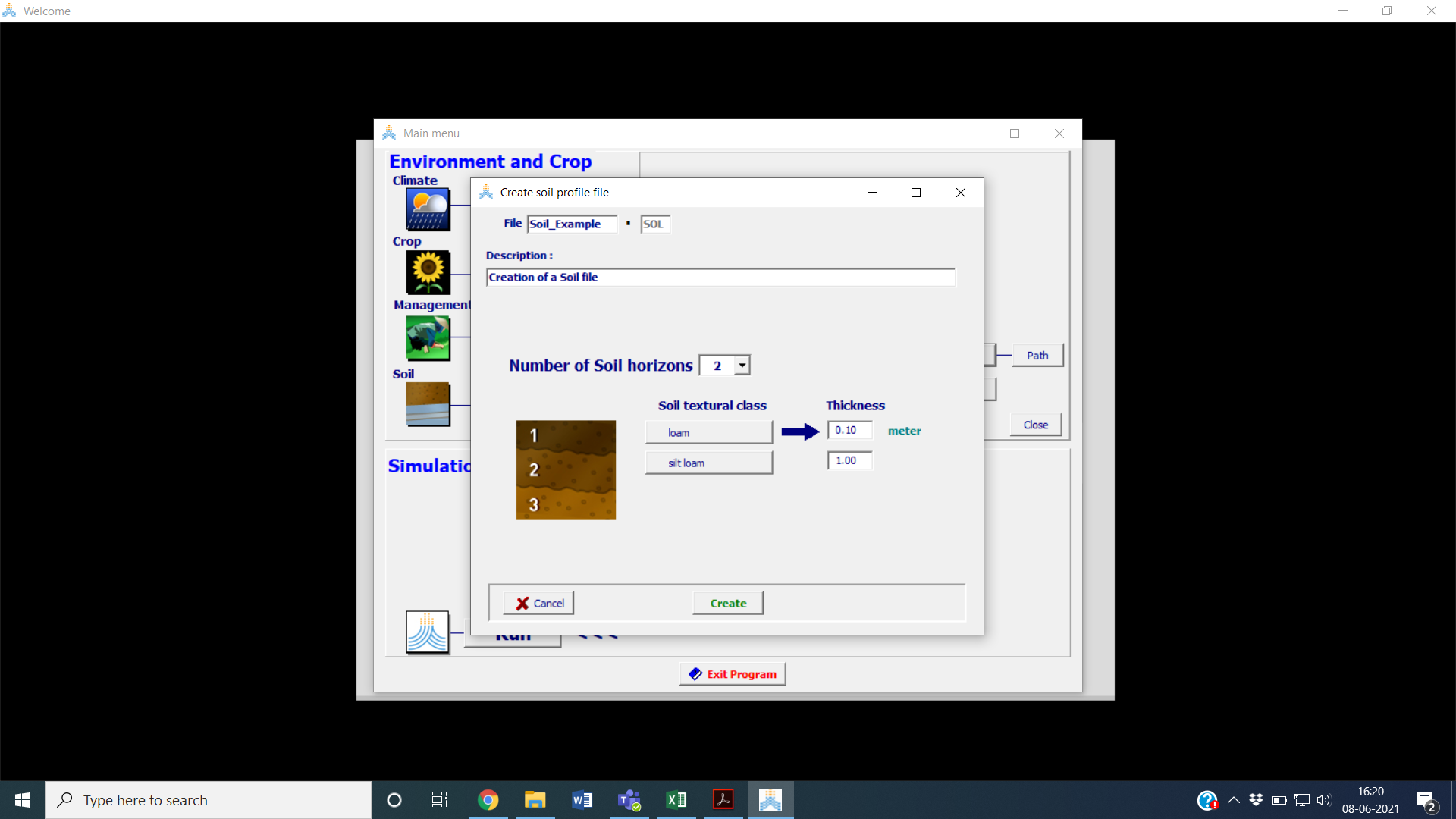


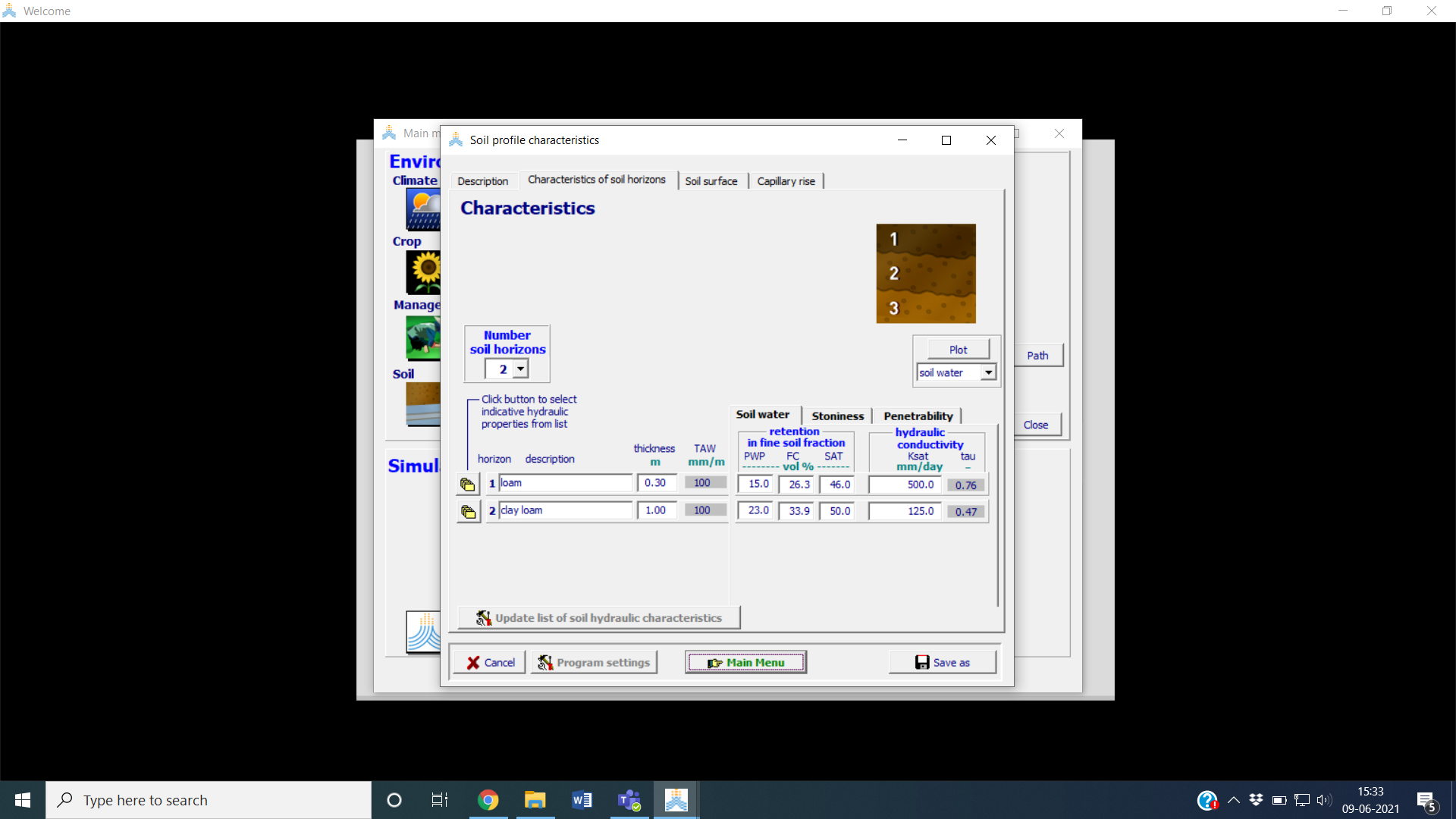


This process must be repeated 26 times.

1. Now, the output for all soil types must be calculated. For simplicity, the climate around Palmyra will be used for all calculations. While we will lose some precision, the city is at the centre of all Cost envelopes, meaning that the overestimation of outputs in drier areas will be compensated by the underestimation of outputs in larger areas. The simulation must be iterated, changing the soil type each time.

- Climate: Climate_Palmyra.CLI
- Crop: BarleyGDD.CRO
- Irrigation: none
- Field: Management_Palmyra.MAN
- Soil: Each of the 26 soil types
- Groundwater: none
- Simulation Period: From 1 November to 31 March
- Initial conditions: none
- Project: none
- Field data: none


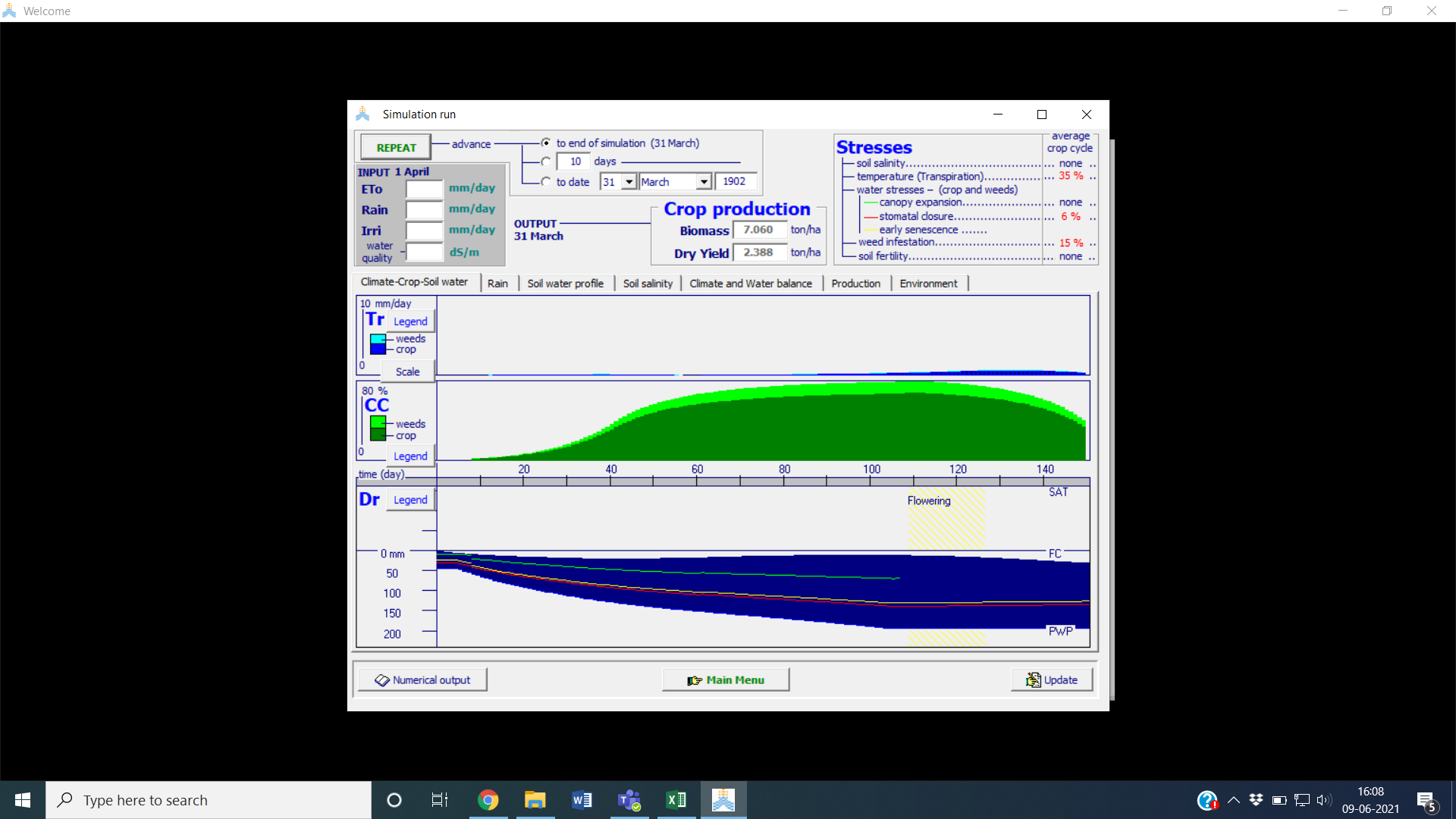


1. Finally, the output for the four areas suitable for irrigation must be established. The outputs in tons/ha must be calculated under irrigation, at a level of 200 mm (125 mm of rainfall + 75 if of irrigation) and of 400 mm (125 mm of rainfall + 275 mm of irrigation).

For these areas, the outputs will be calculated taking into account their local climate. In the case of At-Tarqah and the Oasis, both will use the climate data from around Palmyra.

- Climate: Climate_X.CLI
- Crop: BarleyGDD.CRO
- Irrigation: Either Irrigation_200.IRR or Irrigation_400.IRR
- Field: Management_Palmyra.MAN
- Soil: Each of the 9 soil types concerned (5 for 3322 and 4 for 3324)
- Groundwater: none
- Simulation Period: From 1 November to 31 March
- Initial conditions: none
- Project: none
- Field data: none


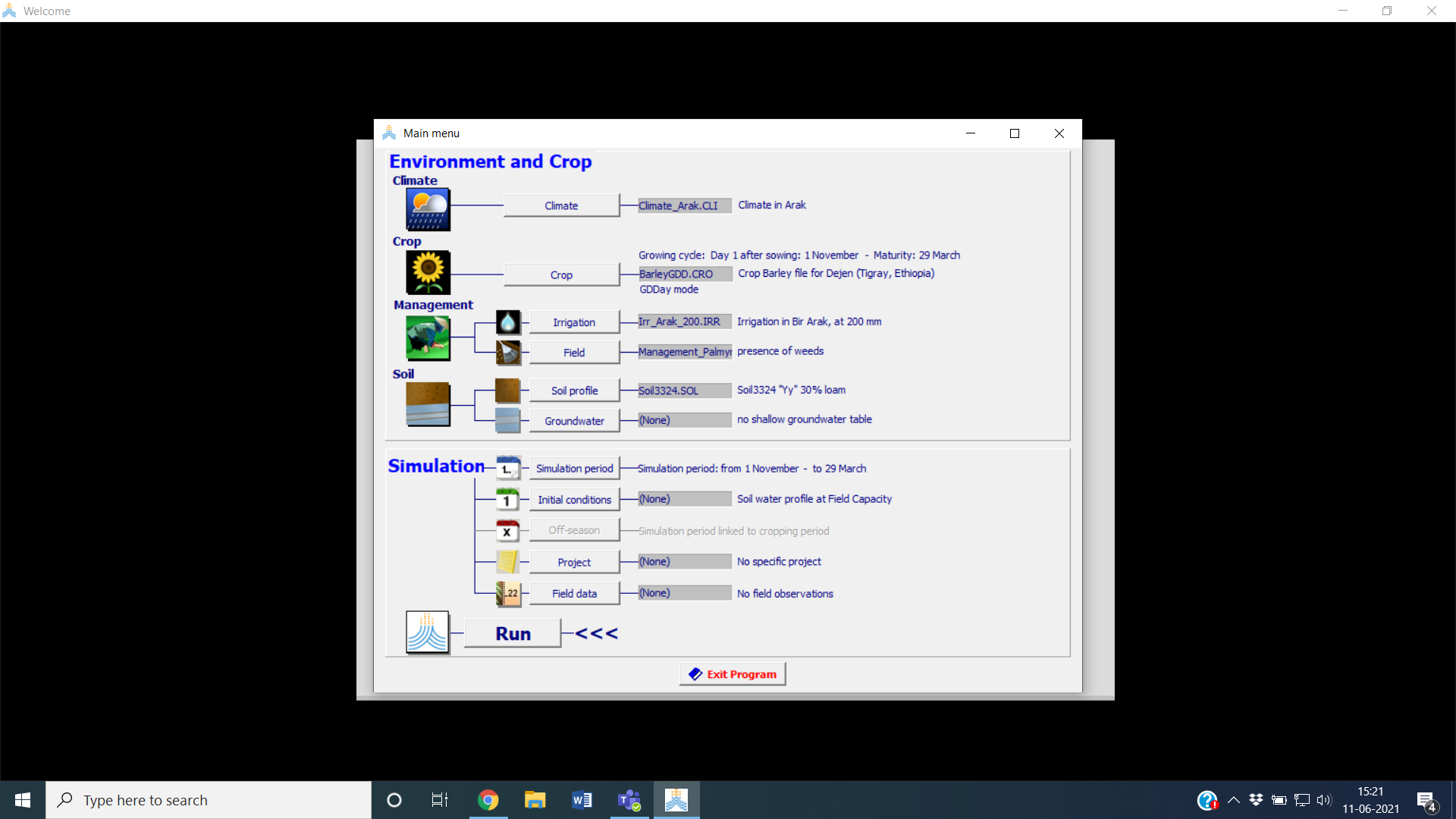


1. **Steps in Excel 2016**


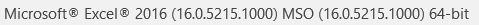


1. At first, a rough optimistic estimate for the productivity of the different soil areas, based on estimated rainfall, will be produced. This will be based on a contemporary australian ratio of 12 kg/ha/mm (10,11), and the assumption of a 1 year fallow:

**Table:** Rough outputs based on Rainfall

**Data inputs:** Yearly_precipitation.tif, Rainfall.shp, Soil_Far.shp, Soil_Medium.shp, Soil_Near.shp

**Columns:**

- ID: Assign each Soil Area an ID number just in case
- Z: Copied from QGIS (Soil_X.shp, attribute “Z”)
- Area: Copied from QGIS (Soil_X.shp, attribute “Area”)
- Rainfall: Manually introduce the number between 100, 150, 200, and 250 that more closely matches the annual precipitation in each given area.
- Area ha: Area/10000
- Output: (Rainfall*Area ha*12)/2
- People Fed Max: Output/360
- People Fed Min: Output/510

**Summary:** ∑ People Fed Max, ∑People Fed Min


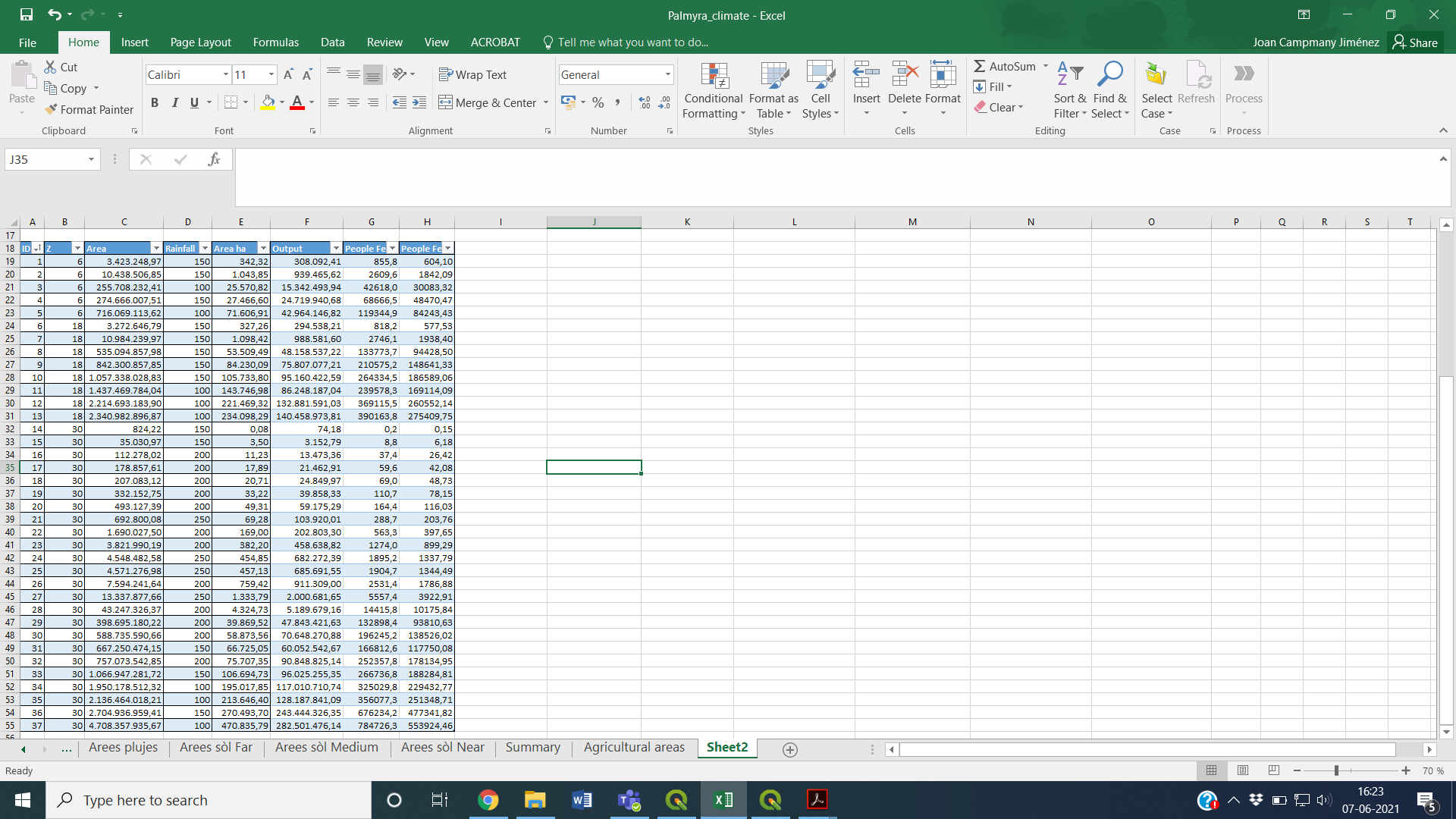


This table needs to be repeated for each of the Soil shapefiles (Soil_Far.shp, Soil_Medium.shp, Soil_Near.shp), or either merged in a single table, but in that case the different Cost envelopes (“Z”= 6, 18 and 30) have to be added up separately. The summaries can be expressed as a single table.

**Table A**

| **Area** | **Output (kg)** | **People Fed Max** | **People Fed Min** |
| --- | --- | --- | --- |
| **Far** | 1.811.231.760,89 | 5.031.199,34 | 3.551.434,83 |
| **Medium** | 664.272.048,18 | 1.845.200,13 | 1.302.494,21 |
| **Near** | 84.274.139,46 | 234.094,83 | 165.243,41 |

Rough outputs based on simple rainfall ratios and WorldClim modern yearly averages.

1. A more precise estimate can be obtained by using AquaCrop and simulating the output for each of the soil areas. This will be better done in three separate tables, one for each cost envelope.

**Table:** Outputs based on AquaCrop

**Data inputs:** Soil_Far.shp, Soil_Medium.shp, Soil_Near.shp, AquaCrop

**Columns:**

- MU_GLOBAL: Copied from QGIS (Soil_X.shp, attribute “MU_GLOBAL”)
- Share: Copied from QGIS (Soil_X.shp, attribute “Share”)
- SEQ: Copied from QGIS (Soil_X.shp, attribute “SEQ”)
- REF_DEPTH: Copied from QGIS (Soil_X.shp, attribute “REF_DEPTH”)
- AWC_CLASS: Copied from QGIS (Area_X.shp, attribute “AWC_CLASS”)
- T_GRAVEL: Copied from QGIS (Area_X.shp, attribute “T_GRAVEL”)
- T_USDA_TEX: Copied from QGIS (Area_X.shp, attribute “T_USDA_TEX”)
- S_GRAVEL: Copied from QGIS (Area_X.shp, attribute “S_GRAVEL”)
- S_USDA_TEX: Copied from QGIS (Area_X.shp, attribute “S_USDA_TEX”)
- Area: Copied from QGIS (Soil_X.shp, attribute “Area”)
- Area ha: Area/10000
- Output tons/ha: Introduce manually the Dry Yields simulated in AquaCrop.
- Area concerned: Area ha*Share/100
- Output kg: (Output tons/ha*Area concerned*1000)/2
- People Fed Max: Output/360
- People Fed Min: Output/510

**Summary:** ∑ People Fed Max, ∑People Fed Min


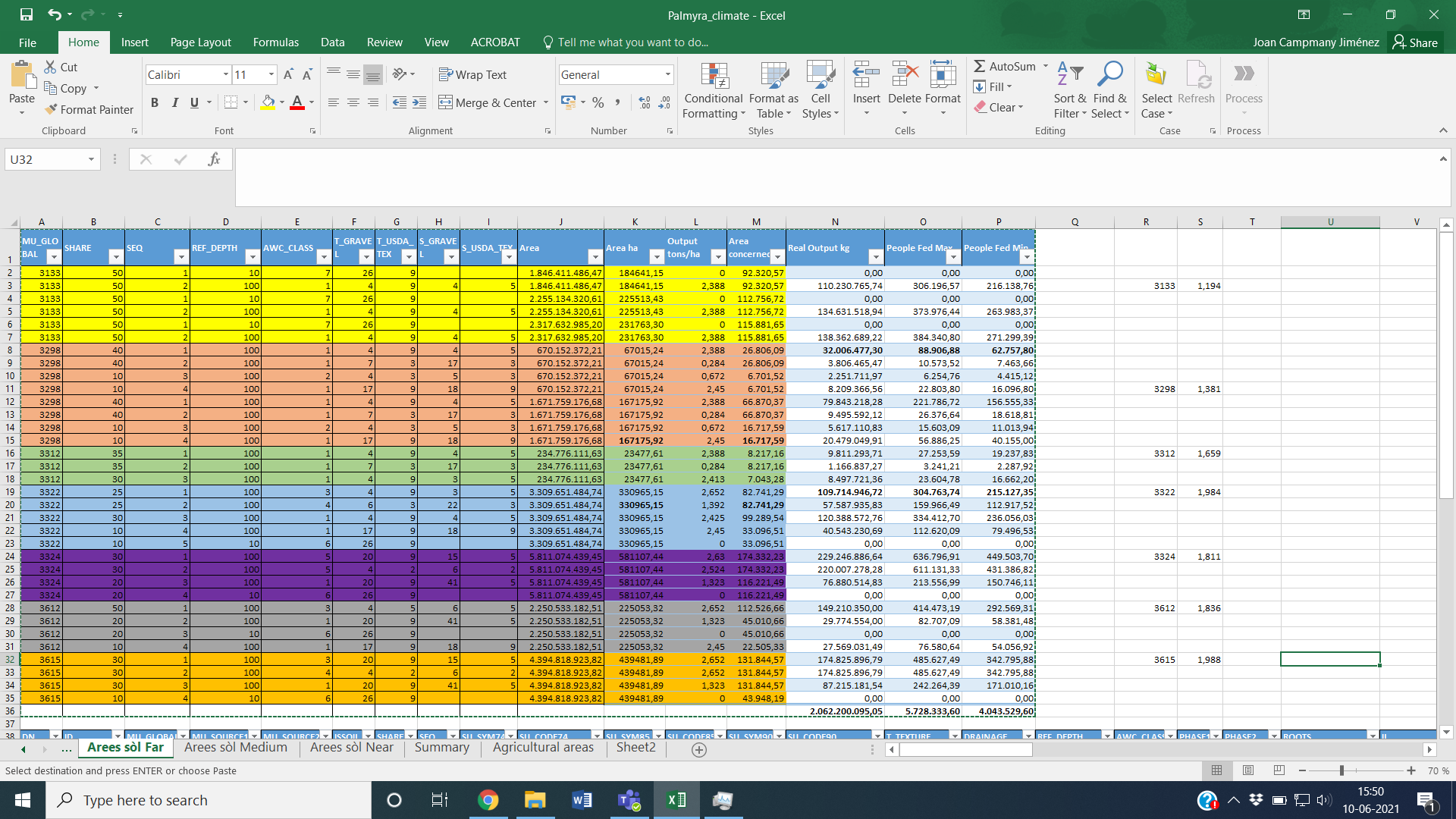


The process needs to be repeated for each of the Soil shapefiles (Soil_Far.shp, Soil_Medium.shp, Soil_Near.shp). The results can be summed up in a single table.

**Table B**

| **Areas** | **Barley output (kg)** | **Fallow Max** | **Fallow Min** |
| --- | --- | --- | --- |
| **Far** | 2.062.200.095,05 | 5.728.333,60 | 4.043.529,60 |
| **Medium** | 843.072.106,96 | 2.341.866,96 | 1.653.082,56 |
| **Near** | 109.085.280,51 | 303.014,67 | 213.892,71 |

Precise outputs based on AquaCrop.

1. Now, the outputs of the different farming areas around Palmyra will be calculated. First, for simplicity, the weighted average productivity of the soil areas concerned will be calculated. This can easily be done with a table.

**Table:** Weighted average of outputs

**Data inputs:** Soil_Far.shp, AquaCrop

**Columns:**

- Area: The name of each of the 4 farming areas
- Soil Type: Copied from QGIS (Soil_Far.shp, attribute “SEQ”)
- Share: Copied from QGIS (Soil_Far.shp, attribute “Share”)
- Output 400: Introduce manually the Dry Yields simulated in AquaCrop for 400 mm of irrigation.
- Output 200: Introduce manually the Dry Yields simulated in AquaCrop for 200 mm of irrigation.

**Summary:** ∑Area(Output 400*Share)/100, ∑Area(Output 200*Share)/100


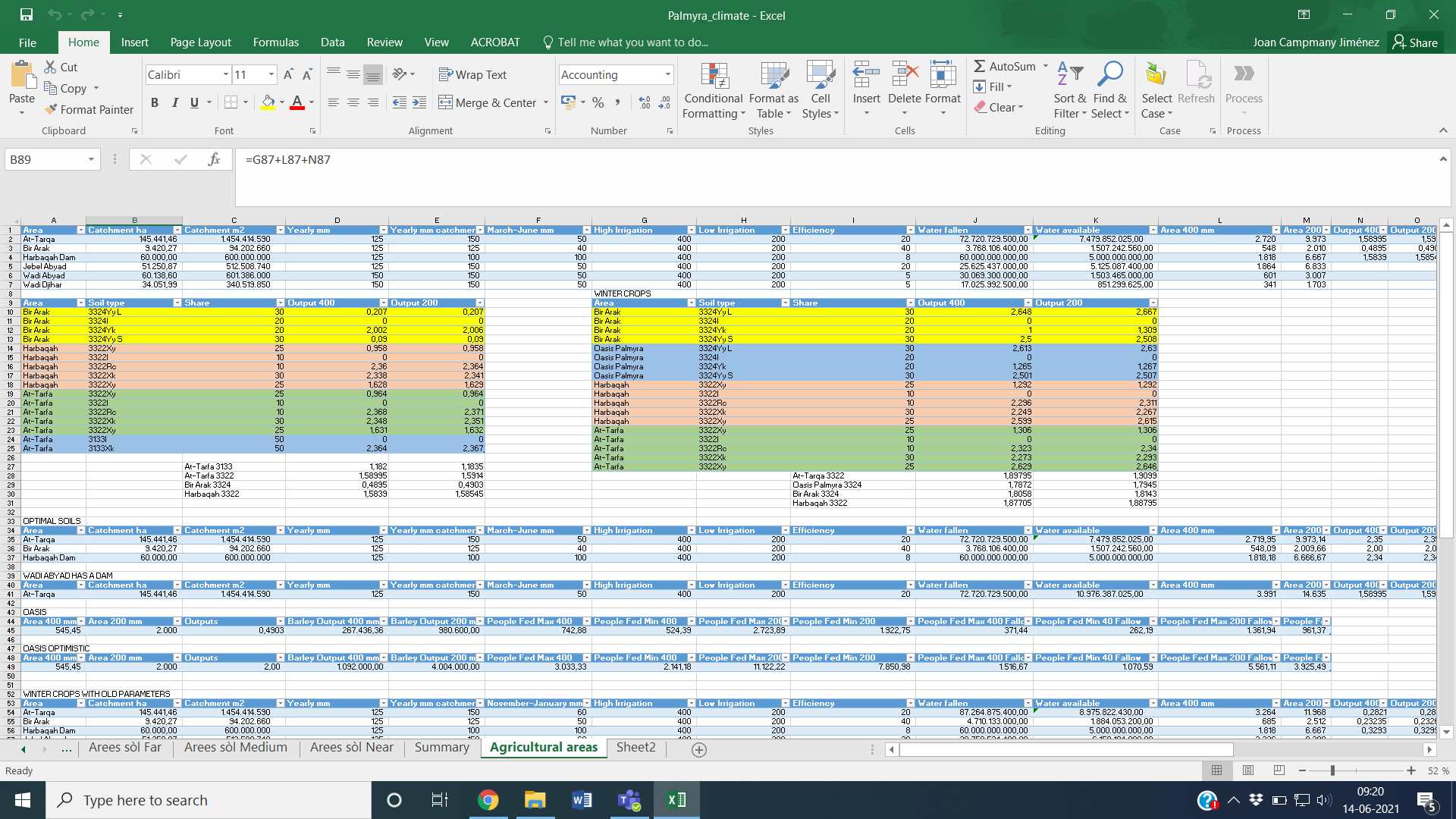


**Table C**

| Area | Soil units | Weighted productivity at 400 mm (tonnes/ha) | Weighted productivity at 200 mm (tonnes/ha) |
| --- | --- | --- | --- |
| **At-Tarqa** | 3322 | 1,89795 | 1,9099 |
| **Oasis Palmyra** | 3324 | 1,7872 | 1,7945 |
| **Bir Arak** | 3324 | 1,8058 | 1,8143 |
| **Harbaqah** | 3322 | 1,87705 | 1,88795 |

Weighted average productivity for each agricultural area.

1. Now the maximum irrigated areas will be calculated from the monthly 30-year averages we collected in step 1.11. Due to the differences between areas, they will be calculated separately.

*For At-Tarqah:*


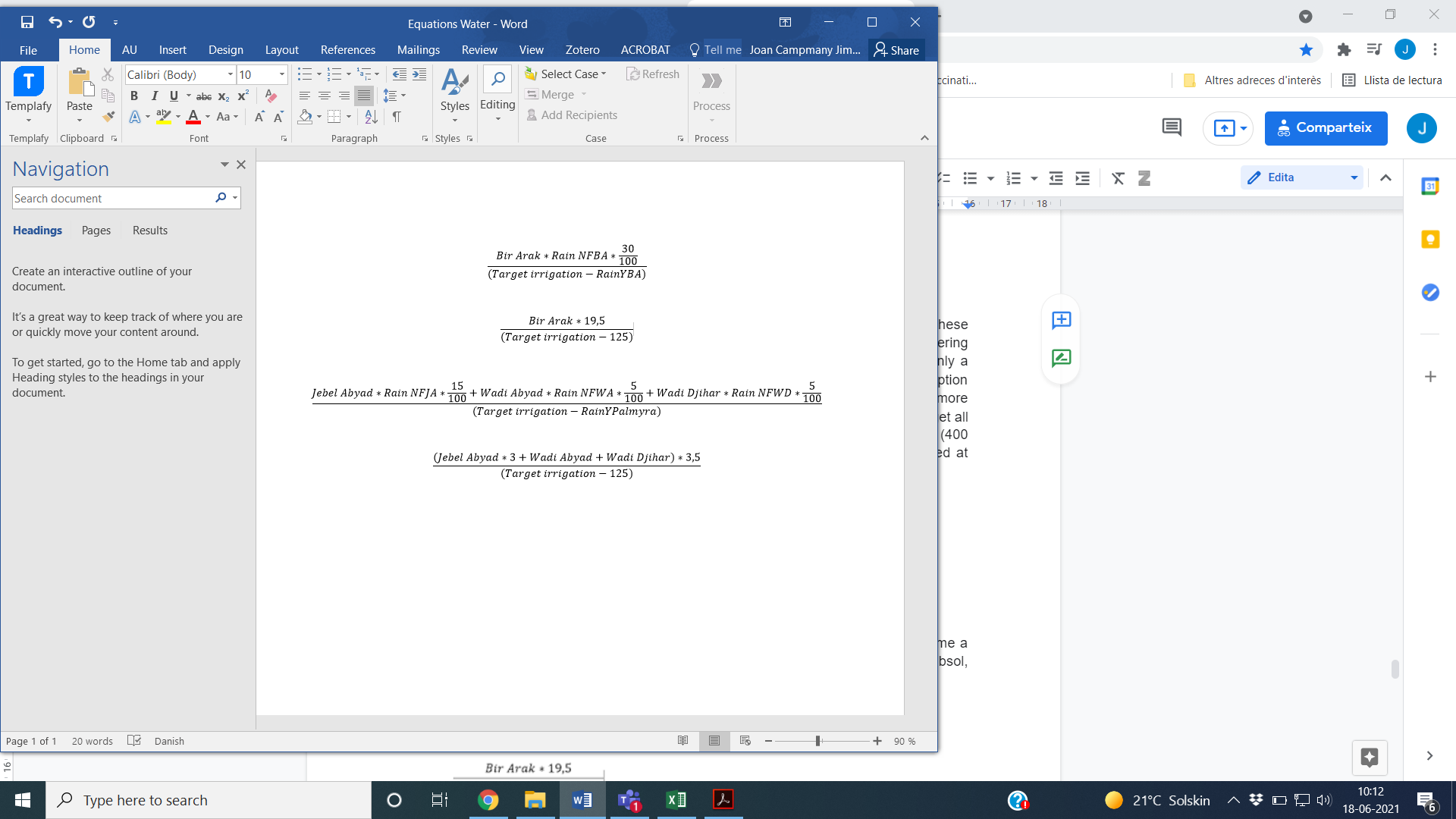


Where “Jebel Abyad”, “Wadi Abyad” and “Wadi Djihar” are the areas for these catchments in ha. By multiplying by the available rainfall in each area Between November and February (Rain NFXX), we get an approximate number of the total rainfall in liters. Only a portion of this water can reach the fields (evaporation, uncontrolled runoff, absorption by the soil, pooling). We estimate 15% for Jebel Abyad, and only 5% for the more distant Wadis. The total available water for irrigation then needs to be divided by the water necessary for irrigation (target irrigation minus rainfall). We then get the ha that could be irrigated at either level.

*For Bir Arak:*


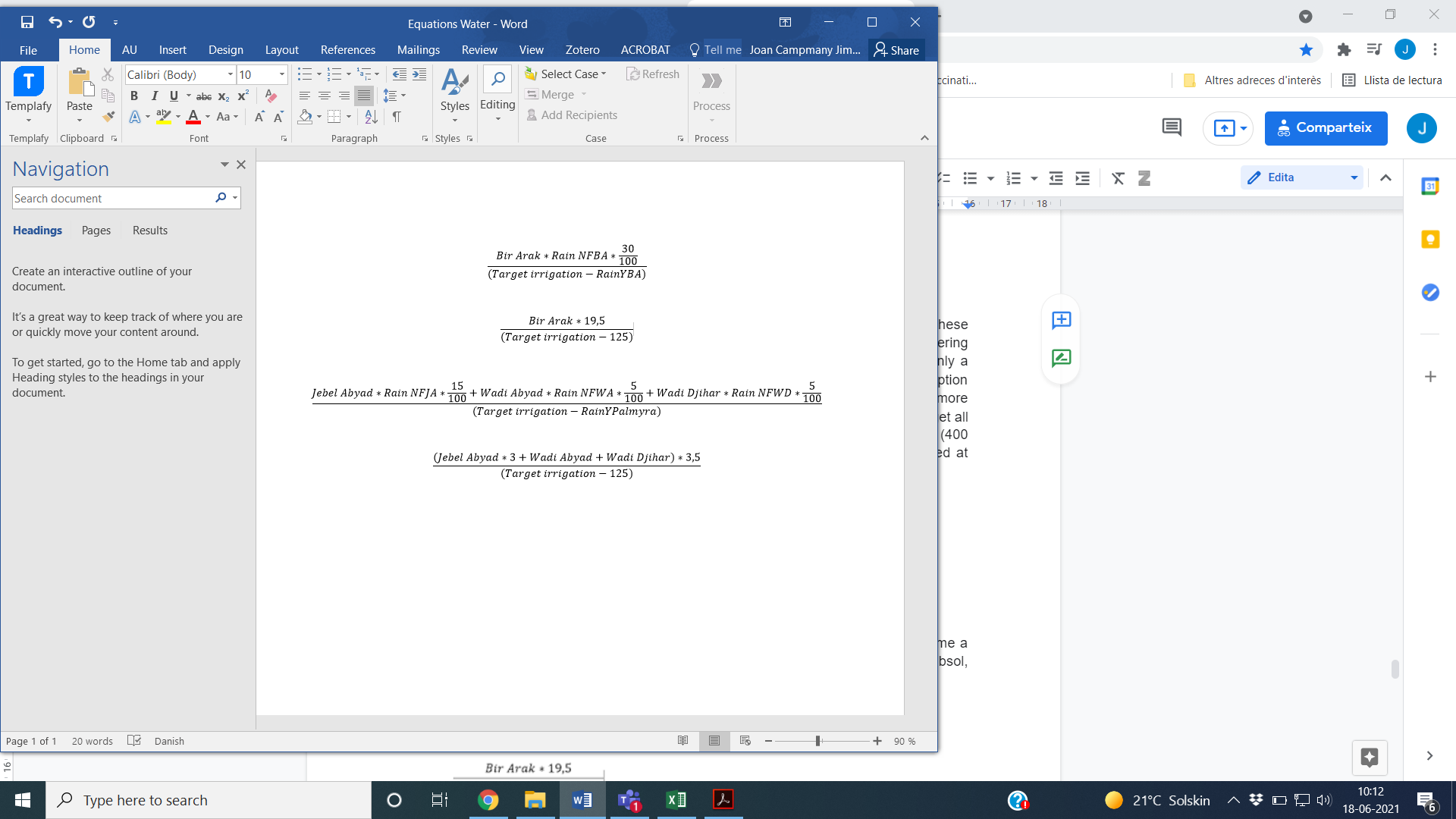


This is the same equation, but with just one catchment. In this case, we assume a much higher efficiency because of the presence of Qanats.

*For Harbaqah:*

Here the water availability comes from the capacity of the Dam. Several sources (12–16) estimate a capacity of at least 5000000 cubic meters. The equation is much more simple:


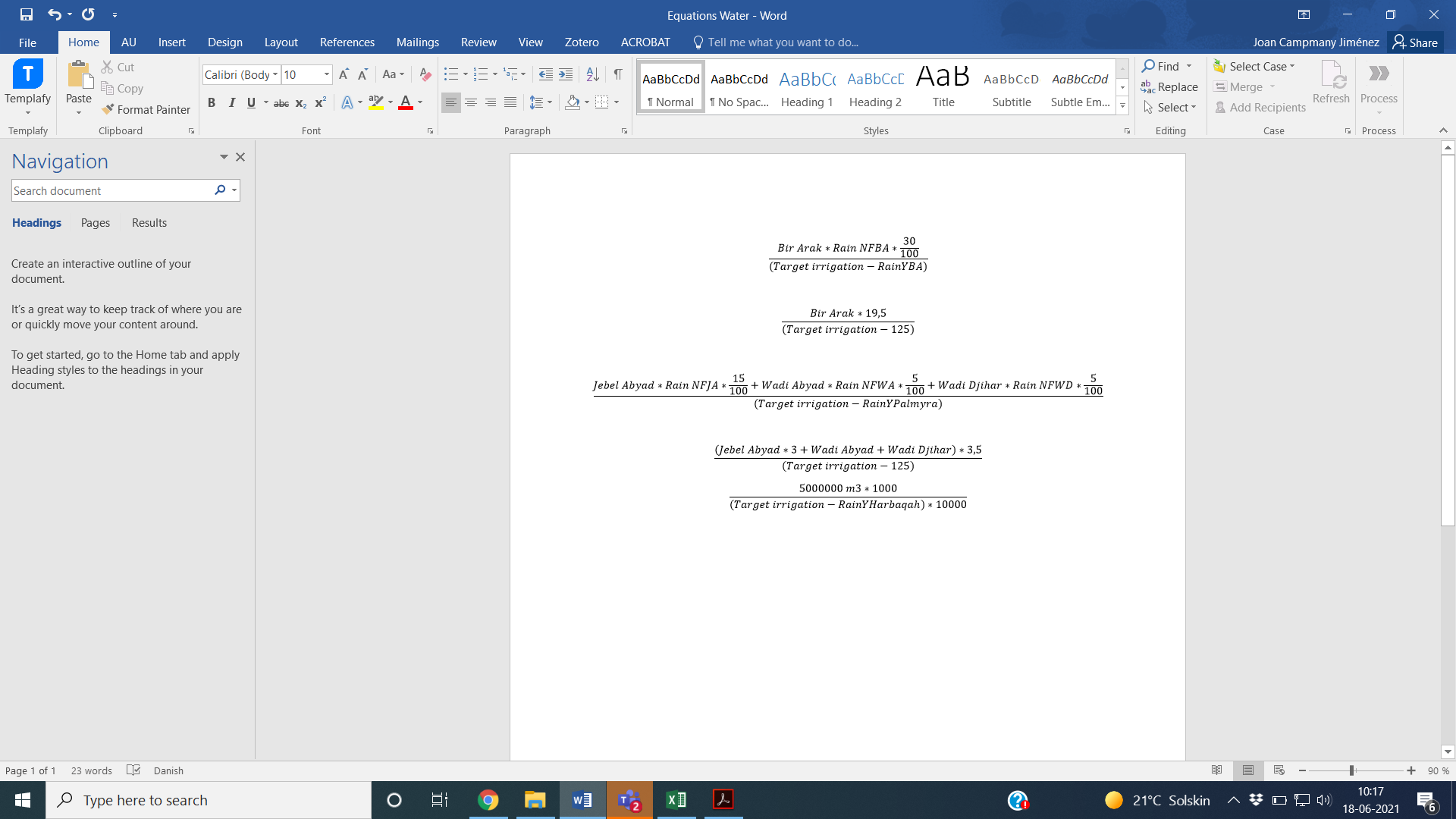


*For the Oasis*:

It is really complicated to establish how much water would have been available for irrigation in the Oasis. For our purposes, we will assume that the entire area (twice as much as is irrigated today) could be irrigated at 200 mm.

For 400 mm:


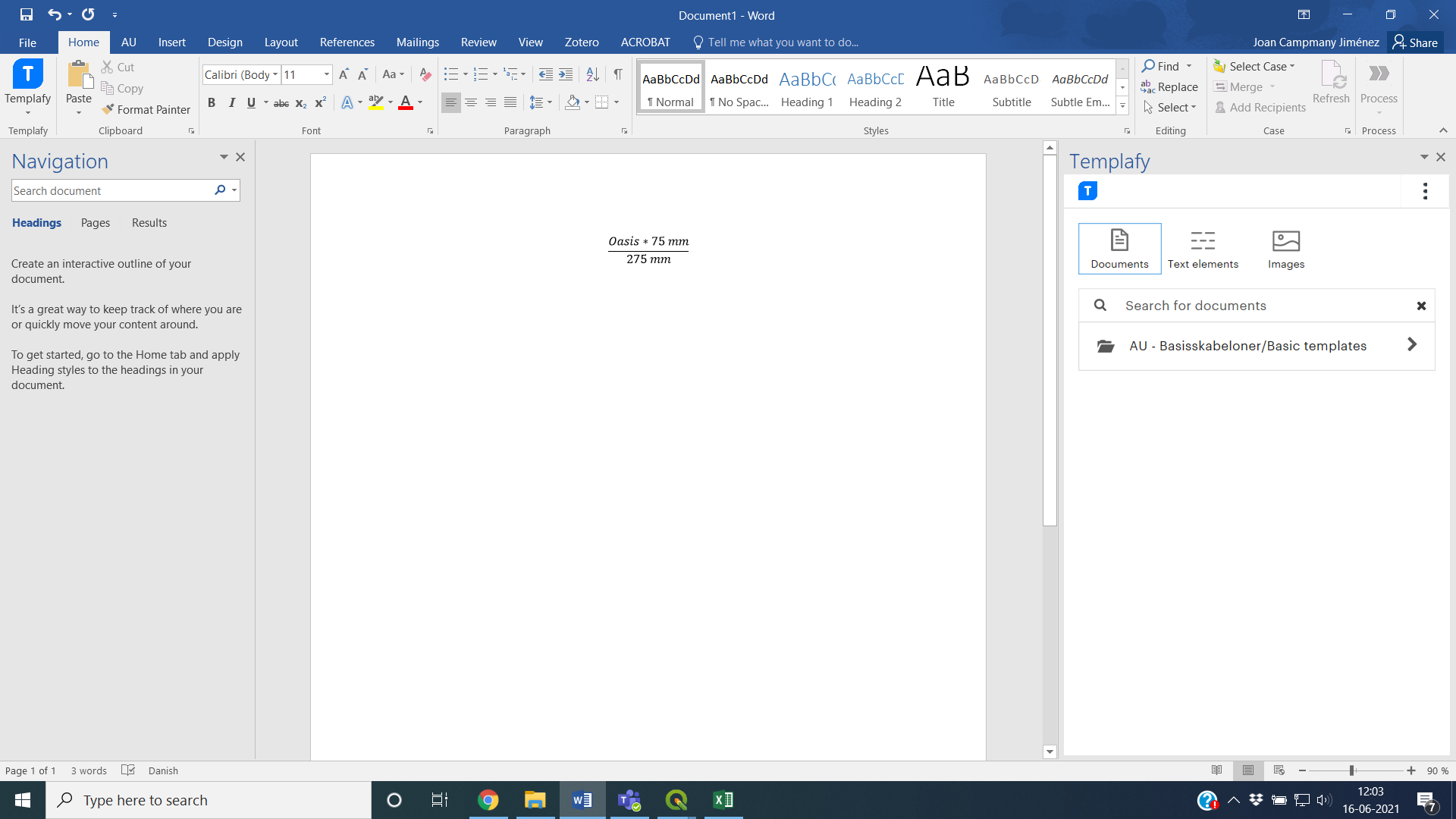


These are our numbers, in ha, with the rainfalls given by the WorldClim 30-year monthly average rasters:


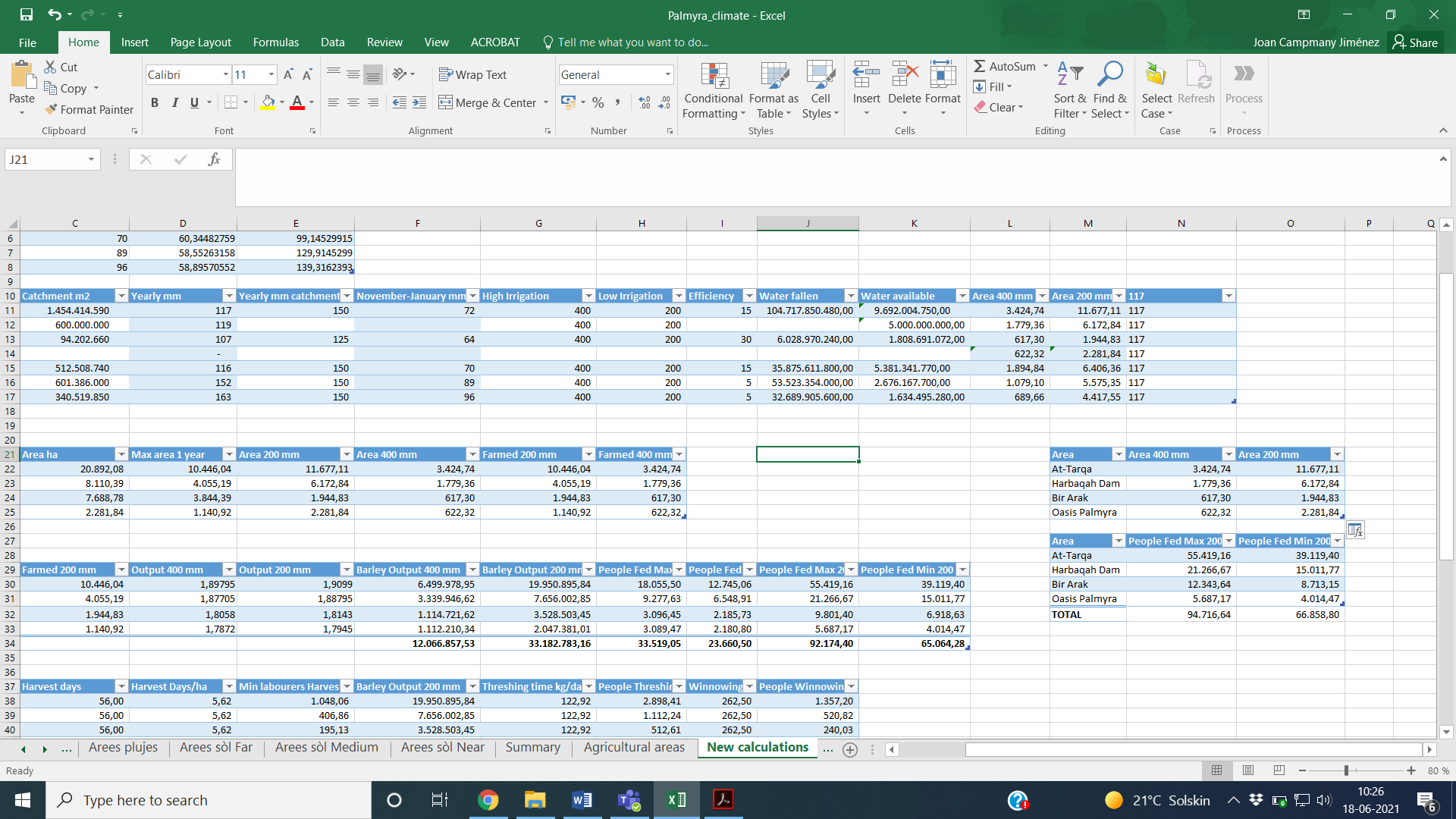


**Table D**

| Farming areas | Area irrigated at 200 mm (ha) | Area irrigated at 400 mm (ha) |
| --- | --- | --- |
| At-Tarqa | 11.677,11 | 3.424,74 |
| Harbaqah Dam | 6.172,84 | 1.779,36 |
| Bir Arak | 1.944,83 | 617,30 |
| Oasis Palmyra | 2.281,84 | 622,32 |

The area that could be irrigated with WorldClim modern yearly averages.

1. The area that could be farmed every year comes determined by two factors. First, how much could be irrigated, fallow. That means that for any given area, only half of the land could be farmed in a year, and that only if there was enough water to irrigate it.

**Table:** Areas farmed yearly

**Data Inputs:** Areas for farming areas measured roughly in QGIS, areas calculated in step 3.4

**Columns:**

- Farming area: The name of each of the 4 farming areas
- Area: Measured in QGIS (Step 1.13)
- Area ha: Area/10000
- Max area 1 year: Area ha/2
- Area 200mm: Calculated in Step 3.4
- Area 400mm: Calculated in Step 3.4
- Farmed 200mm: MIN(Max area 1 year; Area 200mm)
- Farmed 400mm: MIN(Max area 1 year; Area 400mm)

**Summary:** Farmed 200mm; Farmed 400mm

These are our numbers:


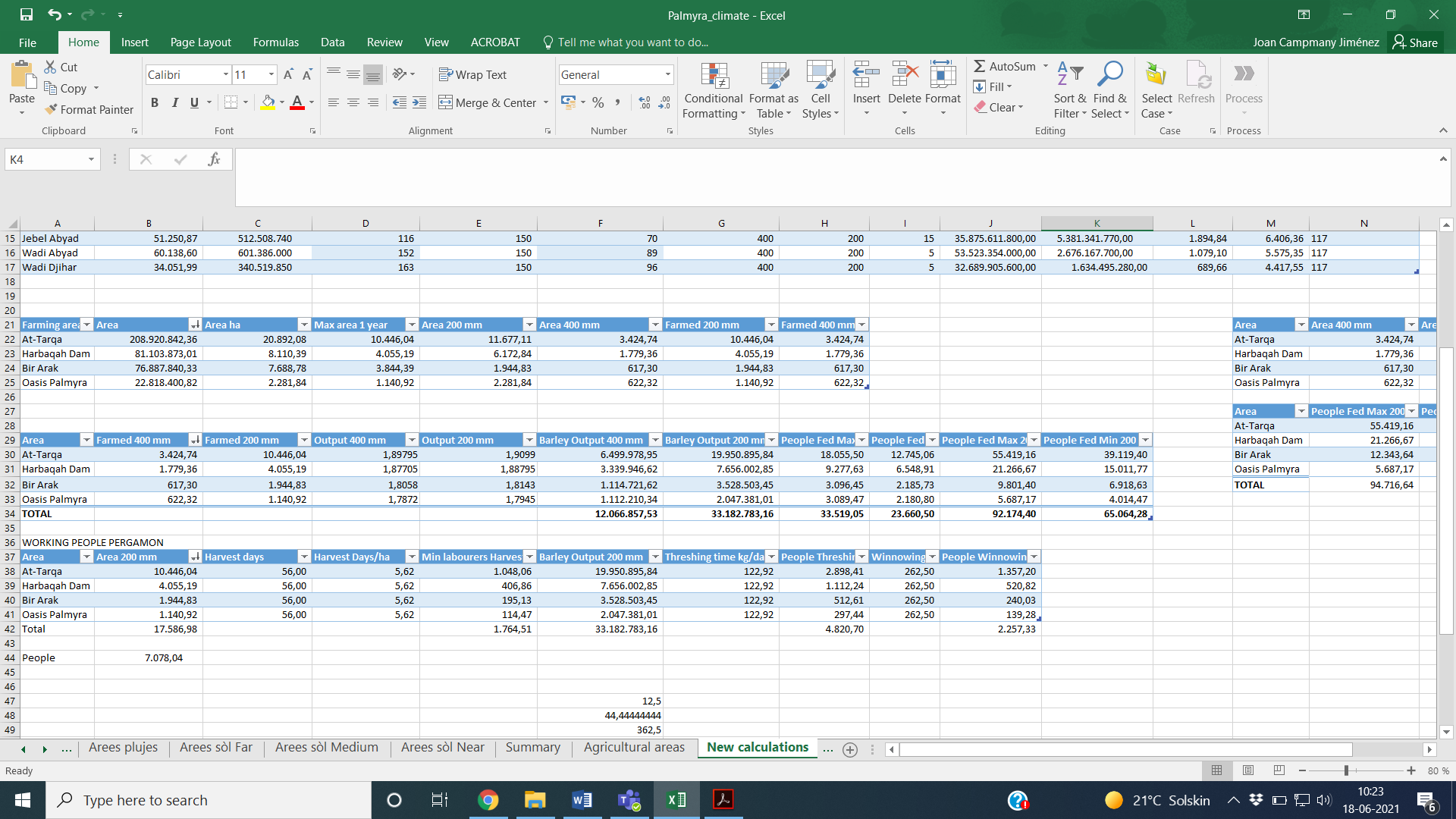


**Table E**

| Farming areas | Farmed area 200 mm (ha) | Farmed area 400 mm (ha) |
| --- | --- | --- |
| At-Tarqa | 10.446,04 | 3.424,74 |
| Harbaqah Dam | 4.055,19 | 1.779,36 |
| Bir Arak | 1.944,83 | 617,30 |
| Oasis Palmyra | 1.140,92 | 622,32 |

Areas that could be farmed with WorldClim modern yearly averages.

1. Now we can calculate the output for each area of Irrigated agriculture.

**Table:** Outputs based on AquaCrop for farming areas.

**Data inputs:** AquaCrop, Calculations in step 3.3 and 3.5.

**Columns:**

- Farming area: Name of each of the 4 areas.
- Farmed 400 mm: Calculated in step 3.5.
- Farmed 200 mm: Calculated in step 3.5.
- Output 400: Calculated in step 3.3.
- Output 200: Calculated in step 3.3.
- Barley output 400: Area 400*Output 400*1000
- Barley output 200: Area 200*Output 200*1000
- People Fed Max 400: Barley output 400/360
- People Fed Min 400: Barley output 400/510
- People Fed Max 200: Barley output 200/360
- People Fed Min 200: Barley output 200/510

**Summary:** ∑ People Fed Max 200, ∑People Fed Min 200


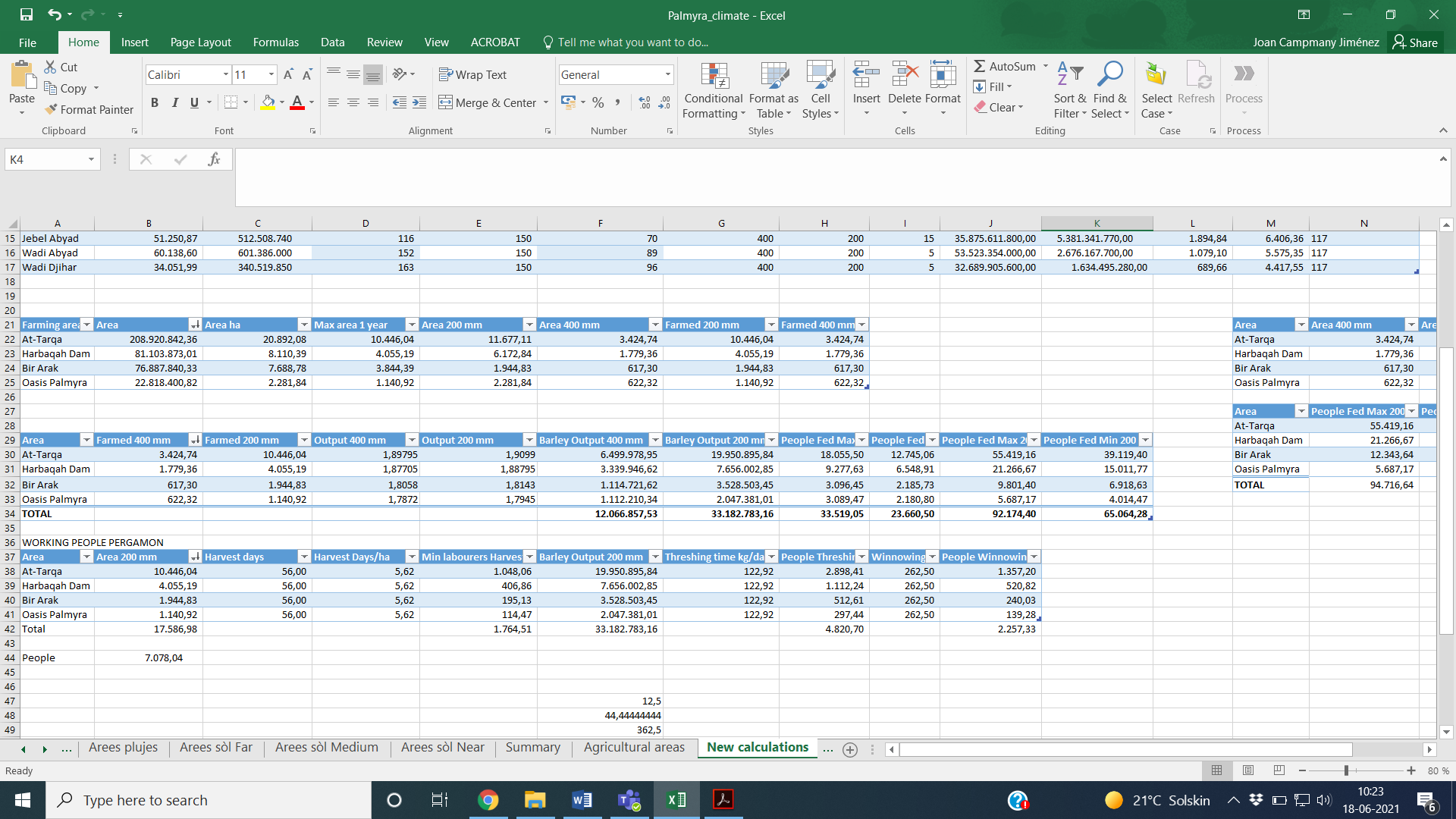


The results are as follows

**Table F**

| **Area** | **People Fed Max 400** | **People Fed Min 400** | **People Fed Max 200** | **People Fed Min 200** |
| --- | --- | --- | --- | --- |
| **At-Tarqa** | 18.055,50 | 12.745,06 | 55.419,16 | 39.119,40 |
| **Harbaqah Dam** | 9.277,63 | 6.548,91 | 21.266,67 | 15.011,77 |
| **Bir Arak** | 3.096,45 | 2.185,73 | 9.801,40 | 6.918,63 |
| **Oasis Palmyra** | 3.089,47 | 2.180,80 | 5.687,17 | 4.014,47 |
| **TOTAL** | 33.519,05 | 23.660,50 | 92.174,40 | 65.064,28 |

People fed, based on AquaCrop productivity estimates, for each farming area. Two irrigation levels were calculated: 200 and 400 mm.

The results make it clear that, for Barley, 200 mm of irrigation are ideal. 400 mm make sense for other types of crops, but not for Barley. Since fallow was practiced, these are our numbers:


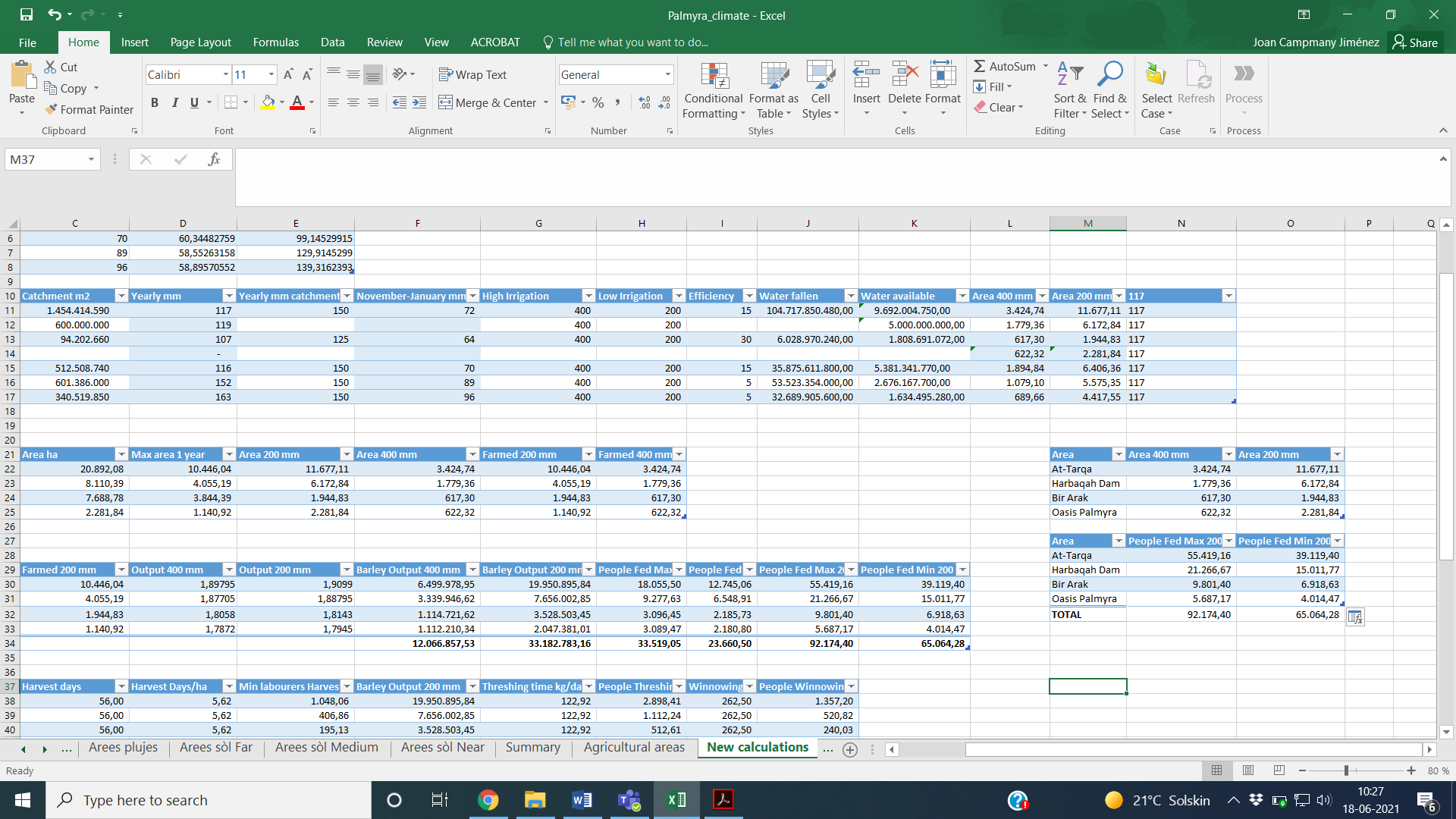


1. Now, we will calculate the amount of people required to harvest the farmed areas. we will use numbers by Laabs and Knitter (17) and the Cologne Tableau (18,19), averaged as shown in the document “workforce.xslx”.

**Table:** Workforce requirements for harvest.

**Data inputs:** Calculations in steps 3.5 and 3.6.

**Columns:**

- Farming area: Name of each of the 4 areas.
- Area 200 mm: Calculated in step 5, same as in step 6.
- Harvest days: 56
- Days/ha: 5,68
- Labourers harvest: (Area 200 mm*”Days/ha”/Harvest days)
- Barley output 200 mm: Calculated in step 3.6.
- Threshing kg/day: 122,92
- People Threshing: (Barley output 200 mm/”Threshing kg/day”)/Harvest days
- Winnowing kg/day: 262,50
- People Winnowing: (Barley Output 200 mm/”Winnowing kg/day”)/Harvest days

**Summary:** ∑ Labourers harvest + ∑People Threshing + ∑People Winnowing.


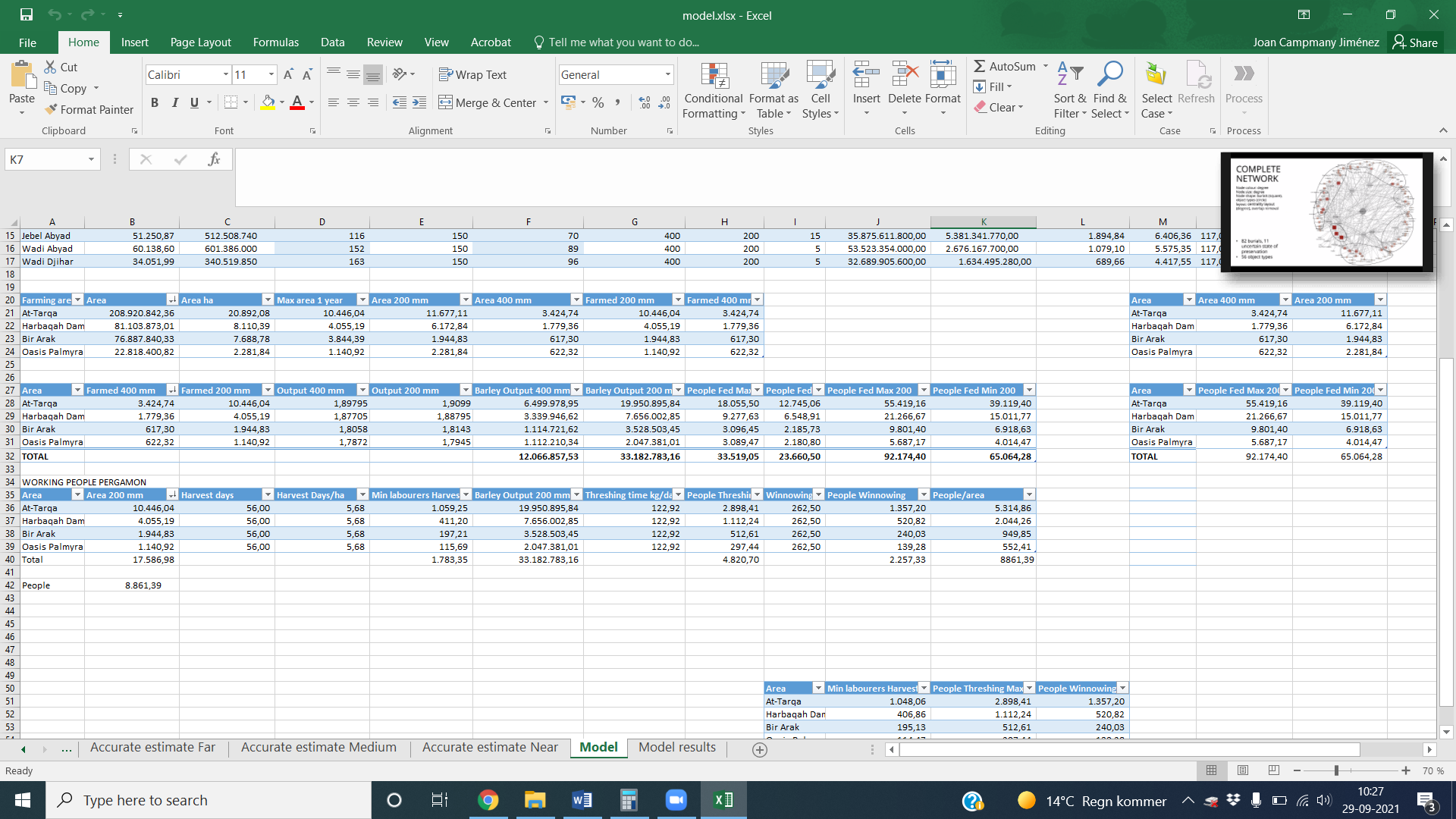


The numbers of workers necessary for each of the tasks in the harvest season must be put together.

| Workers | 8.861,39 |
| --- | --- |

1. For all of our calculations so far we have employed 30-year monthly averages from WorldClim, meaning that our numbers are the average for the present day. However, we have access to Yearly rainfall for Palmyra going back to the 30s and 50s (20). To see possible outputs with different rainfall levels, we must be able to convert Palmyrene yearly rainfalls into the yearly values for all the catchments and farming areas, and in turn, to convert those yearly totals into growing period rainfalls.

The most simple way to do this is by establishing the mathematical proportions between all these values and Palmyrene yearly rainfalls.

**Table:** Rainfall estimates in relation to Palmyrene Yearly rainfalls.

**Data inputs:** Rainfall data collected in step 1.11.

**Columns:**

- Rainfall area: Name of each of the 4 farming areas (the Oasis is not necessary, and can be left blank).
- Yearly rainfall: ∑monthly values collected in step 1.11.
- Nov-Feb: ∑of November, December, January and February rainfalls collected in step 1.11.
- %: Yearly rainfall/Nov-Feb*100
- %Palmyra: Yearly rainfall/117*100

**
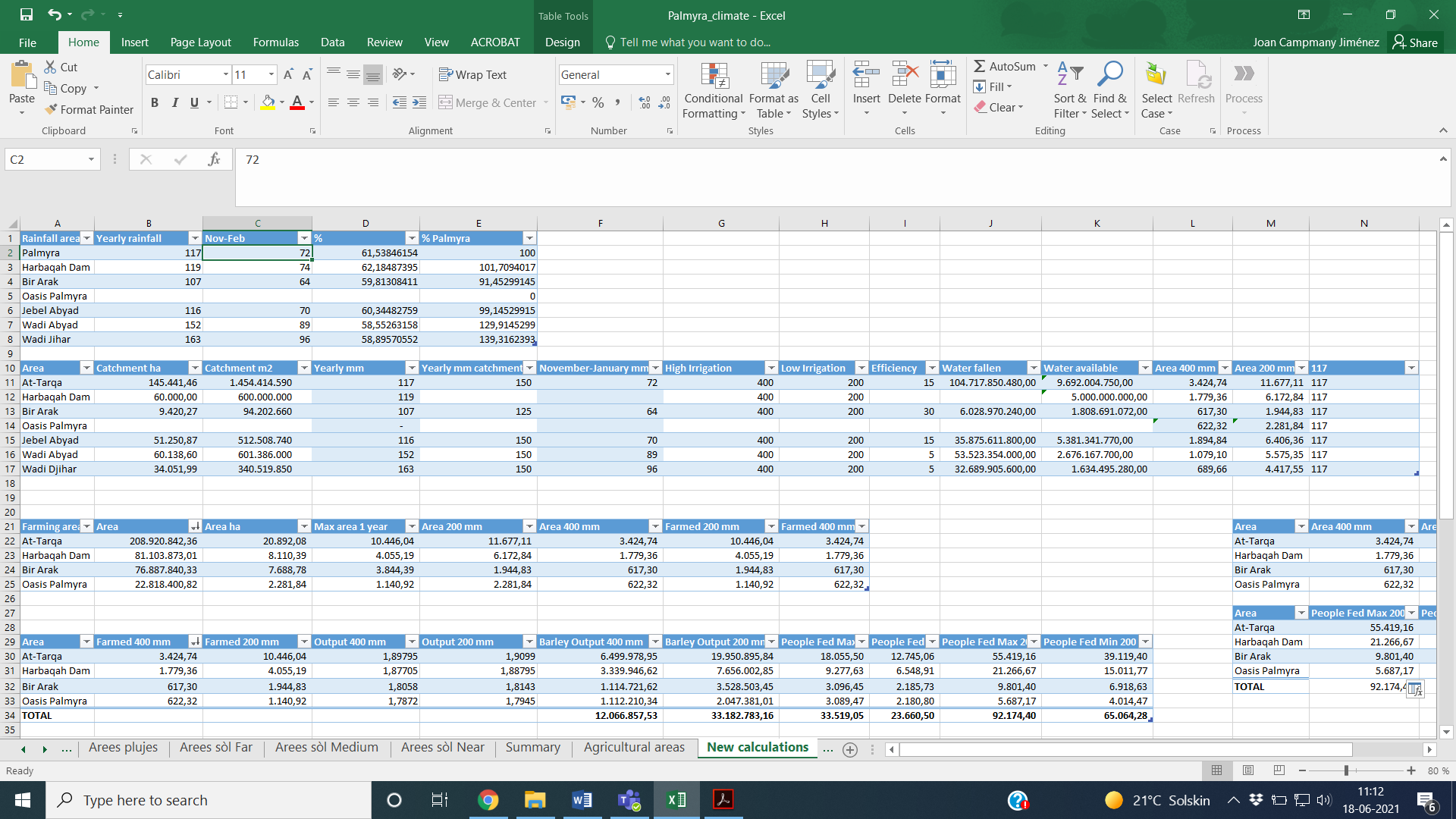
**

**Table G**

| Watershed | Yearly rainfall (mm) | Growing period (November-February) rainfall (mm) | % | % of Palmyrene rainfall |
| --- | --- | --- | --- | --- |
| Palmyra | 117 | 72 | 61,54 | 100,00 |
| Harbaqah Dam | 119 | 74 | 62,18 | 101,71 |
| Bir Arak | 107 | 64 | 59,81 | 91,45 |
| Oasis Palmyra |  |  |  | 0,00 |
| Jebel Abyad | 116 | 70 | 60,34 | 99,15 |
| Wadi Abyad | 152 | 89 | 58,55 | 129,91 |
| Wadi Jihar | 163 | 96 | 58,90 | 139,32 |

Proportion of growing period rainfalls to yearly rainfalls and proportion of yearly rainfalls to Palmyrene rainfall. The Oasis of Palmyra can be ignored, since we do not have information about its irrigation and have not modelled it.

With these mathematical relations, any rainfall value we use in our model can be calculated from the Yearly Palmyrene total rainfall, and introduced in the model to test outputs at different rainfalls.

Values

| ***Step*** | ***Process*** | ***Parameter*** | ***Value*** |
| --- | --- | --- | --- |
| **2.2** | Creating the irrigation files | Irrigation method | Surface Irrigation - Border Irrigation |
| **2.2** | Creating the irrigation files | Percentage of soil surface wetted | 100% |
| **2.2** | Creating the irrigation files | Valid from | Day. No. 1 |
| **2.2** | Creating the irrigation files | Time Criteria>When? | (Interval:93 days) |
| **2.2** | Creating the irrigation files | Fixed net application | 75/275 mm |
| **2.2** | Creating the irrigation files | Water quality | Excellent |
| **2.3** | Creating the soil management files | Soil fertility | non limiting, 100% |
| **2.3** | Creating the soil management files | Soil surface cover | none, 0% |
| **2.3** | Creating the soil management files | Field surface practices, practices preventing runoff | none |
| **2.3** | Creating the soil management files | Soil bunds | none |
| **2.3** | Creating the soil management files | Weed management | good (relative cover of weeds 15%) |
| **2.5 and 2.6** | Simulating Outputs in tons/ha | Simulation Period | From 1 November to 31 March |
| **3.1, 3.2, and 3.6** | Calculating populations | Minimum yearly requirement, Kg of barley | 360 kg |
| **3.1, 3.2, and 3.6** | Calculating populations | Maximum yearly requirement, Kg of barley | 510 kg |
| **3.1** | Rough outputs | Rainfall-derived outputs | 12 kg/ha/mm |
| **3.4** | Calculating area that could be irrigated | Rainfall growing period | Calculated in step 3.8 |
| **3.4** | Calculating area that could be irrigated | Efficiency of water collection Jebel Abyad | 15% |
| **3.4** | Calculating area that could be irrigated | Efficiency of water collection Wadi Abyad | 5% |
| **3.4** | Calculating area that could be irrigated | Efficiency of water collection Wadi Djihar | 5% |
| **3.4** | Calculating area that could be irrigated | Efficiency of water collection Bir Arak | 30% |
| **3.4** | Calculating area that could be irrigated | Capacity Harbaqah | 5.000.000 m3 |
| **3.4** | Calculating area that could be irrigated | Area that can be irrigated at 200 mm in the Oasis | 2.000 ha |
| **3.7** | Workforce requirements | Harvest days | 56 |
| **3.7** | Workforce requirements | Days/ha harvest | 5,62 |
| **3.7** | Workforce requirements | Threshing kg/day | 122,92 |
| **3.7** | Workforce requirements | Winnowing kg/day | 262,5 |
| **3.8** | Estimating rainfall levels | 30-year average yearly rainfall in Palmyra | 117 mm (data from WorldClim) |

**References**

1. FAO, IIASA, ISRIC, ISS-CAS, JRC. Harmonized World Soil Database (version 1.2) [Internet]. Rome, Italy; and Laxenburg, Austria: FAO and IIASA; 2012 [cited 2021 May 17]. Available from: https://www.fao.org/soils-portal/soil-survey/soil-maps-and-databases/harmonized-world-soil-database-v12/en/

2. GADM. Country administrative areas (boundaries) of Syria (version 1.0) [Internet]. 2015 [cited 2020 Nov 11]. Available from: https://www.diva-gis.org/gdata

3. Fick SE, Hijmans RJ. WorldClim 2: New 1‐km Spatial Resolution Climate Surfaces for Global Land Areas. Int J Climatol. 2017;37(12):4302–15.

4. CNES. SPOT Controlled Image Base 10 meter (CIB-10) [Internet]. USGS. 1995 [cited 2021 May 21]. Available from: https://doi.org/10.5066/F7DB809V

5. Steduto P, Hsiao TC, Raes D, Fereres E. AquaCrop—The FAO Crop Model to Simulate Yield Response to Water: I. Concepts and Underlying Principles. Agron J. 2009;101(3):426–37.

6. Raes D, Steduto P, Hsiao TC, Fereres E. AquaCrop—the FAO Crop Model to Simulate Yield Response to Water: II. Main Algorithms and Software Description. Agron J. 2009;101(3):438–47.

7. Raes D, Steduto P, Hsiao TC, Fereres E. FAO Crop-water Productivity Model to Simulate Yield Response to Water. AquaCrop, Version 6.0 – 6.1. Reference Manual. [Internet]. Rome: Food and Agriculture Organization of the United Nations; 2018. Available from: http://www.fao.org/aquacrop/resources/referencemanuals/en/

8. Brunel JP, Ihab J, Droubi AM, Samaan S. Energy Budget and Actual Evapotranspiration of an Arid Oasis Ecosystem: Palmyra (Syria). Agr Water Manage. 2006;84(3):213–20.

9. Fischer G, Nachtergaele F, Prieler S, Van Velthuizen HT, Verelst L, Wiberg D. Global Agro-ecological Zones Assessment for Agriculture (GAEZ 2008). Laxenburg, Austria; and Rome, Italy: IIASA and FAO; 2008.

10. Armstrong R, Fitzpatrick J, Rab M, Abuzar M, Fisher P, O’Leary G. Advances in Precision Agriculture in South-eastern Australia. III. Interactions Between Soil Properties and Water Use Help Explain Spatial Variability of Crop Production in the Victorian Mallee. Crop Pasture Sci. 2009;60(9):870–84.

11. Sadras VO, McDonald G. Water Use Efficiency of Grain Crops in Australia: Principles, Benchmarks and Management. Adelaide: Grains Research and Development Corporation, South Australian Research and Development Institute and University of Adelaide; 2012 p. 28.

12. Meyer JC. Palmyrena. Palmyra and the Surrounding Territory from the Roman to the Early Islamic Period. Oxford: Archaeopress Publishing Limited; 2017.

13. Calvet Y, Geyer B. Barrages Antiques de Syrie. Lyon: Maison de l’Orient Méditerranéen; 1992. (Collection de la Maison de l’Orient Mediterraneen).

14. Geyer B. Harbaqa, un barrage dans la steppe. In: Denise F, Nordighian L, editors. Une aventure archéologique: Antoine Poidebard, photographe et aviateur. Marseille: Édition Paranthèses; 2004. p. 298–9.

15. Genequand D, al-As’ad W. Qasr al-Hayr al-Sharqi, travaux de la mission archéologique syrosuisse 2003-2007. Ann Archéol Arabes Syr. 2006 2007;49–50:177–94.

16. Genequand D, Moaz A al-Razzaq. The New Urban Settlement at Qasr al-Hayr al-Sharqi: Components and Development in the Early Islamic Period. In: Bartl K, as-Sham B, editors. Residences, Castles, Settlements Transformation Processes from Late Antiquity to Early Islam International Conference held at Damascus, 5-9 November 2006. Rahden: Marie Leidorf; 2009. p. 261–85. (Orient-Archäologie.; vol. 24).

17. Laabs J, Knitter D. How Much Is Enough? First Steps to a Social Ecology of the Pergamon Microregion. Land. 2021;10(5):479.

18. Kerig T. Towards an Econometrically Informed Archaeology: the Cologne Tableau (KöTa). In: Posluschny A, Lambers K, Herzog I, editors. Layers of Perception Proceedings of the 35th International Conference on Computer Applications and Quantitative Methods in Archaeology (CAA), Berlin, 2-6 April, 2007. Bonn: Dr. Rudolf Habelt GmbH; 2008. p. 372. (Kolloquien zur Vor- und Frühgeschichte; vol. 10).

19. Kerig T. Einfache und komplexe Wirtschaften. Studien zur Urgeschichte des Faktors Arbeit im mitteleuropäischen Neolithikum. Habilitationsschrift angenommen von der Fakultät für Geschichte, Kunst- und Orientwissenschaften der Universität Leipzig. [unpublished, Leipzig]: Universität Leipzig; 2016.

20. Lawrimore JH, Ray R, Applequist S, Korzeniewski B, Menne MJ. Global Summary of the Year (GSOY), Version 1 [Palmyra, SY] [Internet]. NOAA National Centers for Environmental Information; 2016 [cited 2021 Apr 15]. Available from: https://www.ncdc.noaa.gov/cdo-web/datasets/GSOY/stations/GHCND:SY000040061/detail
