# Supplementary material for: Food security in Roman Palmyra (Syria) in light of paleoclimatological evidence and its historical implications
Source: PLoS One. 2022 Sep 21;17(9):e0273241. doi: 10.1371/journal.pone.0273241 (PMC9491547; doi:10.1371/journal.pone.0273241)
Supplement: S4 File — It contains a short discussion on climatic proxies for the area of study. (DOCX) [file pone.0273241.s004.docx]

**Climate proxies**

In the process of conducting this study, we evaluated a few climatic proxies from the area. We prioritized proxies that reflect climate as opposed to anything else, to avoid confusions. Therefore, we did not look into pollen records.

Early on, we noticed a few problems. Firstly, there were no good proxies close to Palmyra. Additionally, there were significant disagreements between the different proxies regarding rainfall trends in the area. The explanation for these is that most of them had very low resolution, meaning that the changes observed for our period of study might have easily been a result of annual variations. While it has been possible to establish general climatic trends across centuries in the Eastern Mediterranean (1,2), we needed greater resolution.


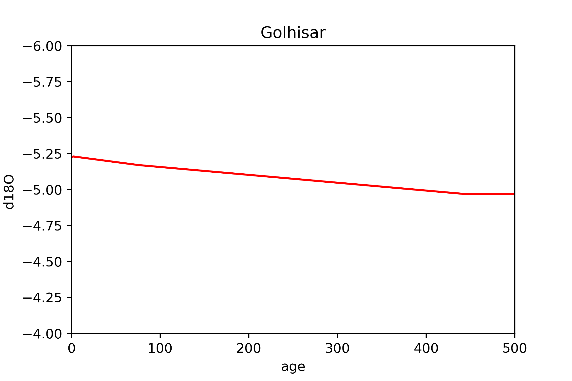

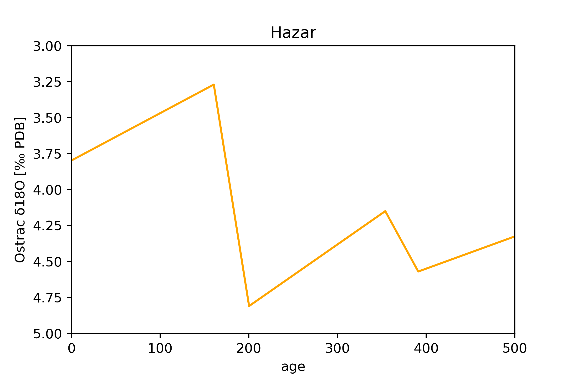

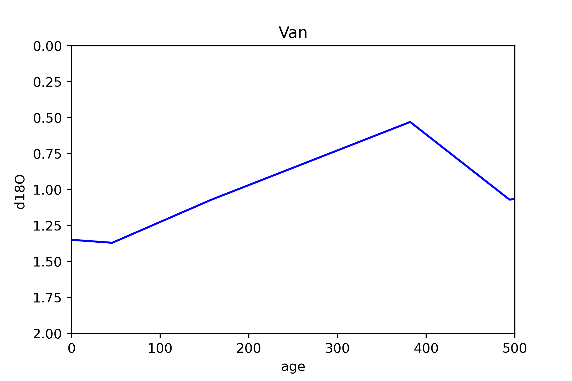

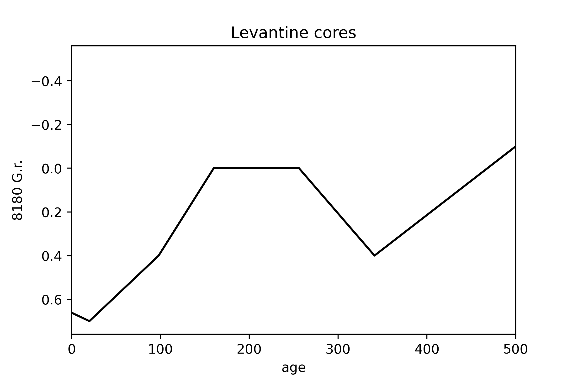


***Figure A:*** *See here for example isotope data from lake Golhisar* (3,4)*, lake Hazar* (5)*, lake Van* (6)*, and the Levantine coast* (7)*. The contradicting trends made it very difficult for us to extract any useful information.*

Circumvent this; we only took into account very high definition (sub-decadal) proxies. This forced us to look outside of the Eastern Mediterranean, in hopes of establishing large-scale trends.


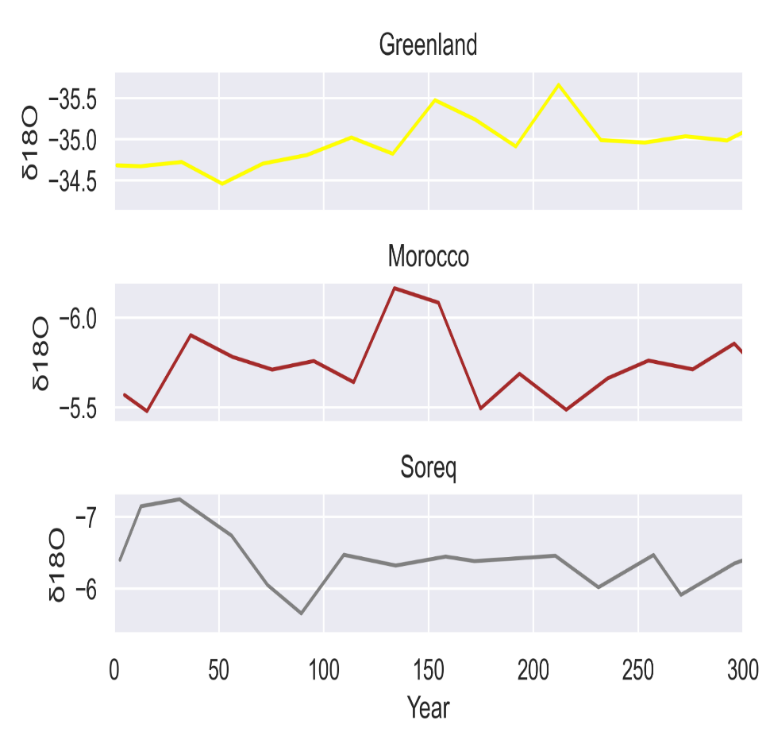


***Figure B:*** *20 year averages of Oxygen isotopes in Greenland, Morocco, and central Palestine. Data from* (8 (Soreq),9 (Morocco),10–12,13 (Greenland))*.*

These rainfall proxies, once resampled, show a variety of trends between the years 0 and AD 300. Morocco and Soreq share a smaller drop in the late first century AD, followed by a rainier 2nd c. and a drier 3rd century. However it was hard to get any representative long term trends that could be reasonably considered to apply for Palmyra.

To be able to advance, we decided to focus instead on the two best proxies available for Levantine rainfall: the one with the highest resolution and the most representative one, Soreq cave and the Dead Sea respectively.

*
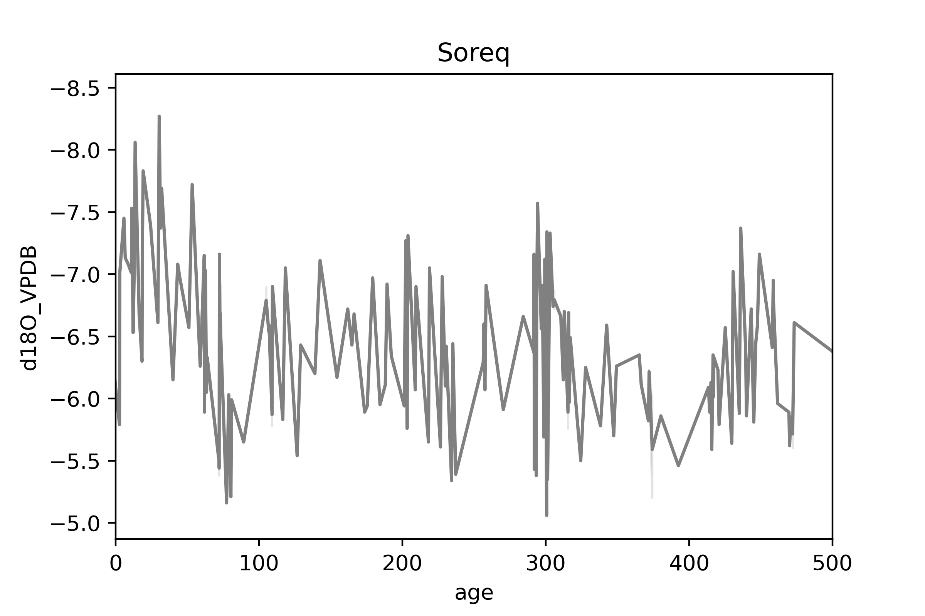
*

***Figure C:*** *Oxygen isotopes from Soreq cave, data from Orland et al. (2009).*


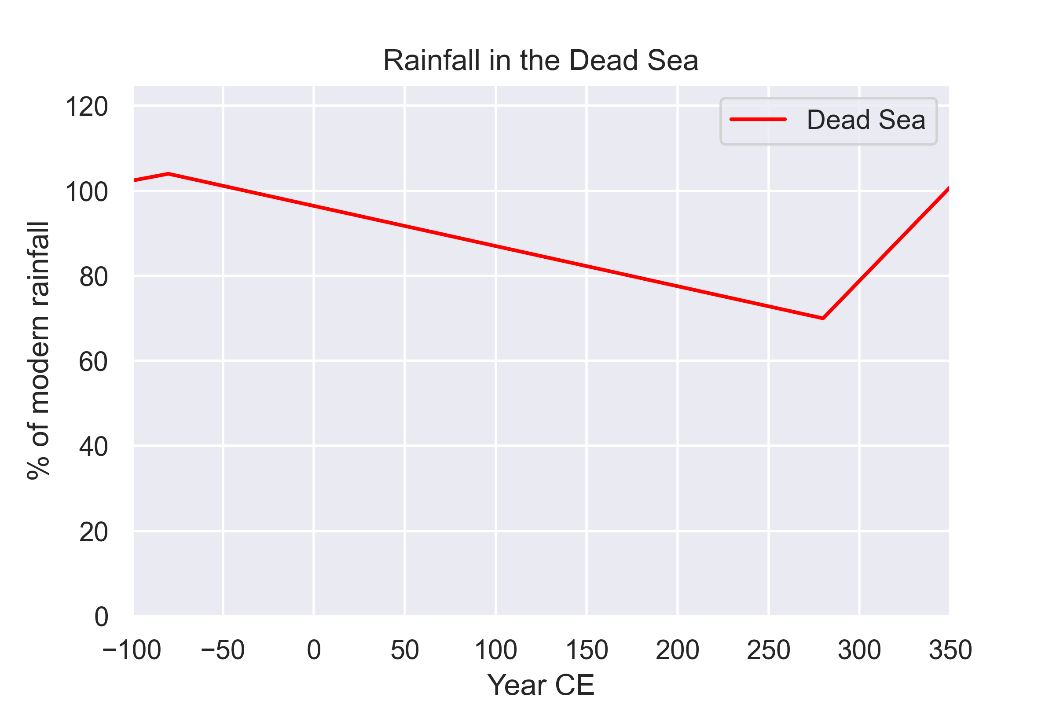


***Figure D:*** *Reconstructed average rainfalls in the Dead Sea, data from Morin et al.* [*(2019)*](https://www.zotero.org/google-docs/?NKbErT)*.*

If the overall trends were similar, then we could assume that rainfall would have followed an overall similar evolution in the interior of the Syrian Desert.

Regarding rainfall reconstructions, we also had a rainfall reconstruction from Soreq available to us. We preferred to use the one in the Dead Sea for several reasons. Firstly, the very large catchment of the Dead Sea ensures that this proxy is more representative of regional conditions than a single cave. Secondly, the lower resolution of the record is not a large issue, since the formation of ancient shorelines was not nearly as affected by yearly variation as Oxygen isotopes. Lastly, the method for rainfall reconstructions employed by Morin et al. (14) appeared more robust. For Soreq cave, a simple extrapolation from modern-day oxygen isotope levels was employed. Recently, doubts have been raised regarding the reliability of Oxygen isotopes for rainfall reconstructions, since changes in the chemical characteristics of rainwater itself can influence values (15). Seeing the wide variability of isotope values across the evaluated proxies, it seems clear that the context affects the final values significantly.

The proxies available to us are clearly not ideal to evaluate rainfall patterns around ancient Palmyra. Yet, the overall trend of decreasing rainfall during the city’s existence is clear. However results are provisional, based in the data available at the moment. New paleoclimatic proxies from Palmyra or its surroundings are needed.

Until then, we used the Dead Sea rainfall reconstruction as a basis to get an idea of how much rainfall changed in Antiquity. We did not assume that the rainfall was the same in Palmyra as it was in the areas surrounding the Dead Sea. Instead, we assumed that rainfall changed proportionally in Palmyra. Reality must have been more complicated, of course, but this is our best guess based on the available data. This means that we applied the change in rainfall in the Dead Sea to Palmyrene rainfall averages (16). With these rainfall levels, we calculated how much people could have been fed, and plotted it on a graph.

At all times, we kept in mind that the limitations of agriculture in the area and the significant variability in rainfall mean that storage of surpluses in above-average years could not compensate below-average years. Therefore, the amount of people that could be fed on average years (so, 5 out of 10 years) would have been the upper limit of the land’s carrying capacity. The low end of the standard deviation for rainfall variability, or 83 mm of rainfall, could be expected to fall 9 out of 10 years. Therefore, the actual amount of people that could be fed reliably fell somewhere between these two extremes.

# **Bibliography**

1. Finné M, Holmgren K, Sundqvist HS, Weiberg E, Lindblom M. Climate in the Eastern Mediterranean, and Adjacent Regions, During the Past 6000 Years–A Review. J Archaeol Sci. 2011;38(12):3153–73.

2. Izdebski A, Pickett J, Roberts N, Waliszewski T. The Environmental, Archaeological and Historical Evidence for Regional Climatic Changes and their Societal Impacts in the Eastern Mediterranean in Late Antiquity. Quaternary Sci Rev. 2016;136:189–208.

3. Eastwood WJ, Leng MJ, Roberts N, Davis B. Holocene Climate Change in the Eastern Mediterranean Region: a Comparison of Stable Isotope and Pollen Data from Lake Gölhisar, Southwest Turkey. J Quaternary Sci. 2007;22(4):327–41.

4. Roberts N, Jones MD, Benkaddour A, Eastwood WJ, Filippi ML, Frogley MR, et al. Stable Isotope Records of Late Quaternary Climate and Hydrology from Mediterranean Lakes: the ISOMED Synthesis. Quaternary Sci Rev. 2008;27(25–26):2426–41.

5. Ön ZB, Akçer-Ön S, Özeren MS, Eriş KK, Greaves AM, Çağatay MN. Climate Proxies for the Last 17.3 ka from Lake Hazar (Eastern Anatolia), Extracted by Independent Component Analysis of μ-XRF Data. Quaternary Int. 2018;486:17–28.

6. Wick L, Lemcke G, Sturm M. Evidence of Lateglacial and Holocene Climatic Change and Human Impact in Eastern Anatolia: High-Resolution Pollen, Charcoal, Isotopic and Geochemical Records from the Laminated Sediments of Lake Van, Turkey. The Holocene. 2003;13(5):665–75.

7. Schilman B, Bar-Matthews M, Almogi-Labin A, Luz B. Global Climate Instability Reflected by Eastern Mediterranean Marine Records during the Late Holocene. Palaeogeogr Palaeocl. 2001;176(1–4):157–76.

8. Orland IJ, Bar-Matthews M, Kita NT, Ayalon A, Matthews A, Valley JW. Climate Deterioration in the Eastern Mediterranean as Revealed by Ion Microprobe Analysis of a Speleothem that grew from 2.2 to 0.9 ka in Soreq Cave, Israel. Quaternary Res. 2009;71(1):27–35.

9. Ait Brahim Y, Wassenburg J, Sha L, Cruz F, Deininger M, Sifeddine A, et al. North Atlantic Ice‐rafting, Ocean and Atmospheric Circulation During the Holocene: Insights from Western Mediterranean Speleothems. Geophys Res Lett. 2019;46(13):7614–23.

10. Grootes PM, Stuiver M, White JWC, Johnsen SJ, Jouzel J. Comparison of Oxygen Isotope Records from the GISP2 and GRIP Greenland Ice Cores. Nature. 1993;366:552–4.

11. Johnsen SJ, Clausen HB, Dansgaard W, Gundestrup NS, Hammer CU, Andersen U, et al. The d18O Record along the Greenland Ice Core Project Deep Ice Core and the Problem of Possible Eemian Climatic Instability. J Geophys Res. 1997;102(C12):26397–410.

12. Dansgaard W, Johnsen SJ, Clausen HB, Dahl-Jensen D, Gundestrup NS, Hammer CU, et al. Evidence for General Instability of Past Climate from a 250 kyr Ice-core Record. Nature. 1993;364:218–20.

13. Anklin M, Barnola JM, Beer J, Blunier T, Chappellaz J, Clausen HB, et al. Climate Instability During the Last Interglacial Period Recorded in the GRIP Ice core. Nature. 1993;364:203–7.

14. Morin E, Ryb T, Gavrieli I, Enzel Y. Mean, Variance, and Trends of Levant Precipitation over the Past 4500 Years from Reconstructed Dead Sea Levels and Stochastic Modeling. Quaternary Res. 2019;91(2):751–67.

15. Chen J, Rao Z, Liu J, Huang W, Feng S, Dong G, et al. On the Timing of the East Asian Summer Monsoon Maximum During the Holocene—Does the Speleothem Oxygen Isotope Record Reflect Monsoon Rainfall Variability? Sci China Earth Sci. 2016;59(12):2328–38.

16. Lawrimore JH, Ray R, Applequist S, Korzeniewski B, Menne MJ. Global Summary of the Year (GSOY), Version 1 [Palmyra, SY] [Internet]. NOAA National Centers for Environmental Information; 2016 [cited 2021 Apr 15]. Available from: https://www.ncdc.noaa.gov/cdo-web/datasets/GSOY/stations/GHCND:SY000040061/detail
